# Data-driven prediction of micro-piled raft load–settlement using machine learning and Monte Carlo simulation

**DOI:** 10.1038/s41598-026-54119-6

**Published:** 2026-05-26

**Authors:** Mahmoud El Gendy

**Affiliations:** Department of Civil Engineering, Faculty of Engineering, Port Said University, Port Said, Egypt

**Keywords:** Artificial intelligence, Machine learning, Micro-piled raft, Load-settlement, Deep foundations, Probabilistic analysis, Engineering, Mathematics and computing

## Abstract

This study investigates the load–settlement behavior of micro-piled raft foundations in clay, focusing on key factors such as raft and micro-pile geometry and critical soil properties. A comprehensive dataset comprising 480 experimental records. sourced from both small-scale laboratory and large-scale field tests. was used to evaluate the predictive capabilities of six supervised machine learning algorithms: Gaussian process regression (*GPR*), extreme gradient boosting (*XGBoost*), gradient boosting machine (*GBM*), random forest (*RF*), K-nearest neighbors (*KNN*), and support vector regression (*SVR*). Each model was optimized using Bayesian optimization with 5-fold cross-validation to ensure robust performance. Model evaluation was conducted using statistical metrics, visual diagnostics (predicted-versus-actual plots), Regression error characteristics curves, score analysis, and hyperparameter tuning. Among the tested models, *GPR* demonstrated superior accuracy and generalization, effectively capturing the nonlinear soil–structure interaction typical of micro-piled raft foundations. Probabilistic analysis using Monte Carlo simulations, incorporating realistic variability in geometric and geotechnical input parameters, further validated the model’s robustness. The close agreement between predicted and experimental load–settlement responses, along with consistently narrow 95% confidence intervals, confirms the reliability of *GPR*. These findings position *GPR* as a highly promising approach for practical geotechnical design applications. Future research should focus on expanding dataset diversity, improving parameter independence, and applying advanced tuning techniques to further enhance model reliability and applicability to full-scale foundation systems.

**Supplementary Information:**

The online version contains supplementary material available at 10.1038/s41598-026-54119-6.

## Introduction

Micro-piled rafts have gained significant popularity as a deep foundation solution in civil engineering, particularly for projects with restricted access, complex ground conditions, or requiring the strengthening of existing structures^1,2^. Despite the intricate load-bearing behavior influenced by soil–structure interaction, group effects, load transfer, and construction methods, micro-piles offer notable advantages^3^. A key challenge lies in accurately predicting their response, often compounded by the misconception that their small diameter limits their load capacity. This underscores the critical need for reliable and efficient prediction tools to enable engineers to design cost-effective and high-performing micro-piled raft foundations.

The increasing adoption of micro-piles is attributed to their straightforward installation, high load capacity (especially pressure-grouted types), versatility in various soils, good penetration, and efficiency in confined spaces^4^. They are characterized by minimal settlement, good groutability, low headroom requirements, and reduced installation noise and vibration. Typically, micro-piles have a diameter under 30 cm and a depth of up to 60 m, constructed using neat cement grout and significant cast-in-place reinforcement based on design^3^. Functionally, they serve as Case I for direct load bearing and Case II for soil stabilization^3,5^, with four grouting methods defining their types: A (gravity), B (low pressure), C (gravity followed by high pressure), and D (gravity followed by multiple high-pressure injections)^3,5^.

While initially used to enhance the bearing capacity of existing shallow foundations, recent research highlights their valuable use in new construction as well^2,6^. For improving weak, deep clay, a micro-piled raft foundation can effectively increase bearing capacity and reduce settlement^7^, a phenomenon linked to soil’s nonlinear stress–strain behavior. The steel-grout area ratio is crucial for micro-pile bearing capacity and settlement^8^. Consequently, various analysis and design methods are available for piled raft and micro-piled raft foundations^9–11^. Research on piled raft settlement has frequently employed the finite element method^12–15^. Furthermore, studies utilizing small-scale models, numerical simulations, and analytical methods have assessed the settlement and seismic behavior of individual and grouped piles, as well as piled rafts, in layered clayey and sandy soils^16–18^. Investigations into the performance of micro-piled rafts in clayey or sandy soil have also been conducted using centrifuge modeling and numerical analysis^19^. As a result, both analytical and numerical methods are extensively used in the field. A common simplification involves modeling the soil-pile interaction with independent Winkler springs distributed along the pile shaft^20–24^. This method forms the basis for analyzing vertical loads using t-z curves^25–27^ and lateral loads using p-y curves^28–32^. Consequently, a variety of analysis and design techniques for piled raft foundations have been developed^33,34^.

Accurately predicting the performance of geotechnical structures such as retaining walls, slopes, and piled-raft foundations remains a fundamental challenge in civil engineering, essential for ensuring both serviceability and ultimate stability. The inherent variability of soil properties, along with the nonlinear nature of soil–structure interaction, poses significant challenges for conventional deterministic design methods. This has motivated the transition toward probabilistic, stochastic, and data-driven modeling approaches. Early contributions pioneered the use of t–z models coupled with Monte Carlo simulation to quantify uncertainty in micropile pullout behavior and develop serviceability resistance factors for LRFD-based design^34,35^. Building on this foundation, probabilistic analyses were extended to optimize the calculated resistance of driven micropiles^36^, and methods were refined for nongrouted micropile foundations^37^. Recent developments have incorporated spatial variability and slope geometry effects into reliability analyses of micro-pile-reinforced slopes using advanced stochastic techniques and Monte Carlo simulation^38^.

Parallel advances in reliability engineering have focused on CPT-based methods, partial factor frameworks, and site-specific reliability tools, including Bayesian and Markov chain Monte Carlo approaches^39–46^. Key contributions include evaluating performance and safety factors for driven piles^47^, improving CPT predictions with pore pressure considerations^48^, developing site-specific reliability tools^49^, quantifying shaft grouting benefits^50^, and establishing partial factor design frameworks^51^. Sophisticated probabilistic and statistical techniques emphasize the influence of soil variability^52^, and reliability assessments using FOSM and FORM confirm the robustness of CPT-based designs and aid LRFD calibration^53^. Innovations such as two-dimensional cloud models for risk assessment and advanced spatial variability analysis further highlight the importance of optimized site investigation^54,55^.

In parallel, evolutionary and symbolic regression approaches were applied to pile and retaining-wall problems^56,57^, and stochastic finite-element applications have been used to improve predictive performance^58^. The incorporation of Artificial Intelligence (AI), particularly Machine Learning (ML) and Deep Learning (DL), has significantly advanced geotechnical engineering in recent decades^59–61^. Among its various applications, pile foundation analysis has emerged as a key area where AI demonstrates strong potential^62–66^.

Early studies primarily concentrated on predicting the ultimate axial capacity of piles, a fundamental parameter in deep foundation design^67,68^. However, there is a growing shift toward predicting the full load–settlement curve, which offers deeper insight into both the ultimate limit state and serviceability limit state^68–70^. This trend allows for a more comprehensive assessment of pile behavior across a range of loading conditions. At the same time, researchers are increasingly addressing the specific challenges associated with modeling bored piles^71,72^. Unlike driven piles, bored piles involve prior soil excavation before concrete placement, significantly affecting the surrounding soil structure and the soil–pile interface. These differences require specialized AI modeling approaches, highlighting the broader push to better capture the complexities of soil–pile interaction and to develop more accurate and practical design tools for engineers.

A diverse range of machine learning (ML) models is currently being explored and evaluated, including ANN, RF, GBM, GPR, KNN, SVR, and ensemble methods^73–87^. Integrating ML with optimization algorithms and explainable-AI techniques has further improved predictive performance and interpretability^88–97^. Despite these advances, a systematic comparison of machine learning architectures for predicting micro-piled raft settlement, particularly incorporating robust uncertainty analysis, remains a research gap. To address this, the present study develops and rigorously evaluates a suite of machine learning models using an extensive dataset of 480 experimental cases. Six algorithms, Gaussian Process Regression (GPR), Extreme Gradient Boosting (XGBoost), Gradient Boosting Machine (GBM), Random Forest (RF), K-Nearest Neighbors (KNN), and Support Vector Regression (SVR), are implemented and optimized via Bayesian Optimization with 5-fold cross-validation, with the goal of identifying the most accurate and generalizable predictive model^98–104^.

Recent advances in machine learning (ML), deep learning (DL), and hybrid intelligent modelling have significantly enhanced predictive capabilities for complex geotechnical engineering problems characterized by strong nonlinearity, soil–structure interaction, and parameter uncertainty. In foundation engineering, data-driven approaches are increasingly being adopted to move beyond purely deterministic formulations toward more reliable, probabilistic, and physically interpretable prediction frameworks. For example, recent studies have shown that integrating ML models with Monte Carlo simulations enables accurate and uncertainty-aware prediction of bored pile load–settlement responses, highlighting the potential of probabilistic ML frameworks for serviceability assessment^103^. Complementary comparative investigations between machine learning and deep learning models for predicting the ultimate bearing capacity of shallow foundations in cohesionless soils have further confirmed the strong generalization capability of optimized data-driven models when trained on well-curated experimental datasets^104^.

At the system level, hybrid intelligent modelling has proven effective in capturing complex load–settlement behavior in reinforced soil structures. Data-driven frameworks combining multiple learning paradigms have been successfully applied to predict and validate the load–settlement response of large-scale geosynthetic-reinforced soil abutments, demonstrating strong agreement with experimental observations^105^. Extending this line of research, machine learning has also been integrated into stochastic reliability modelling of reinforced soil foundations, emphasizing the importance of coupling high predictive accuracy with probabilistic performance assessment^106^. Beyond geotechnical applications, similar hybrid approaches that combine physics-based simulations with machine learning have been applied in other engineering domains, such as CFD-assisted learning models for estimating windage losses and heat-transfer characteristics in electrical machines, underscoring the broad applicability of such methodologies to complex physical systems^107^. Most recently, explainable artificial intelligence (XAI) techniques have been introduced to improve transparency and trust in geotechnical ML models, demonstrating that physically meaningful interpretations can be extracted even from advanced learning algorithms when applied to soil strength prediction problems^108^.

The novelty of this work is threefold: (i) it provides a systematic inter-comparison of these algorithms, identifying the best-performing machine learning model as the most accurate and generalizable model; (ii) it integrates the Bayesian-optimized the best-performing model with Monte Carlo simulation (*MCS*) to perform a comprehensive probabilistic analysis; and (iii) it validates the model’s robustness across 41 experimental load–settlement curves. By bridging advanced machine learning with probabilistic assessment, this research offers a sophisticated framework that enhances prediction accuracy, enables rigorous uncertainty quantification, and provides valuable insights for the design and risk assessment of micro-piled raft foundations.

## Methodology

This study undertakes a comprehensive investigation to develop accurate predictive models for the complex load–settlement behavior of micro-piled rafts embedded in cohesive soils. A dataset comprising 480 experimental cases was compiled from a wide range of published literature, with the detailed parameters of each experiment presented in Table A1. The primary objective is to predict the settlement (*S*_*e*_) of micro-piled raft foundations, a critical performance indicator, using advanced machine learning (*ML*) techniques. A thorough literature review was conducted to identify key input parameters influencing this behavior, which include the geometric characteristics of the raft (width *B* and thickness *t*), micro-pile configuration (diameter *D*, length *L*, spacing *S*, and total number *n*), soil strength (undrained shear strength *c*_*u*_), and the applied stress (*q*). Six well-established *ML* algorithms were implemented in the Python environment, Gaussian Process Regression (*GPR*), Extreme Gradient Boosting (*XGBoost*), Gradient Boosting Machine (*GBM*), Random Forest (*RF*), K-Nearest Neighbors (*KNN*), and Support Vector Regression (*SVR*). Each model was optimized using Bayesian Optimization combined with 5-fold cross-validation to ensure robust and generalizable performance. Based on the initial comparison, the top-performing model was selected for further probabilistic analysis. To assess prediction uncertainty arising from input variability, Monte Carlo simulations (*MCS*) were employed. The resulting predictions were visualized as load–settlement curves plotted alongside corresponding experimental data, with confidence intervals included to illustrate uncertainty. This integrated methodology enables a rigorous evaluation of both predictive accuracy and uncertainty quantification, contributing to the development of more reliable and robust tools for geotechnical design and risk assessment.

### Machine learning

Machine learning (*ML*), a fundamental branch of Artificial Intelligence (*AI*), centers on creating algorithms that learn from data to identify patterns and make predictions or decisions without explicit programming. This data-centric approach is the engine behind various *AI* applications, including image and speech recognition, predictive analytics, and autonomous systems.

Gaussian Process Regression (*GPR*) is a non-parametric Bayesian regression technique that model’s relationships in data by defining a probability distribution over functions. It uses kernel functions to capture data similarity and delivers both predictions and associated uncertainty estimates. While GPR provides flexibility and a solid probabilistic framework, it is computationally intensive, particularly for large datasets, and its performance depends on appropriate kernel selection^91^.

Extreme gradient boosting (*XGBoost*) is an optimized gradient boosting framework designed for performance and speed. It constructs decision trees sequentially using gradient descent and regularization to reduce overfitting. *XGBoost* can handle missing data, support parallel processing, and integrates well with platforms like Scikit-learn, offering scalability and high predictive accuracy across various domains^93^.

Gradient Boosting Machines (*GBM*) is a powerful ensemble technique that builds models iteratively by correcting the errors of previous models using gradient descent. It aggregates the predictions of weak learners (typically decision trees) for enhanced accuracy. Though versatile and effective for both classification and regression tasks, GBM requires careful hyperparameter tuning and can be computationally expensive for large datasets^82^.

Random Forest (*RF*) is an ensemble learning method that constructs multiple decision trees using bootstrapped data and aggregates their outputs. It introduces randomness in both data and feature selection to reduce overfitting. *RF* delivers high accuracy and includes out-of-bag (*OOB*) error estimates for model validation. However, interpretability may be limited compared to single decision trees, and large forests can demand significant computational resources^56^.

K-Nearest Neighbors (*KNN*) is a simple, instance-based algorithm used for both classification and regression. It predicts output based on the ‘k’ closest data points in the training set. While *KNN* does not require training, it can be computationally expensive during inference and sensitive to feature scaling and high-dimensional data^104^.

Support Vector Regression (*SVR*) is based on Support Vector Machines and uses kernel functions to model complex relationships. It employs an $\epsilon$-insensitive loss function and regularization to control model complexity and improve robustness to outliers. While effective, *SVR* requires careful hyperparameter tuning and may not scale well to large datasets^85^.

In this study, the six machine learning algorithms were not chosen arbitrarily; rather, I deliberately selected them to represent complementary learning paradigms with demonstrated capabilities for capturing the strong nonlinearity, feature interactions, and uncertainty intrinsic to soil–structure interaction problems^104^. Specifically, these algorithms benchmark three distinct families of machine learning approaches. First, Tree-Based Ensemble Models (Random Forest, Gradient Boosting Machine, and *XGBoost*) were selected for their strong capability to capture complex nonlinear relationships and higher-order feature interactions without requiring strict assumptions about data distribution or extensive feature scaling. Second, Instance-Based Learning (K-Nearest Neighbors) was included as a non-parametric, distance-based baseline model to assess how local similarity-based learning performs relative to more sophisticated ensemble and kernel-based techniques. Finally, Kernel-Based Methods (Support Vector Regression and Gaussian Process Regression) were chosen for their mathematically rigorous mapping of inputs into high-dimensional feature spaces. In particular, Gaussian Process Regression (*GPR*) was selected for its Bayesian formulation, which provides probabilistic predictions and intrinsic uncertainty quantification (confidence intervals), a feature of critical importance for geotechnical risk assessment and reliable engineering decision-making.

Database and Modeling.

### Input selection

**Fig. 1:**
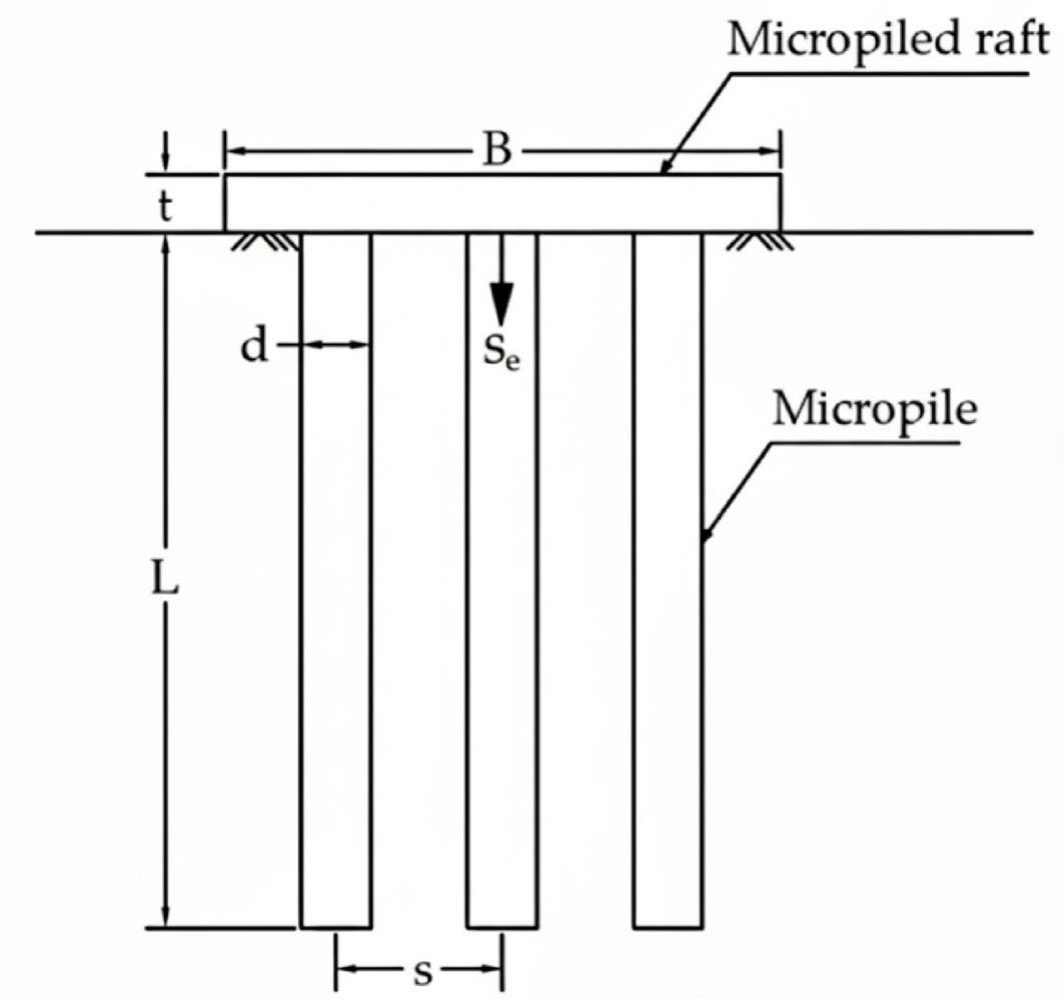
Simplified sketch of the micro-piled raft under investigation^59^.

For micro-piled raft foundations constructed on clay soils, the load–settlement behavior is primarily governed by a specific set of interrelated factors, as illustrated in Fig. 1. These include the geometric properties of the raft (its width (*B*) and thickness (*t*)) which influence its flexural rigidity and capacity to distribute loads. Equally important are the characteristics of the micro-piles: diameter (*D*) and length (*L*), which affect their axial load-bearing capacity and soil penetration depth; spacing (*S*) and total number (*n*), which control the extent and pattern of reinforcement beneath the raft.

In cohesive soils, particularly clay, the undrained shear strength (*c*_*u*_) is the most critical soil parameter. Unlike granular soils, where friction along the pile–soil interface dominates, the bearing and shaft resistance in clay is largely controlled by *c*_*u*_. This governs both the base resistance of the raft and the shaft resistance of the piles, directly impacting the foundation’s load-carrying capacity.

While natural clay deposits often exhibit broad regional variability, it is important to note that investigating the load-settlement response requires isolating specific variables. By utilizing data from experimental programs with controlled soil conditions, I can effectively minimize the variance in undrained shear strength (*c*_*u*_). This controlled approach intentionally isolates the complex influence of foundation geometry, such as raft dimensions and micro-pile configurations, and load transfer mechanisms, ensuring that the observed behavioral changes are primarily driven by the foundation’s structural arrangement rather than erratic soil heterogeneity.

Importantly, the load–settlement response of a micro-piled raft is not a simple superposition of raft and pile behaviors. Instead, it results from a complex interaction between the rigid raft, the slender micro-piles, and the surrounding clay. Factors such as pile spacing, pile length relative to the bearing stratum, and raft dimensions determine the load transfer efficiency and stress distribution within the soil. This geometric and mechanical interaction defines how load is shared between raft and piles and how the foundation deforms under applied stresses.

Given this complexity, accurate prediction of the foundation’s response requires accounting for uncertainties in key input parameters. These include spatial variability in *c*_*u*_, non-uniform load distribution among piles, and long-term effects such as consolidation. A robust uncertainty analysis must capture these sources of variability to ensure that predictive models remain reliable and reflective of real-world soil–structure interaction behavior. Accurate modeling of raft and pile geometry, soil strength, and their combined influence is essential for developing dependable geotechnical design tools.

### Dataset

For the development and validation of predictive models for micro-piled raft foundations on cohesive soils, a dataset of 480 experimental cases was compiled from^7,59^. This dataset is a composite database, including both small-scale 1 g laboratory model tests and large-scale field tests.

The dataset covers nine small-scale experiments and 32 large-scale tests. The minimum micropile diameter of 1 cm corresponds to the laboratory-scale models, where such dimensions are feasible and suitable for experimental setups. While not practical for full-scale field applications, including these scaled experiments is essential to capture the full spectrum of load–settlement behaviors. Restricting the dataset to comply strictly with FHWA standards would require excluding valid laboratory data and would alter the scope of this research. The applicability of the developed model to purely full-scale designs is addressed in the “Limitations and Future Work” section.

**Fig. 2:**
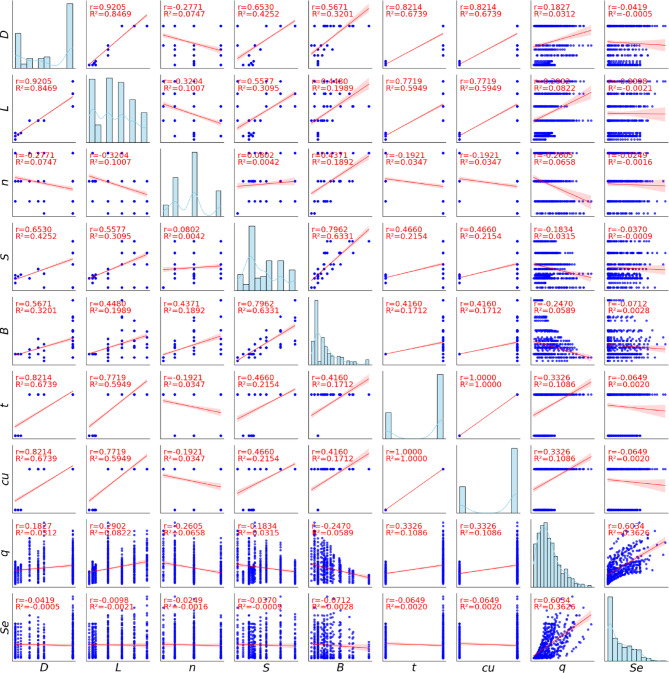
Correlation matrix for the micro-piled raft foundations databases.

Table A1 provides a comprehensive overview of literature sources and the parameter ranges for each micro-piled raft configuration. Despite variations in testing conditions, this aggregated dataset captures a broad range of simulated micro-piled raft behavior in cohesive soils, reflecting both laboratory and field scenarios. Statistical properties and distributions of the input parameters are illustrated in Fig. 2 and summarized in Table 1.

As presented in Table 1, it is essential to highlight the engineering significance of the parameter ranges, particularly the relatively narrow variation observed for the undrained shear strength (*c*_*u*_), which ranges from 16 to 19 kN/m^2^. This narrow variation directly reflects the controlled clay conditions adopted in the experimental programs from which the data were compiled. These datasets were intentionally designed to isolate the effects of foundation geometry and load transfer mechanisms under comparable, tightly controlled soil strength conditions, rather than to capture broad regional variability in clay properties. Consequently, the dataset remains physically meaningful and highly realistic for modeling the localized mechanics of micro-piled raft behavior in cohesive soils, while strictly maintaining the internal consistency and experimental control required for robust machine learning training.

**Table 1:** Statistics of input parameters.

	Min.	Max.	Mean	Std.	Skewness	Kurtosis
*D* [mm]	10	50	32.90	17.36	-0.20	-1.72
*L* [mm]	150	2000	971.32	586.34	0.25	-1.12
*n* [-]	1	16	8.24	4.54	0.36	-0.69
*S* [mm]	0	300	127.27	88.26	0.60	-0.69
*B* [mm]	100	1010	325.51	193.11	1.68	2.75
*t* [mm]	10	72.5	54.36	28.40	-0.92	-1.15
*c*_*u*_[kN/m^2^]	16	19	18.13	1.36	-0.92	-1.15
*q*[kN/m^2^]	3.61	323.24	88.81	59.90	1.04	0.91
*S*_*e*_ [mm]	0.04	89.01	19.09	18.40	0.92	0.15

To ensure the reproducibility of the machine learning workflow, the data preprocessing procedures were kept strictly transparent. Specifically, no feature normalization or scaling techniques were applied to the dataset; all continuous numerical input features were retained in their original engineering units. This approach was chosen to preserve the direct physical interpretability of the parameters. While some algorithms can be sensitive to unscaled data, the rigorous Bayesian Optimization process was utilized to explore the hyperparameter space and appropriately adjust model configurations to handle the raw numerical magnitudes. Furthermore, regarding data filtering, I deliberately chose not to apply any outlier removal techniques. The extreme values present at the tail ends of the load-settlement curves represent valid, highly non-linear physical failure mechanisms rather than erroneous measurements. Retaining these specific data points is crucial, as the predictive models must be exposed to and learn from the full spectrum of soil-structure interaction, including behavior approaching ultimate failure.

Following this structural verification, the raw dataset was partitioned into two independent subsets to ensure effective model training and unbiased performance evaluation: a training set (80% of the data) and a testing set (20%). The training set was used exclusively for model development, while the testing set, kept entirely separate during training, was reserved for final evaluation. This approach assesses the models’ generalization ability by measuring their performance on previously unseen data, thereby minimizing evaluation bias.

### Correlation analysis

**Fig. 3:**
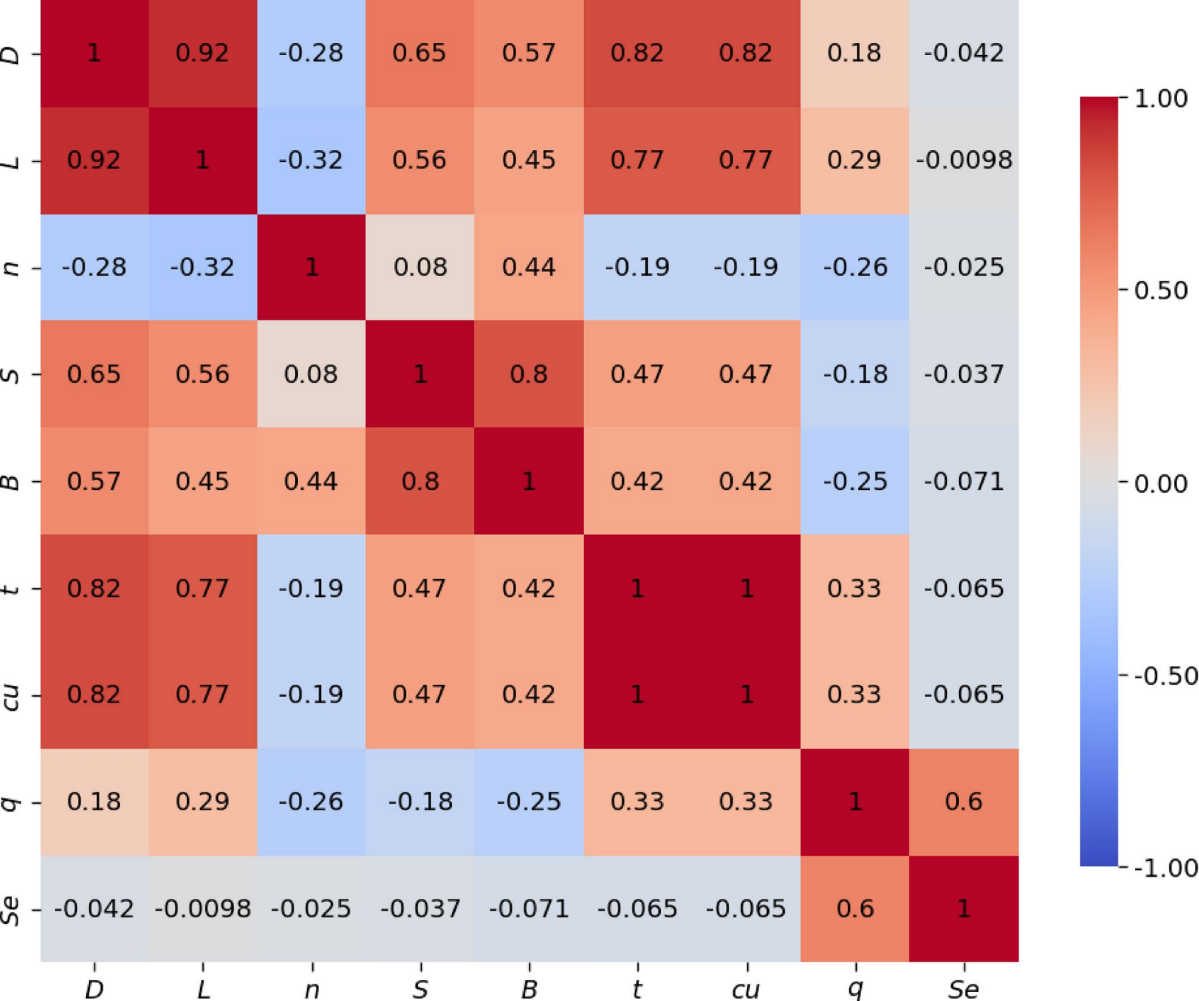
Heatmap correlation matrix.

Figure 3 presents the correlation matrix, which shows how the different input variables are linearly related to each other. Each cell in the matrix contains the Pearson correlation coefficient (*r*), which ranges from − 1 to + 1. A value of + 1 means a perfect positive relationship, -1 means a perfect negative relationship, and 0 means no linear relationship. The strength of the correlation increases as the value moves closer to either + 1 or -1. Color coding is used to make the matrix easier to interpret—red shades represent positive correlations, blue shades indicate negative correlations, and lighter colors suggest weak or no correlation.

Several strong positive correlations are observed. The micro-pile diameter (*D*) is strongly correlated with pile length (*L*) (*r* = 0.92), indicating that larger diameter piles tend to be longer. Diameter also shows strong correlations with raft thickness (*t*) and undrained shear strength (*c*_*u*_) (*r* = 0.82), suggesting that thicker rafts and stronger soils are used together with larger piles—likely for higher load capacity. Moderate correlations are also seen between diameter and pile spacing (*S*, *r* = 0.65) and raft width (*B*, *r* = 0.57).

Micro-pile length (*L*) is also positively correlated with raft thickness and *c*_*u*_ (*r* = 0.77), as well as moderately with pile spacing (*r* = 0.56) and raft width (*r* = 0.45). These trends indicate that longer piles are used with wider and thicker rafts, likely to reach stronger soil layers and carry larger loads. A strong correlation is also found between raft width and pile spacing (*r* = 0.80), meaning wider rafts tend to have more widely spaced piles.

A key finding is the perfect correlation between raft thickness (*t*) and undrained shear strength (*c*_*u*_) (*r* = 1), indicating a systematic dependency within the dataset. This likely results from consistent design practices in the original experiments, where thicker rafts were used for stronger clay deposits to ensure adequate stiffness and load distribution. Such a perfect linear relationship represents a case of extreme multicollinearity, where one variable can be exactly predicted from the other, making them redundant. While many machine learning models can tolerate moderate multicollinearity, a perfect correlation prevents the model from identifying their individual contributions. In this case, diagnostic tools like the Variance Inflation Factor (*VIF*) are unnecessary, as the result would be infinite—clearly confirming the complete redundancy of these two inputs.

Some negative correlations are also observed. For example, micro-pile diameter (*D*) and number of piles (*n*) have a moderate negative correlation (*r* = -0.28), suggesting a trade-off between using fewer large piles or more small ones. A similar pattern exists between pile length (*L*) and number of piles (*r* = -0.32). Pile spacing (*S*) also has a slight negative correlation with number of piles (*r* = -0.08), meaning more piles are generally spaced closer together.

Interestingly, the applied stress (*q*) and settlement (*S*_*e*_) show weak correlations with individual parameters like pile dimensions or soil strength. This suggests that settlement behavior is influenced by complex interactions between multiple variables, not just one. Therefore, more advanced methods like machine learning are needed to capture these nonlinear relationships.

In summary, the observed correlations generally align with geotechnical design logic. Positive correlations show expected trends (e.g., larger rafts used with stronger soil), while negative ones reflect trade-offs in design. However, the perfect correlation between *t* and *cu* should be carefully considered during modeling, as it may affect model performance. These correlations help us understand the structure of the data and support the development of reliable predictive models.

Although the correlation analysis reveals a perfect linear relationship (*r* = 1) between raft thickness (*t*) and undrained shear strength (*c*_*u*_), I deliberately retained both parameters in the input space for model training. From a geotechnical perspective, these variables represent fundamentally distinct physical domains, structural geometry and soil shear capacity, respectively. Removing one to satisfy statistical independence would result in an incomplete physical definition of the soil-structure interaction system. Therefore, both are included to preserve the physical completeness of the problem, with the explicit understanding that this severe multicollinearity will influence how the machine learning algorithms subsequently attribute predictive weight to these specific features.

### Tunning hyperparameters of machine learning models

To enhance the performance and generalizability of the machine learning models, this study employed Bayesian Optimization (BO) combined with 5-fold cross-validation (CV), forming a robust BO+5CV framework. This approach, implemented using the Optuna library, outperforms traditional methods like grid or random search by efficiently exploring the hyperparameter space. The objective was to maximize the cross-validated *R*^2^ score, ensuring the models accurately captured variance in settlement predictions. For each trial, Optuna proposed hyperparameter sets, such as the number of estimators, learning rate, and tree depth, which were evaluated through 5-fold CV. The average *R*^2^ across the five folds guided the optimizer toward optimal configurations over 1000 iterations.

To ensure the complete reproducibility and transparency of the optimization process, the specific search space boundaries defined within the Optuna framework (and the internal optimizer for GPR) are explicitly outlined in Table 2. Defining these ranges is a critical step in Bayesian Optimization, as it establishes the boundary conditions from which the objective function evaluates performance and samples subsequent configurations. As detailed in the table, the predefined search space encompassed comprehensive continuous and categorical ranges for tree-based metrics, spatial distance parameters for instance-based learning, and regularization margins for kernel-based regressions.

**Table 2:** Hyperparameter search space boundaries defined for model optimization^103,104^.

ML model	Hyperparameter
*XGBoost*	Number of Estimators (n_estimators) = [800–1200], Learning Rate (learning_rate) = [0.01–0.30], random_state = 42, n_jobs= -1
*GBM*	Number of Estimators (n_estimators) = [800–1200], Learning Rate (learning_rate) = [0.05–0.30], random_state = 42
*RF*	Number of Estimators (n_estimators) = [100–1000], Maximum Depth (max_depth) = [3–20], Min Samples Split (min_samples_split) = [2–10], Min Samples Leaf (min_samples_leaf) = [1–10], Bootstrap (bootstrap) = [True, False], random_state = 42
*KNN*	Number of Neighbors (n_neighbors) = [1–50], Weight Function (weights) = [‘uniform’, ‘distance’], Power Parameter (p) = [1 (Manhattan), 2 (Euclidean)], Algorithm (algorithm) = [‘auto’, ‘ball_tree’, ‘kd_tree’, ‘brute’]
*SVR*	Kernel (kernel) = [‘linear’, ‘poly’, ‘rbf’, ‘sigmoid’], Penalty Parameter (C) = [0.001–1000] *(Log-Uniform)*, Epsilon (epsilon) = [0.01–1.0], Polynomial Degree (degree) = [2–5]
*GPR*	Composite Kernel Elements = RBF + Matern + WhiteKernel + RationalQuadratic, Length-scale & Constant Bounds = [1e-5–1e5], Optimizer Restarts (n_restarts_optimizer) = 10

**Fig. 4:**
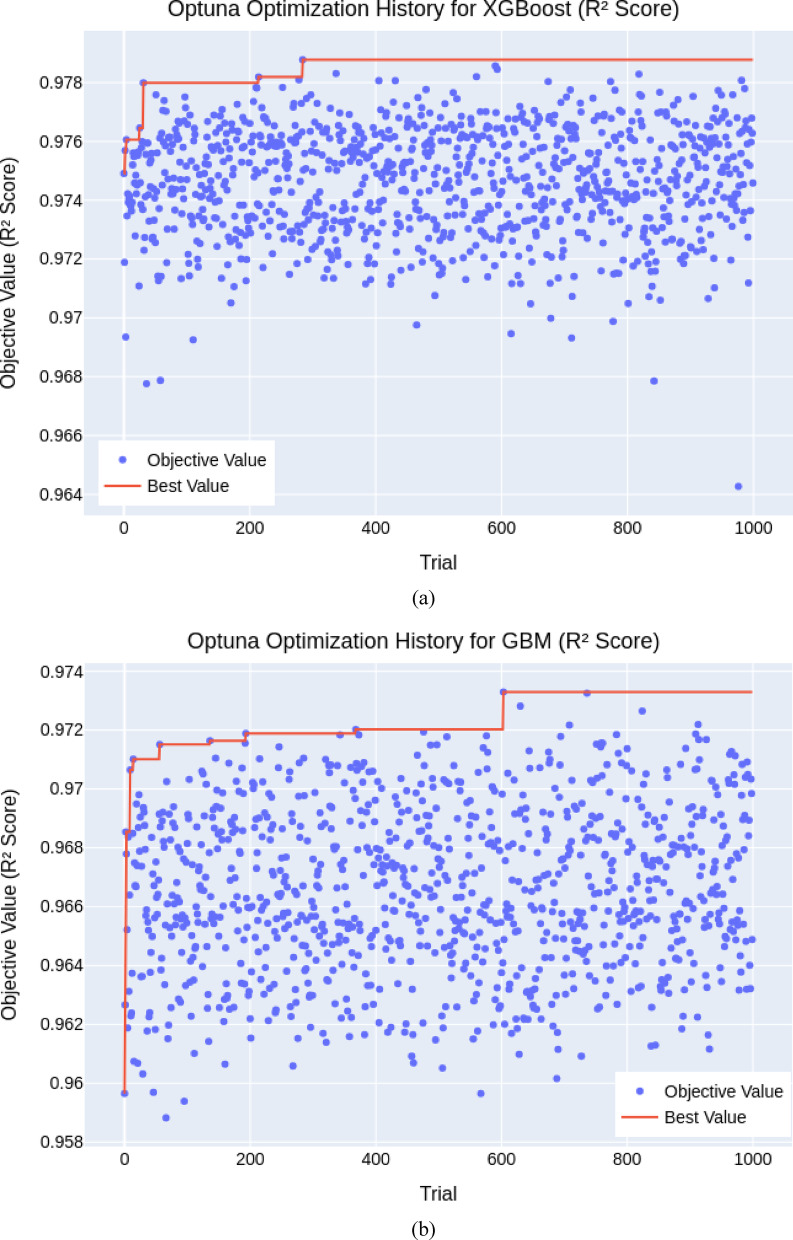

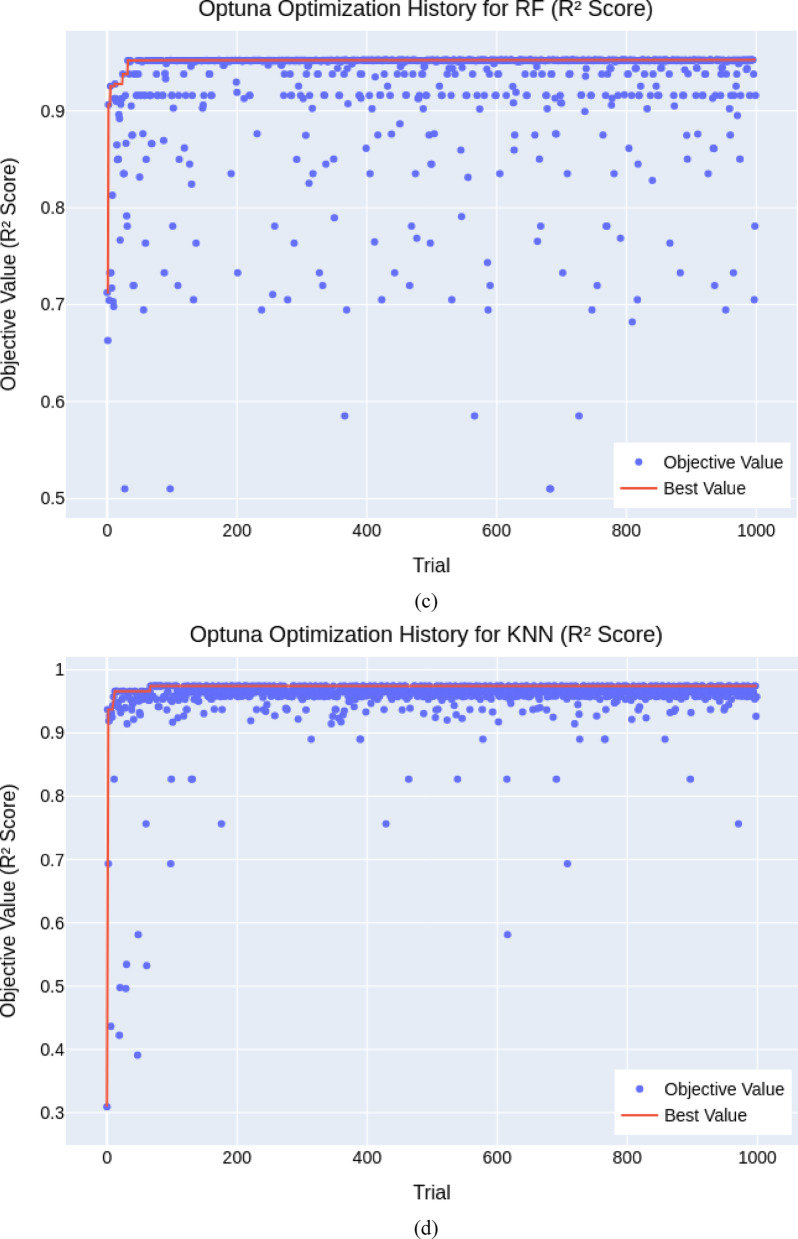

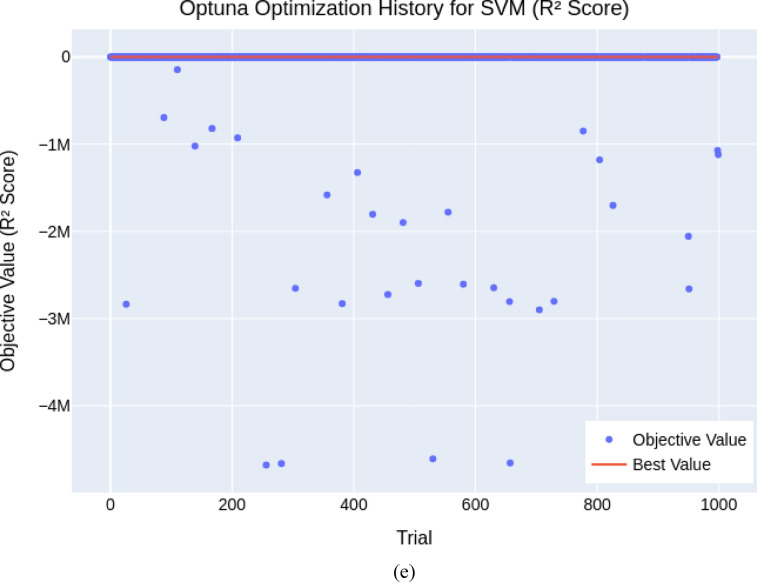
Convergence plot of the Bayesian Optimization for hyperparameter tuning (**a**) *XGBoost*, (**b**) *GBM*, (**c**) *RF*, (**d**) *KNN*, (**e**) *SVR*.

**Fig. 5:**
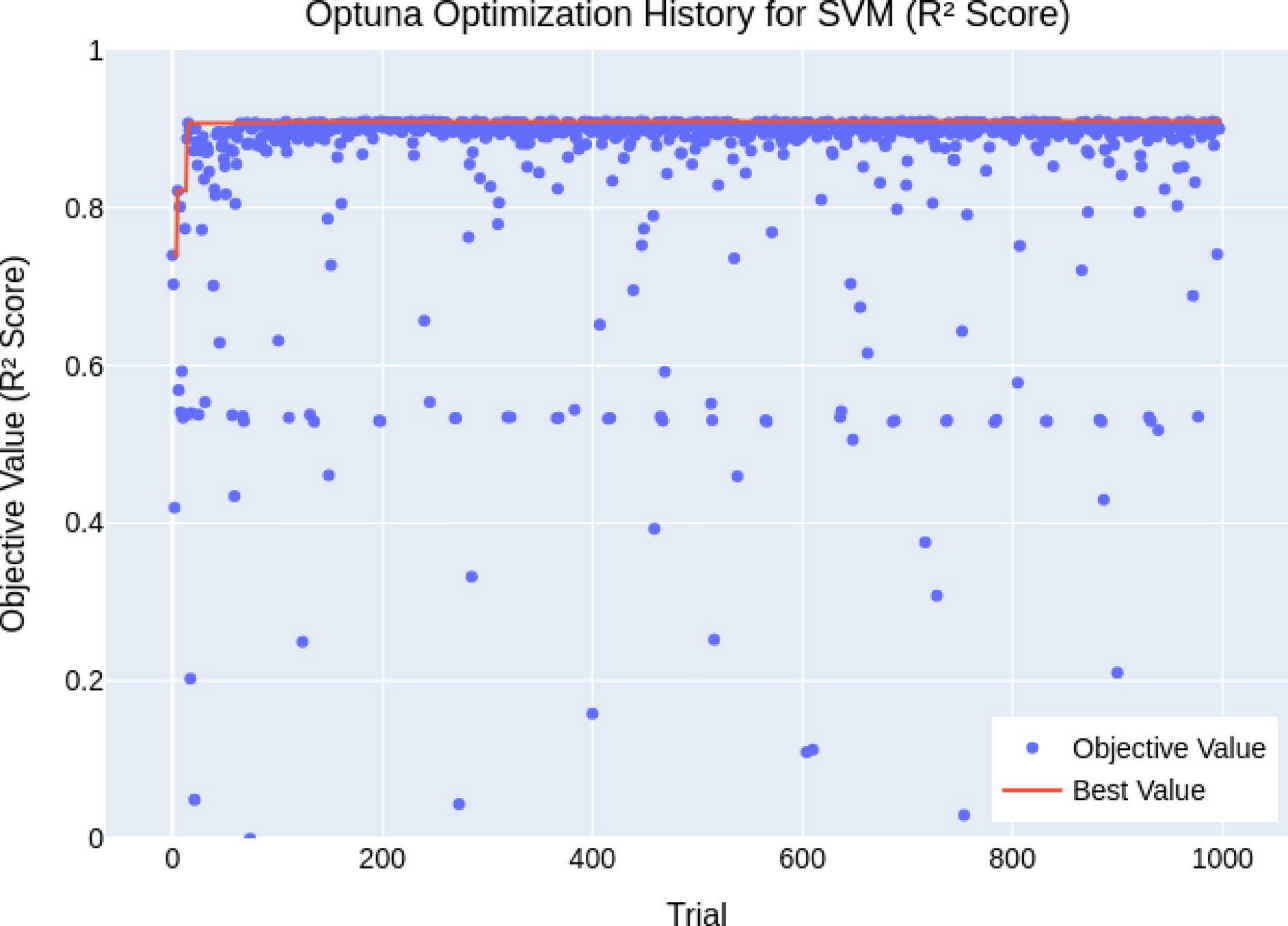
Convergence plot of the Bayesian optimization for *SVR* hyperparameter tuning (Positive value only).

The final hyperparameters (Table 3) demonstrated high model performance, particularly for boosting-based models. As illustrated in *XGBoost* (Fig. 4a) and *GBM* (Fig. 4b) showed rapid convergence and stable high *R*^2^ scores (0.979 and 0.973), indicating robustness across different hyperparameter sets. In contrast, *RF* (Fig. 4c) and *KNN* (Fig. 4d) were highly sensitive to tuning, with wide variability in performance across trials despite achieving strong peak *R*^2^ values (≈ 0.952 and 0.974). *SVR* (Figs. 4e and 5) delivered moderate consistency, but some trials yielded negative *R*^2^ scores, revealing optimization challenges and limitations in robustness. Overall, the BO+5CV strategy effectively identified high-performing configurations, especially for models like *XGBoost* and *GBM*, while highlighting stability concerns in others.

**Table 3:** ptimal hyperparameters of machine learning models.

ML model	Optimal hyperparameters
*XGBoost*	n_estimators = 827, learning_rate = 0.15009718753856716, random_state = 42, n_jobs=-1
*GBM*	n_estimators = 815, learning_rate = 0.26871296160819835, random_state = 42
*RF*	n_estimators = 231, max_depth = 14, min_samples_split = 2, min_samples_leaf = 1, bootstrap=False, random_state = 42
*KNN*	n_neighbors = 2, weights=’distance’, *p* = 2, algorithm=’auto’
*SVR*	kernel=’rbf’, C = 998.9603412068344, epsilon = 0.14934351511702545, degree = 4

**Fig. 6:**
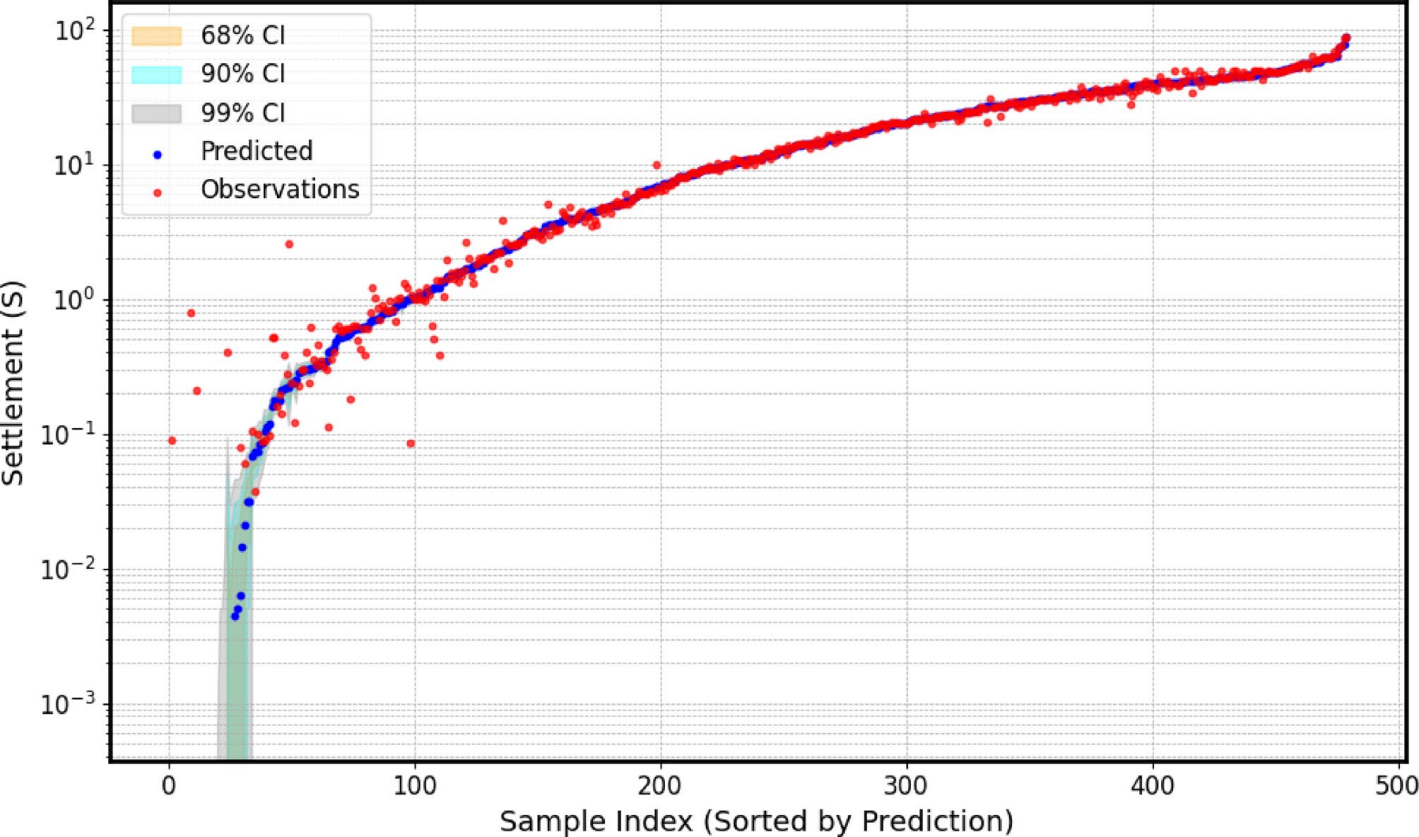
Gaussian process regression predictions with confidence intervals.

For the Gaussian process regressor (*GPR*), hyperparameter tuning focused on optimizing a composite kernel structure that integrates several covariance functions, including ConstantKernel, RBF, Matern, WhiteKernel, and RationalQuadratic. Key kernel parameters—such as constant_value, length_scale (for RBF and Matern), ν (for Matern), noise_level (for WhiteKernel), and α (for RationalQuadratic)—were optimized using skopt.gp_minimize, a Bayesian optimization method. Unlike Optuna, this framework does not generate convergence plots, so a direct visualization of the optimization process is unavailable. Instead, the model’s performance was assessed through predicted vs. actual plots, providing a clear evaluation of accuracy and generalization. Additionally, the *GPR* confidence interval plot (Fig. 6) showcases the model’s strength in capturing both trends and uncertainties in settlement predictions across a wide range, from 0.001 to 100 units, on a logarithmic scale. The nested 68%, 90%, and 99% confidence intervals widen appropriately in regions of higher uncertainty, while remaining tight where the model is more certain. Most observed data points fall within the 90% interval, indicating good calibration, although some mid-range settlements (0.1–1 unit) fall outside the 99% band, highlighting areas of increased predictive difficulty. The smooth prediction curve and well-structured uncertainty bands reflect both the model’s physical relevance and its limitations, providing engineers with accurate predictions and transparent risk bounds essential for safe and reliable geotechnical design.

### Model evaluation

Evaluating the performance of machine learning (*ML*) models in predicting the complex load–settlement behavior of micro-piled raft foundations requires a rigorous and multi-dimensional framework that extends beyond basic training accuracy. To avoid overfitting and ensure generalizability, a separate testing dataset was used to evaluate how well each model could predict unseen load–settlement conditions, reflecting realistic variability in soil and loading scenarios. Both visual and statistical evaluation methods were employed. Visual diagnostics, including scatter plots for predicted-versus-actual plots across various load levels, offered intuitive insights into model behavior, enabling the detection of bias or systematic prediction errors. Complementing these, a comprehensive suite of statistical metrics provided quantitative evaluation of each model’s predictive reliability. These included the Coefficient of Determination (*R*^2^), Mean Absolute Percentage Error (*MAPE*), Mean Absolute Error (*MAE*), Mean Bias Error (*MBE*), and Root Mean Square Error (*RMSE*), which together assessed accuracy, error magnitude, and bias. To capture curve prediction fidelity, additional metrics such as the A20 Index, Scatter Index (*SI*), and Agreement Index (*d*) were introduced. The A20 Index represents the percentage of predictions within ± 20% of the actual values, with higher values indicating better reliability. The SI normalizes the prediction error relative to observed values, offering a scale-independent measure of scatter, while the Agreement Index (ranging from 0 to 1) quantifies the overall match between predictions and observations. Higher *d*-values indicate closer alignment with real-world behavior. The mathematical formulations of these metrics were also provided for transparency and reproducibility.

To deepen the understanding of model error distribution, Regression Error Characteristic (*REC*) curves were employed, particularly for the best-performing model. *REC* curves graphically represent the cumulative distribution of prediction errors, plotting the error tolerance (x-axis) against the percentage of samples with errors less than or equal to that tolerance (y-axis). This visualization aids in assessing how quickly the model predictions converge to low error levels, providing an intuitive measure of overall error characteristics. The area over the *REC* curve (AOC) further quantifies model performance, with lower AOC values indicating better predictive accuracy. In addition, score analysis across multiple evaluation metrics was conducted to benchmark model strengths and weaknesses comprehensively. This multi-metric scoring highlighted the trade-offs between precision, bias, and generalization ability, ensuring that the chosen model balanced these factors optimally.

Crucially, explainability was introduced via SHapley Additive exPlanations (*SHAP*) analysis applied to the top-performing model, enhancing transparency and trust. *SHAP* values quantify the contribution of each input feature to individual predictions, enabling a global and local interpretability of the model. Feature importance rankings derived from *SHAP* clarified which parameters most strongly influenced load–settlement predictions, revealing key drivers such as micro-pile diameter, length, raft thickness, and undrained shear strength. Local explanations identified instances where specific features had outsized effects, providing insights into model decision-making patterns and helping diagnose potential sources of error or bias. This interpretability framework empowers engineers to better understand, validate, and trust the model outputs, facilitating informed decision-making in design and risk assessment of micro-piled raft foundations. Further enhancing top-performing model credibility, a Monte Carlo simulation-based uncertainty analysis was conducted to propagate variability in input parameters, generating probabilistic load–settlement curves with confidence intervals. This combined approach, integrating visual interpretation, statistical evaluation, and uncertainty quantification, ensures a thorough and reliable assessment of each *ML* model’s accuracy, robustness, and practical applicability in geotechnical engineering contexts.

The mathematical definitions of the evaluation metrics are presented as follows:

1 $$\:{R}^{2}=1-\frac{\sum\:{\left({y}_{i}-\widehat{y}\right)}^{2}}{\sum\:{\left({y}_{i}-\stackrel{-}{y}\right)}^{2}}$$

2 $$\:MAPE = \frac{{100}}{n}\sum\limits_{{i = 1}}^{n} {\frac{{y_{i} - \hat{y}}}{{y_{i} }}}$$

3 $$MAE = \frac{1}{n}\sum\limits_{{i = 1}}^{n} {\left| {y_{i} - \hat{y}} \right|}$$

4 $$MBE = \frac{1}{n}\sum\limits_{{i = 1}}^{n} {\left( {y_{i} - \hat{y}} \right)}$$

5 $$\:RMSE=\sqrt{\frac{1}{n}\sum\:{\left({y}_{i}-\widehat{y}\right)}^{2}}$$

6 $${\mathrm{A}}20 = \frac{1}{n}\sum\limits_{{i = 1}}^{n} {\left\{ {\begin{array}{*{20}c} {1,\:\:\:\:\:\:\:\:\:\:\:\:\:{\mathrm{if}}\:\left| {\frac{{\left( {y_{i} - \mathop y\limits^{ - } } \right)}}{{y_{i} }}} \right| \le \:0.2} \\ {\:0,\:\:\:\:\:\:\:\:\:\:\:\:\:\:\:\:otherwise\:\:\:\:\:\:\:\:\:\:\:\:} \\ \end{array} } \right.}$$

7 $$\:SI=\frac{RMSE}{\stackrel{-}{y}}$$

8 $$d = 1 - \frac{{\sum\limits_{{i = 1}}^{n} {\left( {y_{i} - \hat{y}} \right)^{2} } }}{{\sum\limits_{{i = 1}}^{n} {\left( {\left| {\hat{y} - \mathop y\limits^{ - } } \right| + \left| {y_{i} - \mathop y\limits^{ - } } \right|} \right)^{2} } }}$$

Where $\:{y}_{i}$ is the actual value, $\:\widehat{y}$ is predicted value, $\:\stackrel{-}{y}$ is the mean of the actual values, and $\:\stackrel{̿}{y}$ is the mean of the predicted values.

## Results

### Performance and results of machine learning models

**Fig. 7:**
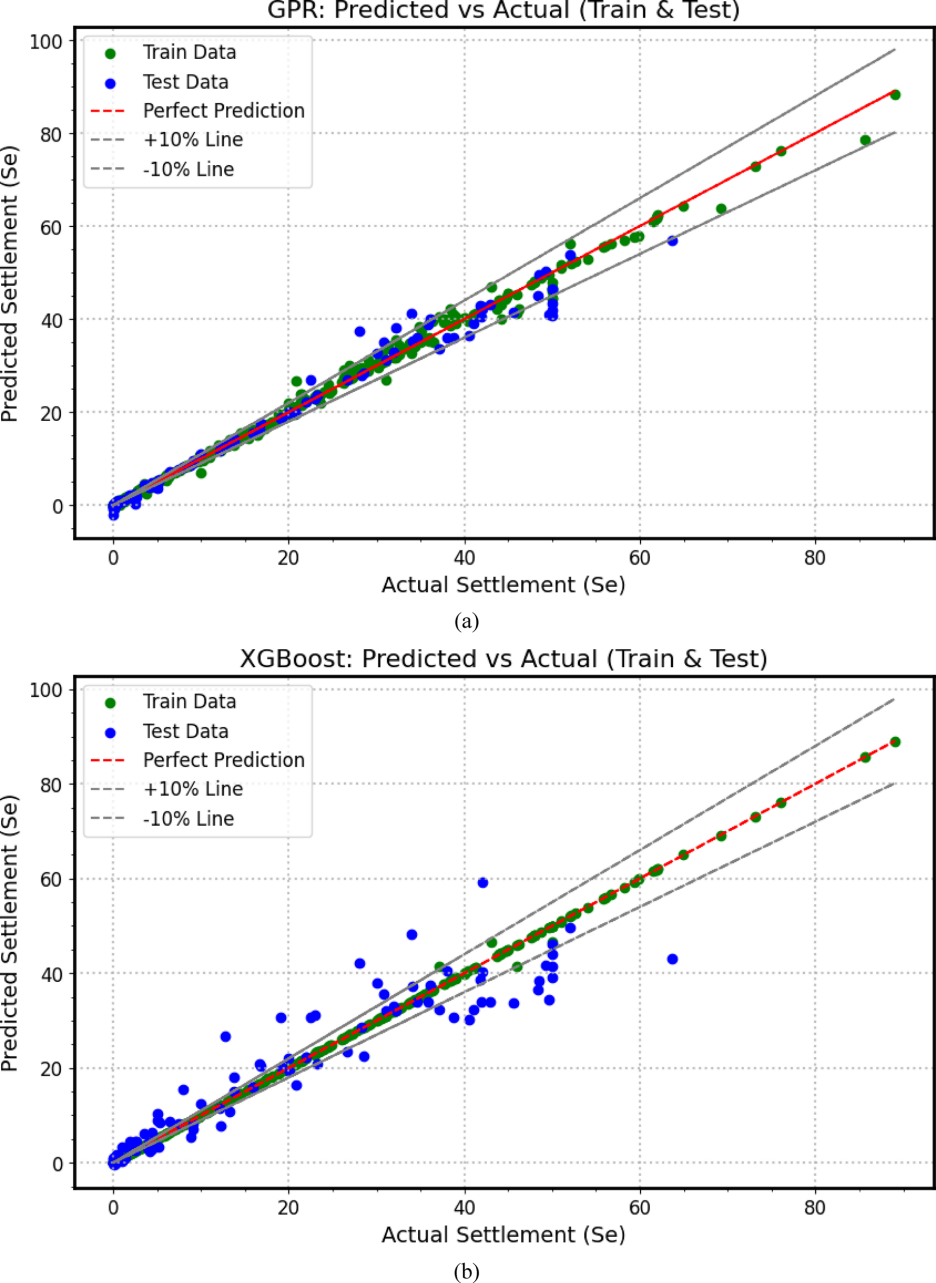

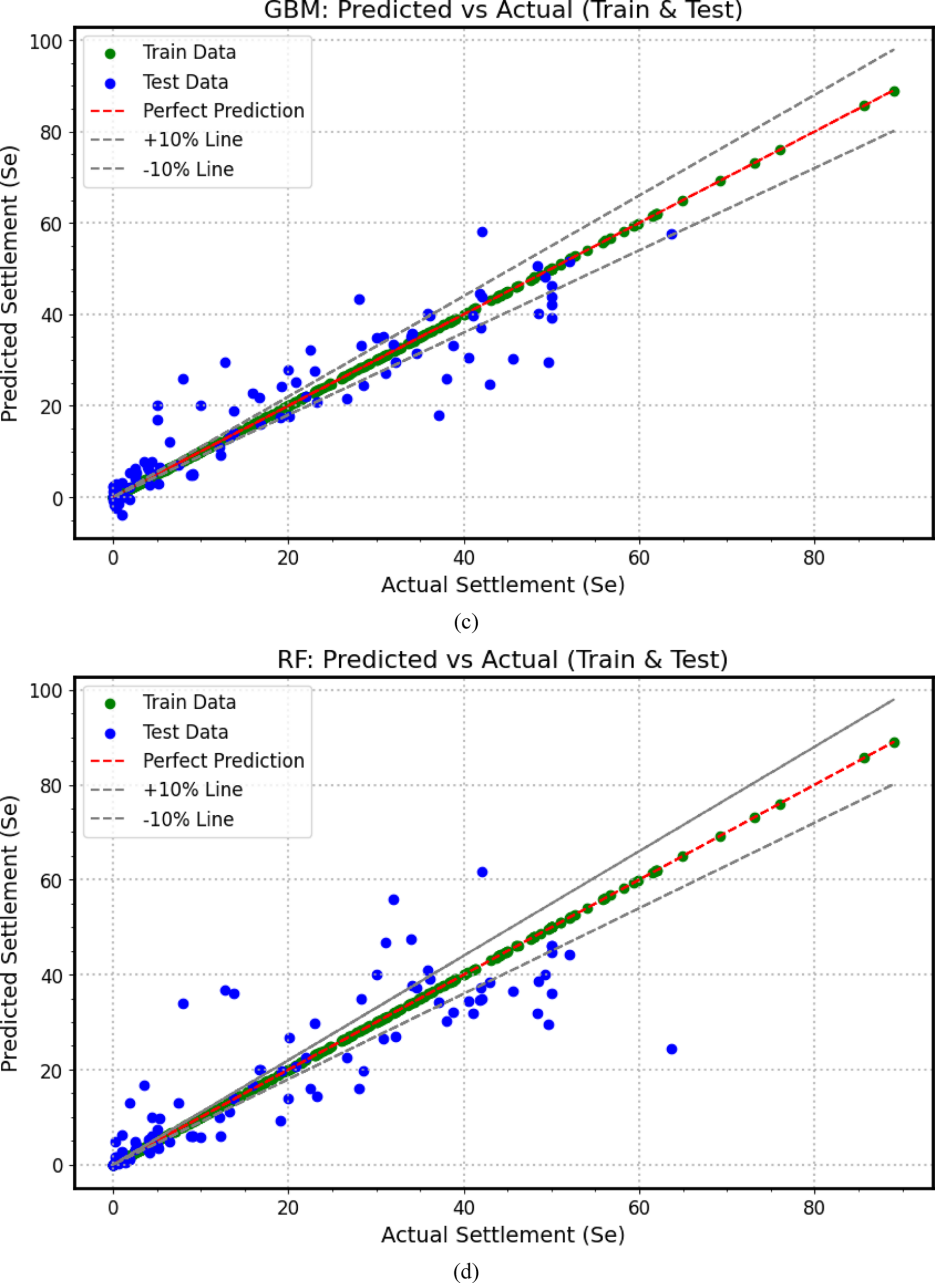

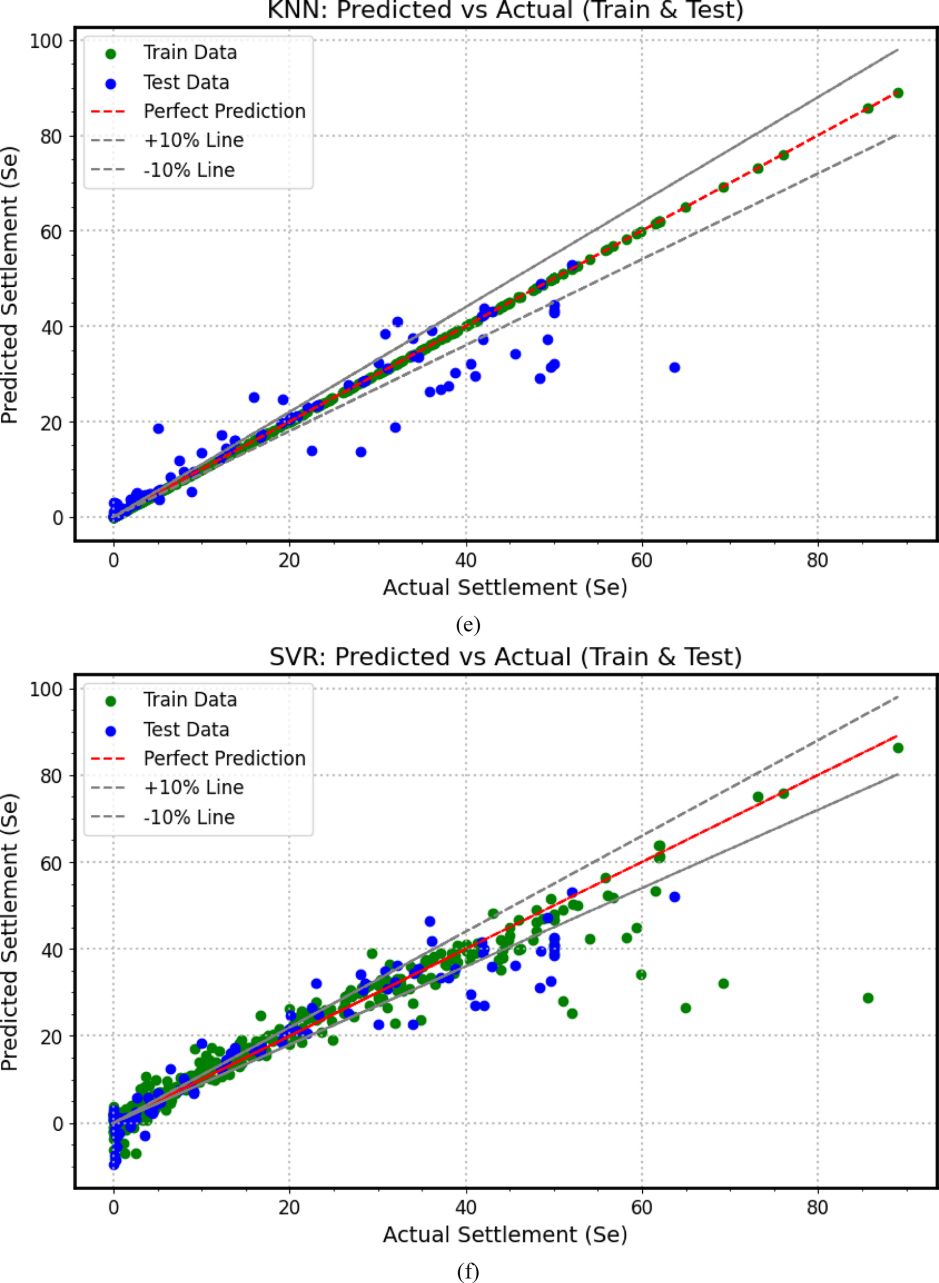
Scatter plots between actual and predicted settlement values based on (**a**) *GPR*, (**b**) *XGBoost*, (**c**) *GBM*, (**d**) *RF*, (**e**) *KNN*, (**f**) *SVR*.

**Fig. 8:**
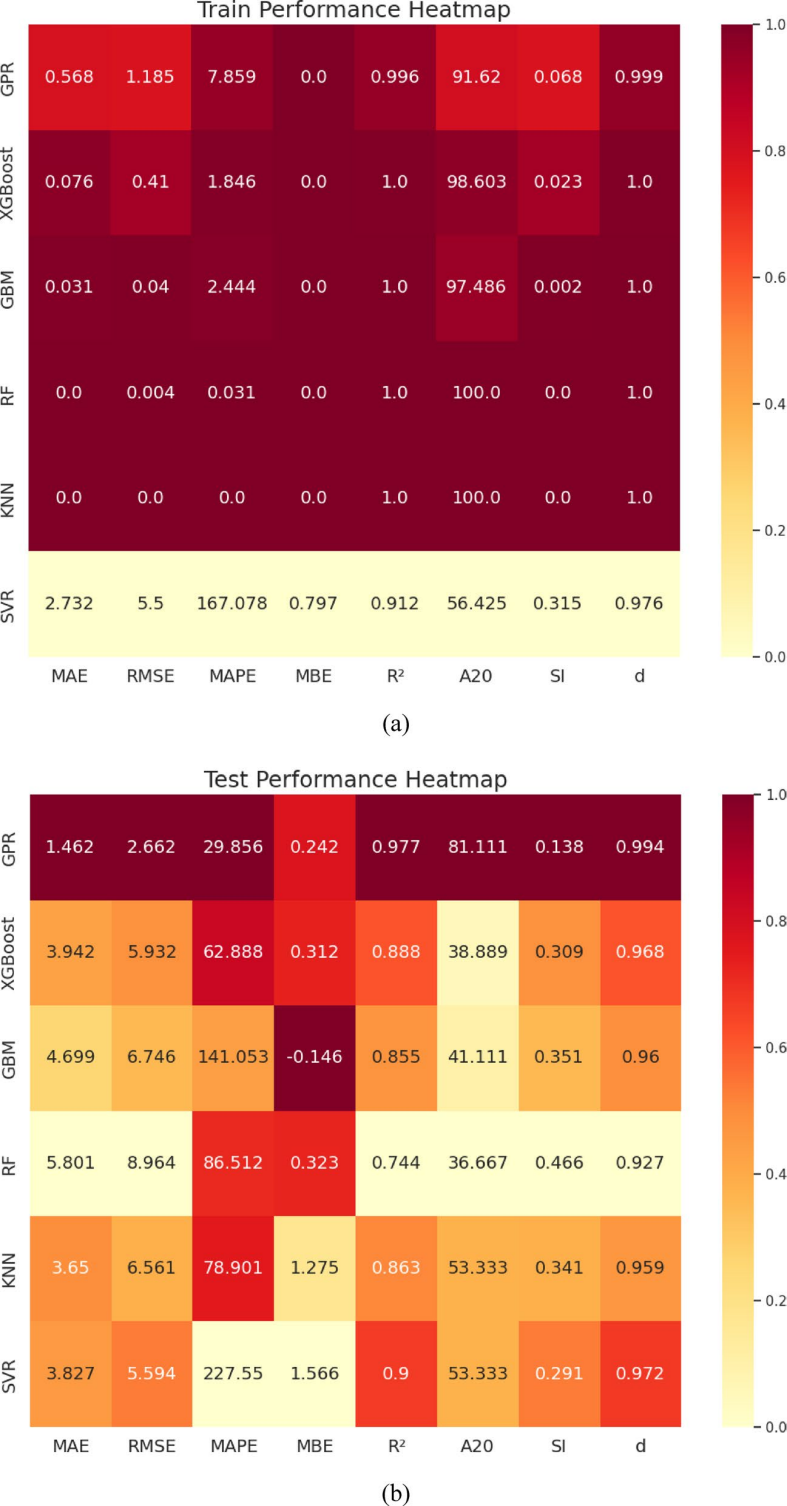
Comparison of the developed *ML* models’ performance heatmap (Darker = Better) for predicting micro-piled raft settlements (mm): (**a**) Training phase; (**b**) Testing phase.

The predictive performance of the developed machine learning (*ML*) models for load–settlement behavior of micro-piled raft foundations in clay is illustrated through scatter plots (Fig. 7). The close clustering of both training (80%) and testing (20%) data points along the diagonal line demonstrates a strong correlation between predicted and experimental values, confirming the models’ accuracy and reliability. To provide a comprehensive quantitative comparison, a performance heatmap (Fig. 8) summarizes key statistical metrics. Together, these metrics rigorously evaluate each model’s precision, bias, consistency, and generalization ability during both training and testing phases.

All *ML* models achieved high *R*^2^ values near unity and low error metrics on the training data, indicating strong learning of complex patterns. However, the heatmap in Fig. 8 reveals varying generalization performance on the testing dataset, exposing the typical challenge of overfitting where models fit training data noise but show reduced predictive robustness on unseen data. This performance hierarchy highlights the trade-off between training accuracy and real-world applicability, emphasizing the importance of robust validation for practical deployment.

Among all algorithms, *GPR* (Fig. 7a) demonstrates the most balanced and robust performance. Its testing metrics (*R*^2^ = 0.977, *MAPE* = 29.86%, *MBE* = 0.242 mm, *MAE* = 1.462 mm, *RMSE* = 2.662 mm, A20 = 81.11%, *SI* = 0.138, *d* = 0.994) (Fig. 8a) remain close to its training results (*R*^2^ = 0.996, *MAPE* = 7.86%, *MAE* = 0.568 mm, *RMSE* = 1.185 mm, A20 = 91.62%, *SI* = 0.068, *d* = 0.999) (Fig. 8b). This modest decline between training and testing indicates excellent generalization, demonstrating the model’s ability to effectively capture nonlinear soil–structure interactions without overfitting. Thus, GPR emerges as the most reliable and stable predictive tool for estimating the load–settlement response of micro-piled raft foundations.

It is important to note that while the *GPR* model’s *MAPE* of approximately 30% may seem high, this is largely due to the wide range of settlement magnitudes in the dataset, spanning from a few millimeters to tens of millimeters. Small absolute errors (*MAE* ≈ 1.5 mm, *MBE* ≈ 0.24 mm) naturally translate into higher percentage errors for smaller settlement values, inflating the *MAPE* metric. Therefore, the elevated *MAPE* reflects sensitivity to data scale rather than model inaccuracy, reaffirming *GPR’s* strong correlation and low absolute error across the dataset.

In contrast, *XGBoost* (Fig. 7b) exhibits clear signs of overfitting. While it achieved near-perfect training metrics (*R*^2^ = 0.9995, *MAPE* = 1.85%, *MAE* = 0.076 mm, *RMSE* = 0.410 mm, A20 = 98.60%, *SI* = 0.023, *d* = 0.9999) (Fig. 8a), its testing performance declined substantially (*R*^2^ = 0.888, *MAPE* = 62.89%, *MBE* = 0.312 mm, *MAE* = 3.942 mm, *RMSE* = 5.932 mm, A20 = 38.89%, *SI* = 0.309, *d* = 0.968) (Fig. 8b), indicating reduced stability and generalization on unseen data.

Similarly, *GBM* (Fig. 7c) showed excellent training results (*R*^2^ ≈ 1, *MAPE* = 2.44%, *MAE* = 0.031 mm, *RMSE* = 0.04 mm, A20 = 97.49%, *SI* = 0.002, *d* = 1) (Fig. 8a) but experienced significant drops in testing accuracy (*R*^2^ = 0.855, *MAPE* = 141.05%, *MBE* = − 0.146 mm, *MAE* = 4.699 mm, *RMSE* = 6.746 mm, A20 = 41.11%, *SI* = 0.351, *d* = 0.960) (Fig. 8b). The dramatic rise in *MAPE* and *RMSE* highlights overfitting and weak generalization despite excellent training precision.

*RF* (Fig. 7d) also demonstrates extreme overfitting. The model achieves near-perfect training metrics (*R*^2^ ≈ 1, MAPE = 0.03%, *RMSE* = 0.0039 mm, A20 = 100%, *SI* = 0.0002, *d* ≈ 1) (Fig. 8a), but its testing results drop sharply (*R*^2^ = 0.744, *MAPE* = 86.51%, *MBE* = 0.323 mm, *MAE* = 5.801 mm, *RMSE* = 8.964 mm, A20 = 36.67%, *SI* = 0.466, *d* = 0.927) (Fig. 8b). This substantial discrepancy emphasizes the model’s tendency to memorize training data, reducing its generalization capability to new soil conditions.

*KNN* (Fig. 7e) achieves a perfect fit to the training dataset (*R*^2^ = 1.0, *MAPE* = 0%, *MBE* = 0 mm, *MAE* = 0 mm, *RMSE* = 0 mm, A20 = 100%, *SI* = 0, *d* = 1.0) (Fig. 8a), indicating complete memorization of training samples. However, its testing results (*R*^2^ = 0.863, *MAPE* = 78.90%, *MBE* = 1.275 mm, *MAE* = 3.650 mm, *RMSE* = 6.561 mm, A20 = 53.33%, *SI* = 0.341, *d* = 0.959) (Fig. 8b) reveal limited generalization and higher prediction errors, demonstrating that *KNN* struggles to adapt effectively to unseen data.

Finally, *SVR* (Fig. 7f) exhibits moderate and relatively stable behavior across both phases. The training results (*R*^2^ = 0.912, *MAPE* = 167.08%, *MBE* = 0.797 mm, *MAE* = 2.732 mm, *RMSE* = 5.5 mm, A20 = 56.42%, *SI* = 0.315, *d* = 0.976) (Fig. 8a) and testing outcomes (*R*^2^ = 0.900, *MAPE* = 227.55%, *MBE* = 1.566 mm, *MAE* = 3.827 mm, *RMSE* = 5.594 mm, A20 = 53.33%, *SI* = 0.291, *d* = 0.972) (Fig. 8b) remain relatively consistent, though the large *MAPE* values and error magnitudes indicate weaker predictive precision compared to *GPR*.

In summary, the comparative heatmap (Fig. 8) and scatter plots (Fig. 7) collectively confirm that *GPR* achieves the optimal balance between accuracy, stability, and generalization. *XGBoost* and *KNN* exhibit moderate reliability, while *GBM*, *RF*, and *SVR* suffer from pronounced overfitting or instability. These findings underscore the importance of appropriate model regularization, parameter tuning, and cross-validation in developing robust, data-driven predictive tools for geotechnical engineering applications involving settlement prediction (mm).

### The regression error characteristics curve

**Fig. 9:**
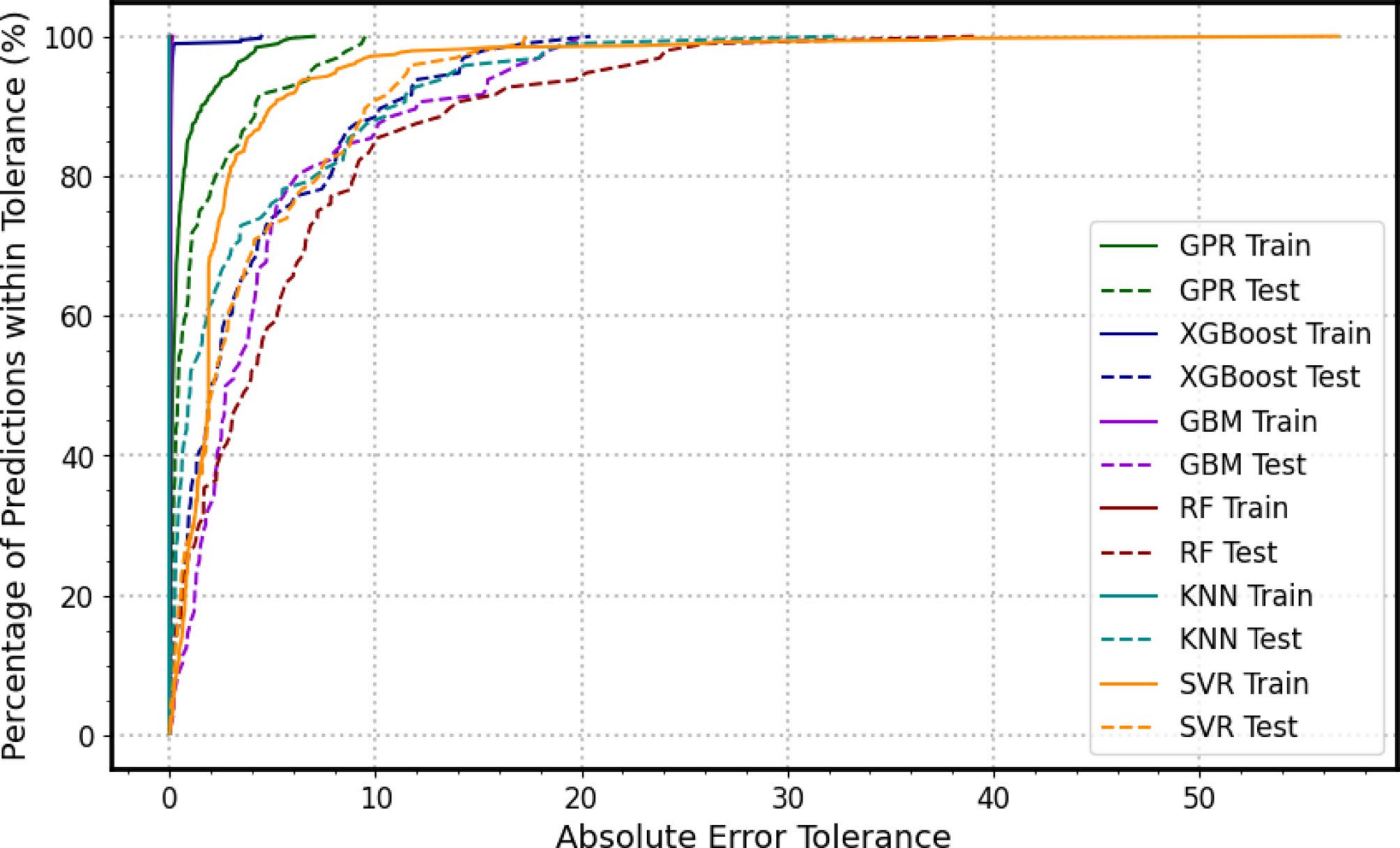
REC curves illustrate the performance of the adopted models during the training and testing.

A comprehensive evaluation of the six machine learning models, based on the Regression Error Characteristics (*REC*) curves presented in Fig. 9, reveals a distinct hierarchy of predictive performance in estimating the load settlement behaviors. The analysis identifies Gaussian Process Regression (*GPR*) as the most effective and reliable algorithm, while the remaining models exhibit varying trade-offs between predictive accuracy and generalization capability. The *GPR* model demonstrates exceptional predictive performance and robust generalization, as evidenced by the close alignment between its training and testing curves. This tight coupling indicates that the model has successfully captured the true underlying relationships in the data without overfitting. Furthermore, the *GPR* curve rises steeply, signifying that a high proportion of its predictions fall within a very small error margin. Notably, at a low absolute error tolerance of just 2%, GPR correctly predicts approximately 80% of the targets in both training and testing phases. and require10% absolute error tolerance to capture all predictions. This combination of high precision and strong generalization establishes *GPR* as the most accurate and trustworthy model for this regression task.

In contrast, the *XGBoost*, Gradient Boosting Machine (*GBM*), Random Forest (*RF*), and K-Nearest Neighbors (*KNN*) models display strong learning capacity during training but also exhibit significant overfitting. Their training curves rise almost vertically, suggesting near-perfect accuracy on the training data, yet this performance deteriorates when evaluated on the test set. Each model requires a substantially higher error tolerance to capture all predictions, highlighting the disparity between training and testing behavior: approximately 20% absolute error tolerance for *XGBoost* and *GBM*, 30% for *KNN*, and 40% for RF. This widening gap confirms that while these algorithms are highly capable of learning complex patterns, they struggle to generalize effectively to unseen data, limiting their practical reliability for this specific prediction task.

Finally, the Support Vector Regression (*SVR*) model emerges as the least effective performer. Its REC curve remains shallow and low across all error tolerances, indicating that only a small percentage of predictions achieve moderate accuracy. Although the close proximity between its training and testing curves suggests consistent generalization and the absence of overfitting, this alignment merely reflects the model’s consistent inability to capture the true data patterns. Consequently, despite its theoretical advantages in handling nonlinear relationships, *SVR* produces uniformly inaccurate predictions and is deemed unsuitable for deployment in this application.

Overall, the *REC*-based comparison confirms that *GPR* offers the best trade-off between accuracy and generalization, followed by *XGBoost* and *GBM*, while *RF* provides stable but less precise predictions, and *KNN* and *SVR* lag significantly behind in overall predictive performance.

### Score analysis

To objectively establish the final hierarchy of the six evaluated machine learning models, I implemented a comprehensive Rank Sum scoring methodology. Rather than relying on a single metric, which can provide a skewed perspective of model performance depending on the data distribution, this scoring system aggregates predictive accuracy and error distributions across all eight computed statistical metrics (*R*^2^, *MAPE*, *MAE*, *RMSE*, absolute *MBE*, *A*20-Index, Scatter Index, and Agreement Index). For each metric, the algorithms were ranked from 1 to 6. For accuracy-driven metrics (*R*^2^, *A*20, and *d*), the highest numerical value received the top rank; conversely, for error-driven metrics (*MAPE*, *MAE*, *RMSE*, *SI*, and absolute *MBE*), the lowest value received the top rank. These discrete ranks were subsequently converted into an aggregated point system. Crucially, to explicitly quantify the degree of overfitting and evaluate true generalization capability, this ranking process was performed independently on both the training and testing datasets. The final Comprehensive Score for each algorithm was computed by summing its accumulated points from both phases. This dual-phase evaluation inherently penalizes models that suffer from severe overfitting, such as certain tree-based algorithms that achieved near-perfect training scores but degraded significantly on unseen data, because their testing phase points sharply decline.

**Fig. 10:**
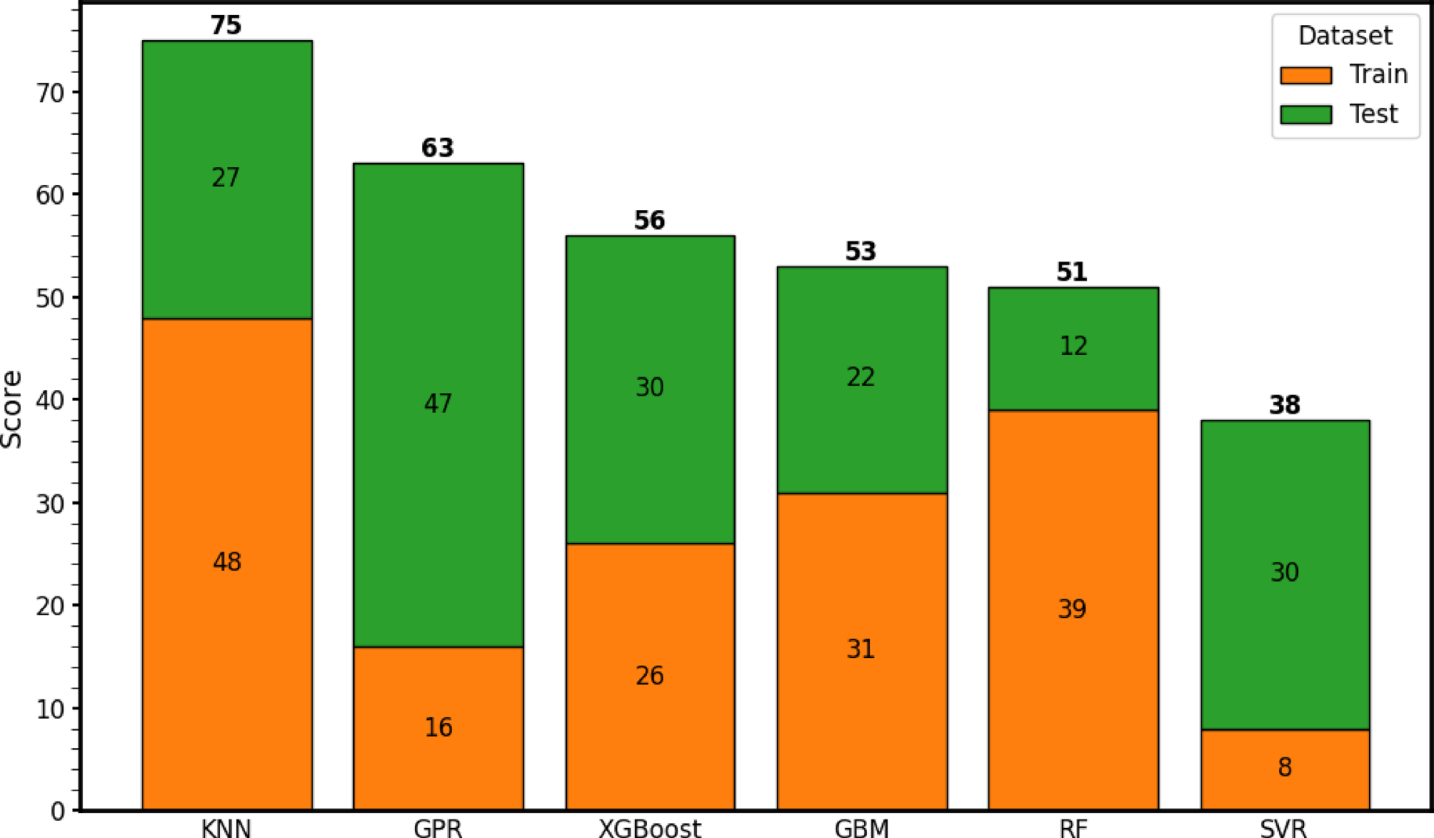
Score analysis indicates the performance of *ML* models.

The score analysis provides a comprehensive comparison and ranking of six machine learning algorithms, *KNN*, *GPR*, *XGBoost*, *GBM*, *RF*, and *SVR*, based on their performance across both training and testing datasets, as illustrated in Fig. 10. The bar chart clearly depicts the distribution of training and testing scores, enabling a direct assessment of each model’s learning behavior and generalization capability.

Among the evaluated models, the K-Nearest Neighbors (*KNN*) algorithm achieved the highest overall score (75), largely due to its strong training performance (48). However, its testing score (27) declined significantly, suggesting that *KNN* may have overfitted the training data, capturing noise rather than the underlying pattern. In contrast, the Gaussian Process Regressor (*GPR*) demonstrated the most balanced and generalizable performance, recording a relatively low training score (16) but an exceptionally high testing score (47), reflecting strong adaptability to unseen data and excellent model robustness.

The *XGBoost* model exhibited consistent results across both datasets, with training and testing scores of 26 and 30, respectively. This indicates a healthy balance between bias and variance, implying that the model successfully generalized without overfitting. The Gradient Boosting Machine (*GBM*) yielded moderate outcomes (31 for training and 22 for testing), representing average stability and reliable, though not outstanding, predictive capability.

On the other hand, the Random Forest (*RF*) model displayed a clear case of overfitting, achieving a high training score (39) but a markedly low testing score (12), indicating poor generalization despite its strong performance on the training data. The Support Vector Regression (*SVR*) model showed the opposite tendency, with a low training score (8) but a higher testing score (30), suggesting potential underfitting, that is, the model might not have captured the full complexity of the training data but still performed reasonably well on new inputs.

Overall, the comparative analysis indicates that *GPR* and *XGBoost* provided the most optimal balance between training accuracy and generalization, making them the most reliable models for predicting load–settlement behavior. In contrast, *KNN* and *RF* suffered from overfitting, while *SVR* tended toward underfitting, highlighting the importance of appropriate hyperparameter tuning and model selection in achieving robust predictive performance.

### Feature importance analysis

**Fig. 11:**
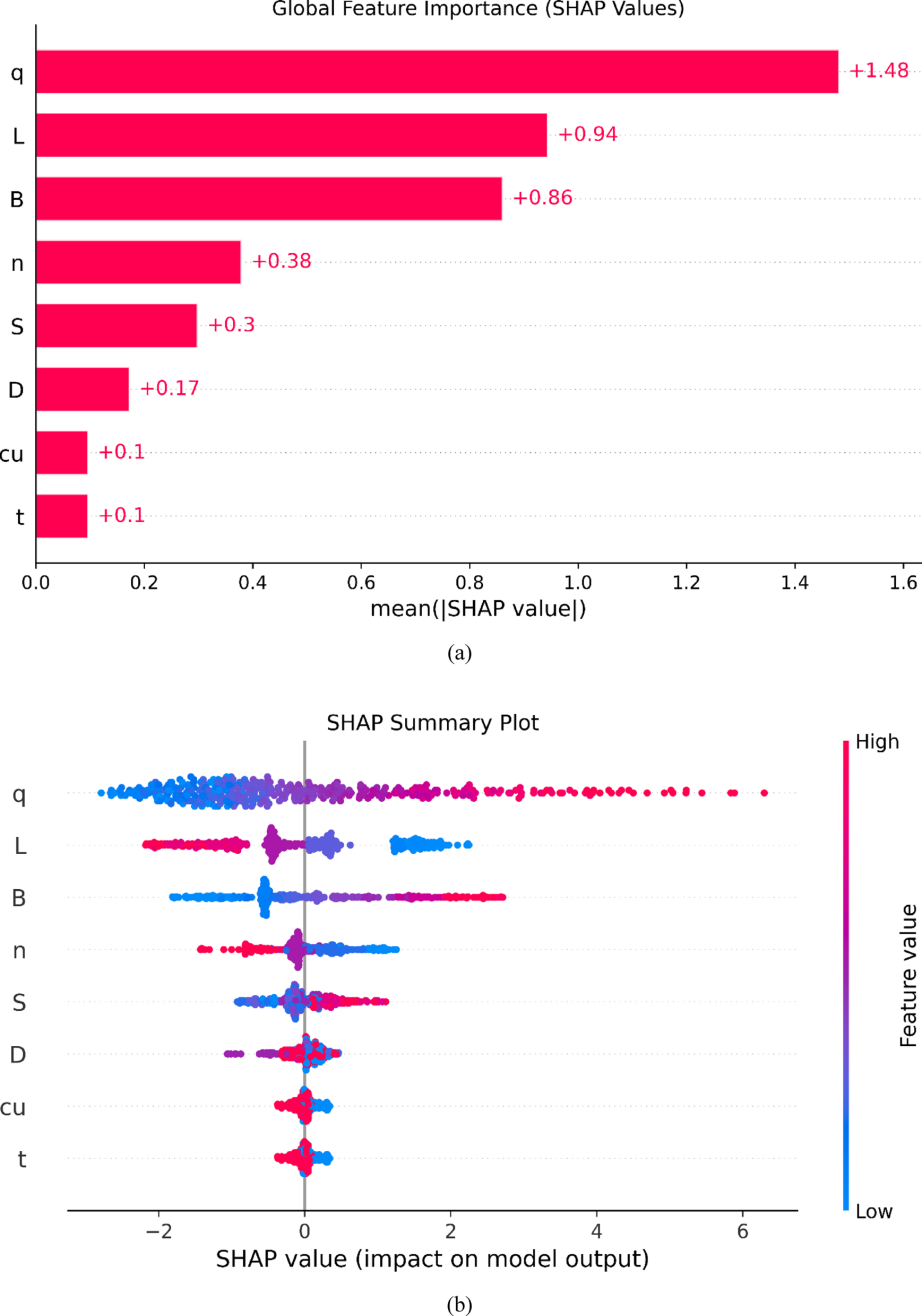

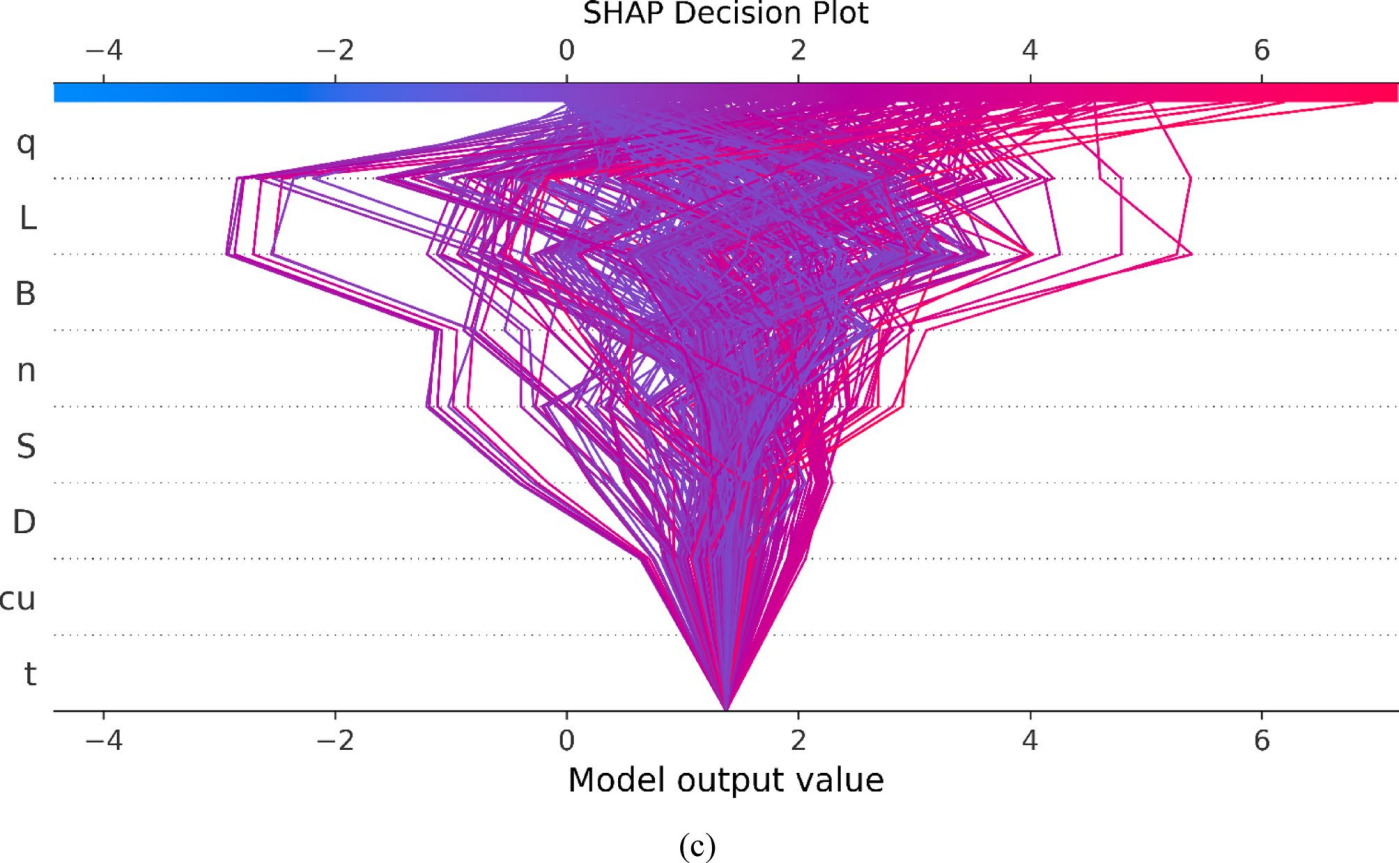
*SHAP* decision plots for micro-piled raft database.

The SHAP feature importance analysis, illustrated in Fig. 11(a), provides a detailed examination of how the Gaussian Process Regression (*GPR*) model interprets and weighs different input parameters when predicting the settlement (Se) of micro-piled raft foundations. Using the Shapley Additive Explanations (*SHAP*) framework, each feature’s contribution to individual predictions is quantified and then aggregated to produce the mean absolute *SHAP* value across the entire dataset. The magnitude of this value directly reflects the global importance of each input variable, where larger *SHAP* values indicate a stronger overall influence on the model’s predictions.

The global feature importance ranking in Fig. 11(a) reveals a clear and physically consistent hierarchy. Applied stress (*q*) is identified as the most influential factor, with a mean absolute *SHAP* value of + 1.48, confirming its dominant role in governing settlement behavior. This strong influence reflects the fundamental relationship between applied load and deformation, as recognized in geotechnical mechanics, while also validating that the model has correctly learned this dependency from data.

Following this, the pile length (*L*) exhibits a mean absolute *SHAP* value of + 0.94, underscoring its critical role in controlling axial stiffness and soil–pile interaction. The raft width (*B*) ranks third (+ 0.86), consistent with its function in distributing stress and influencing the load-sharing mechanism between the raft and micro-piles. Together, these three parameters form the primary control group over predicted settlement, which the *GPR* model captures with high fidelity.

Parameters such as the number of piles (*n*), pile spacing (*S*), and pile diameter (*D*) show progressively lower average importance, with *SHAP* values of + 0.38, + 0.3, and + 0.17, respectively. These variables still influence the overall foundation stiffness and group interaction effects but contribute less significantly to global settlement variation. The raft thickness (*t*) and undrained shear strength (*c*_*u*_) display the smallest *SHAP* values (+ 0.1), suggesting their effects are secondary within the range of configurations analyzed. Although both parameters influence structural rigidity and bearing capacity, their impact is less pronounced relative to geometric and loading factors.

The *SHAP* summary plot (Fig. 11(b)) extends this interpretation by illustrating the direction and non-linearity of each feature’s influence. High applied stress values (red points) are associated with strongly positive *SHAP* values, indicating that incremental increases in *q* cause disproportionately large rises in predicted settlement, a clear non-linear response. For features such as pile length (*L*) and raft width (*B*), the *SHAP* values change sign depending on other parameters, revealing interaction effects, for instance, short piles exhibit different settlement behavior depending on applied stress or soil strength. These cross-dependencies reflect complex interactions captured by the *GPR* model that go beyond linear geotechnical assumptions.

The *SHAP* decision plot (Fig. 11(c)) visualizes the cumulative reasoning behind individual predictions, showing how each feature incrementally shifts the model’s output from the base value toward the final predicted settlement. The color-coded traces reveal how combinations of input values either amplify or mitigate predicted settlement, demonstrating that the *GPR* model not only aligns with established geotechnical theory but also captures subtle, data-driven variations and interactions.

Finally, the thickness of the raft (*t*) and the undrained shear strength of the clay (*c*_*u*_) exhibit the lowest mean absolute *SHAP* values, both at + 0.1. This result must be interpreted with extreme caution and is considered a computational artifact rather than a reflection of true geotechnical behavior. Due to the perfect multicollinearity (Pearson’s *r* = 1.0) observed between these two variables, the *GPR* model and the subsequent *SHAP* analysis cannot deconvolve their individual effects. The model cannot assign importance to one variable over the other when they provide identical information. Therefore, the low *SHAP* values are a direct consequence of this informational redundancy and do not diminish the fundamental geotechnical principle that soil strength is a critical factor in foundation performance.

Finally, the thickness of the raft (*t*) and the undrained shear strength of the clay (*c*_*u*_) exhibit the lowest mean absolute *SHAP* values, both at + 0.1. As anticipated from the correlation analysis, the perfect multicollinearity between these two variables manifests as a mathematical artifact within the *SHAP* interpretability framework. Because *t* and *c*_*u*_ supply statistically identical information to the model, the algorithm cannot mathematically deconvolve their independent contributions, resulting in artificially diluted importance scores for both. I strongly advise readers that these low *SHAP* values are strictly a computational artifact of the dataset’s informational redundancy, not a reflection of true physical behavior. In reality, both structural rigidity and soil strength remain fundamental, critical drivers of foundation settlement, and their retention was necessary to preserve the physical completeness of the model’s input space.

The relative importance and directional influence of the key predictors identified by the machine learning models are fully consistent with established geotechnical understanding of micro-piled raft behavior. Applied stress governs settlement magnitude through nonlinear soil deformation; pile length controls stiffness and load transfer depth; and raft geometry regulates stress distribution and pile–raft interaction. This consistency confirms that the developed *ML* models learn physically meaningful relationships rather than purely statistical correlations.

### Probabilistic analysis

The probabilistic analysis in this study employs Monte Carlo simulations (*MCS*) to assess the impact of uncertainties in the geometric and soil parameters on the predicted settlement of micro-piled raft foundations. The input parameters considered include micro-pile diameter (*D*), length (*L*), number (*n*), spacing (*S*), raft width (*B*), raft thickness (*t*), and the undrained shear strength of the clay (*c*_*u*_),. Each parameter is assigned a probability distribution reflecting controlled experimental variability, and random samples are generated to propagate uncertainty through the pre-trained GPR model.

It is important to note that the applied load (*q*) is treated as a static vertical load and considered deterministic. The *MCS* do not introduce variability in the loading; rather, they evaluate the probabilistic response of the foundation for each fixed load level. This approach allows the study to quantify the uncertainty in predicted settlements due to inherent variability in the foundation and soil characteristics while maintaining controlled, realistic loading conditions.

The choice of probability distributions and associated standard deviations was carefully designed to reflect the controlled experimental uncertainties within the dataset, which includes nine small-scale laboratory tests and thirty-two large-scale field tests. Three distribution types: Normal, Lognormal, and Gamma, were used to test the model’s sensitivity to different forms of uncertainty. The standard deviations for each input parameter were defined as fixed percentages of their mean values: 0.5% for pile diameter (*D*), raft thickness (*t*), and undrained shear strength (*c*_*u*_), and 1.0% for pile length (*L*), pile spacing (*S*), and raft width (*B*). For example, a micro-pile with a diameter of 50 mm was sampled with a standard deviation of 0.25 mm, while a raft width of 560 mm was assigned a deviation of 5.6 mm. These narrow margins were intentionally selected to simulate measurement precision and fabrication tolerances typical of experimental testing, rather than natural geotechnical variability. The resulting coefficient of variation (COV ≈ 0.5–1%) isolates the influence of controlled experimental randomness while maintaining consistency with the data used to train the *GPR* model. The Normal distribution captures symmetric variability, the Lognormal accounts for positive-only multiplicative variation, and the Gamma provides an alternative for skewed strength-related parameters. This configuration ensures that the uncertainty propagation analysis remains focused on quantifying model robustness under realistic experimental conditions, while large-scale field variability is reserved for future probabilistic calibration and validation.

For each predefined uniform load value, the Monte Carlo simulation iterates 1,000 times, introducing random variations to the input parameters according to the selected probability distributions. These distributions are centered on the base configuration means, with small standard deviations reflecting realistic deviations from nominal design due to construction tolerances or minor soil heterogeneity. Specifically, *D*, *L*, *S*, *B*, *t*, and *c*_*u*_ are treated as continuous random variables, while n is held constant. In each iteration, a unique input vector, comprising the fixed applied stress (*q*) and randomly sampled input parameters, is fed into the pre-trained Gaussian Process Regression (*GPR*) model to predict the corresponding settlement (*S*_*e*_). The predicted settlements and their input combinations are stored for statistical post-processing.

Following completing the *MCS* for each load, these stored predictions are used to derive key statistical insights, including mean predicted settlements, confidence intervals, and the distribution of predicted settlements. This probabilistic approach provides a more realistic assessment of foundation performance, offering not only a single deterministic prediction but a range of likely settlement outcomes and associated probabilities. The final output is a probabilistic load-settlement curve, providing a comprehensive understanding of foundation behavior under uncertain operating conditions and enabling direct comparison with experimental results.

**Fig. 12:**
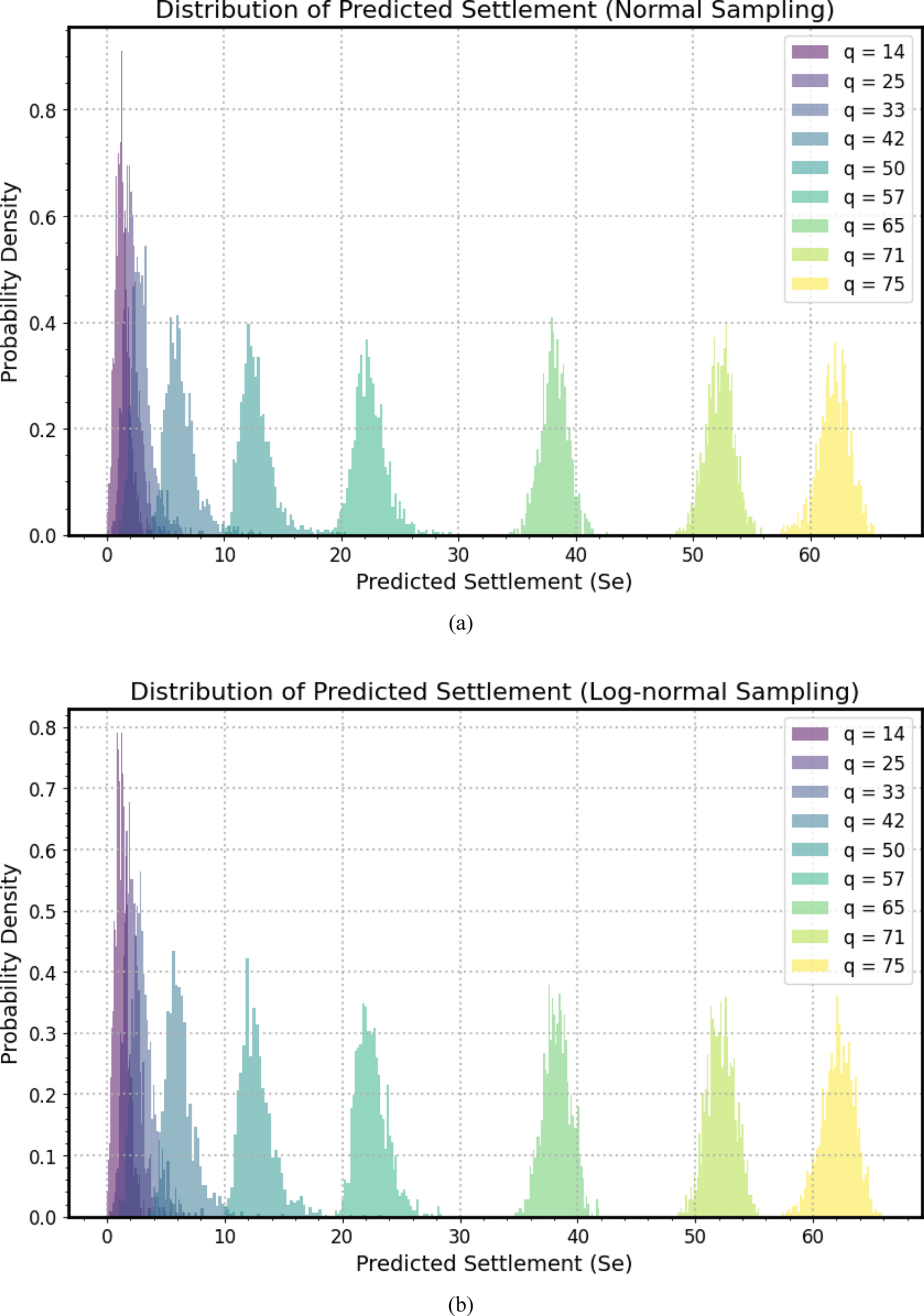

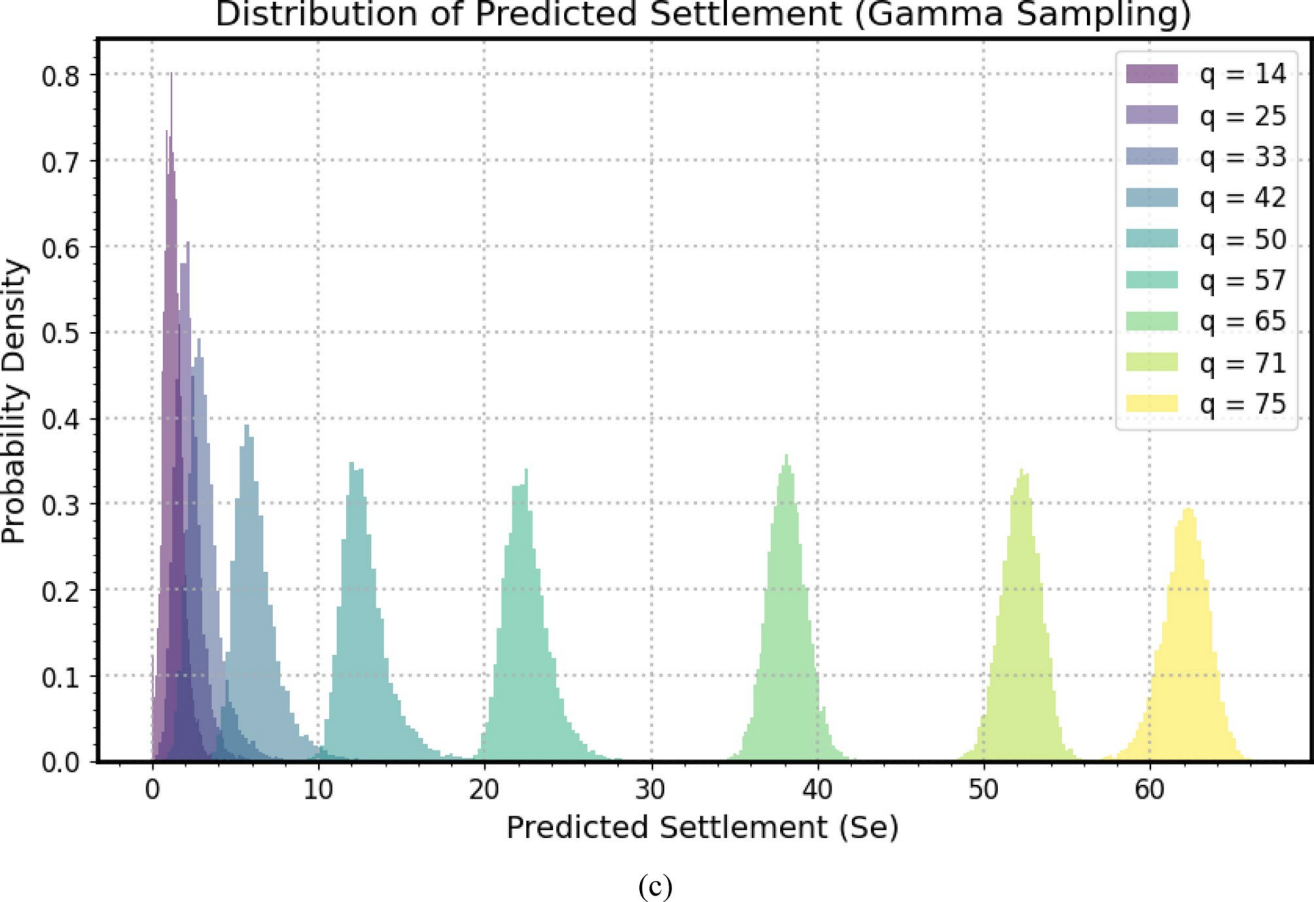
A comprehensive sensitivity analysis: (**a**) Normal distributions, (**b**) Lognormal distributions, (**c**) Gamma distributions.

Figure 12(a–c) present a comprehensive sensitivity analysis, illustrating the probabilistic distribution of predicted settlement (*S*_*e*_) for a micro-piled raft foundation across a range of applied stress (*q*), selected from available experimental tests. Figure 12(a) shows Normal distributions, Fig. 12(b) presents Lognormal distributions, and Fig. 12(c) depicts Gamma distributions. Each colored probability density histogram corresponds to specific applied stress, ranging from *q* = 14 to *q* = 75, allowing for a detailed examination of how predicted settlement varies with load magnitude and the uncertainties propagated through the *GPR* model during the Monte Carlo simulation. The horizontal axis delineates predicted settlement values, providing a quantitative scale for assessing the foundation’s deformation, while the vertical axis quantifies probability density, indicating the likelihood of observing specific settlement values for each loading scenario. A clear trend emerges as the applied stress increases and the distribution of predicted settlement shifts progressively toward higher values. This aligns fundamental geotechnical principles, were increased load leads to greater deformation and settlement.

The shape and spread of each histogram offer insights into uncertainty associated with settlement predictions. Narrower distributions indicate higher confidence and lower variability, while wider ones suggest greater uncertainty. Notably, there is a subtle trend of increasing spread in predicted settlement distributions as applied stress intensifies, possibly reflecting the nonlinear behavior of soil-structure interaction or the model’s sensitivity to input variability at higher loads. The peak of each histogram represents the most probable settlement value for the corresponding applied stress, indicating the mode of the distribution. The progression of these peaks across different load scenarios provides a probabilistic load-settlement curve. The spacing of modal values can indicate linearity or nonlinearity in the foundation response equal spacing suggests linearity, while unequal spacing indicates nonlinear behavior.

This probabilistic sensitivity analysis informs risk assessment in designing micro-piled raft foundations. By examining the area under each probability density curve beyond critical settlement thresholds, engineers can quantify the likelihood of exceeding allowable settlement limits. This insight is crucial for ensuring the stability of the structure supported by the foundation, enabling a design that accounts for inherent uncertainties and provides robust performance against excessive deformation. The visual representation of these distributions is a powerful tool for communicating expected performance and associated risks to stakeholders in geotechnical engineering projects.

**Fig. 13:**
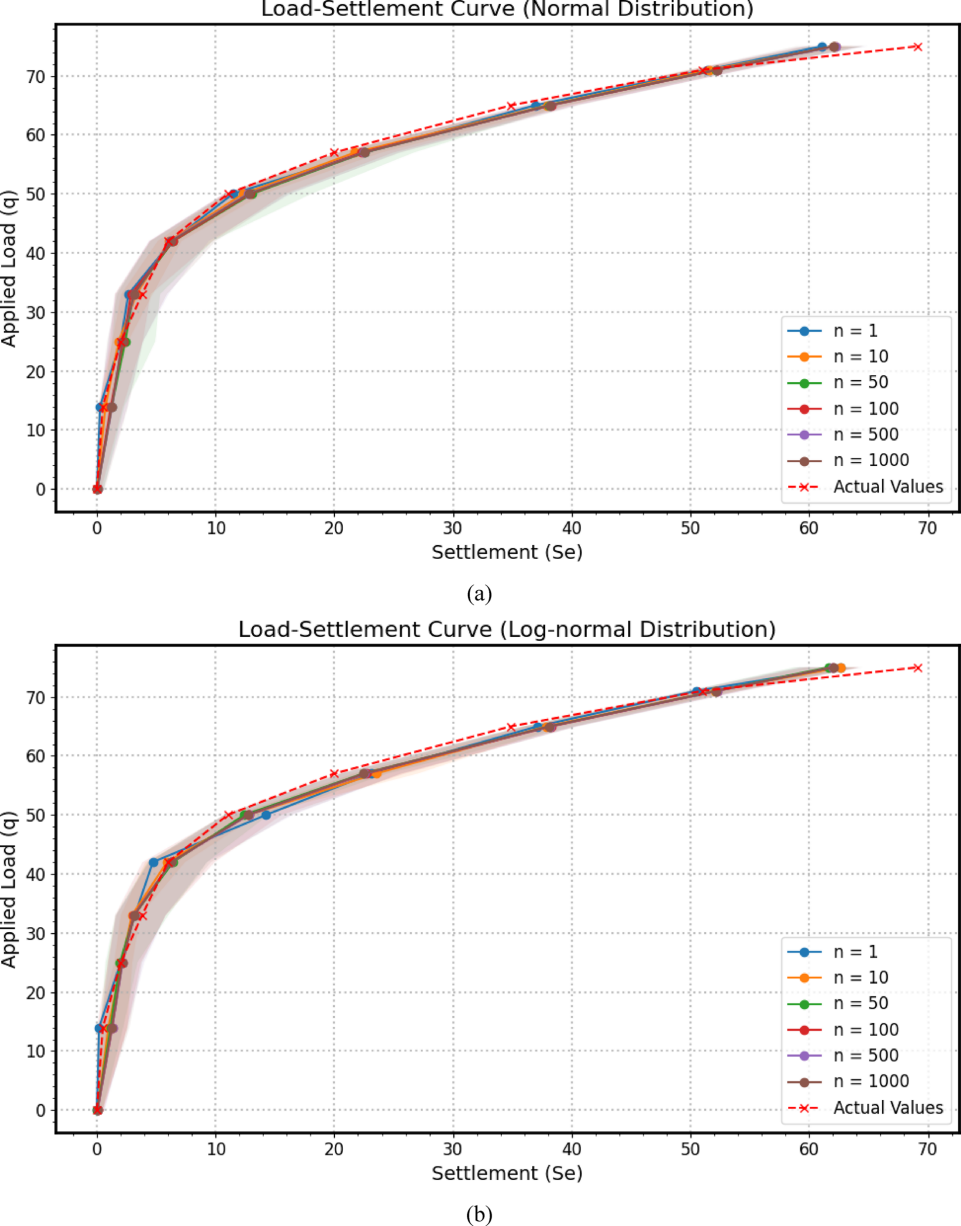

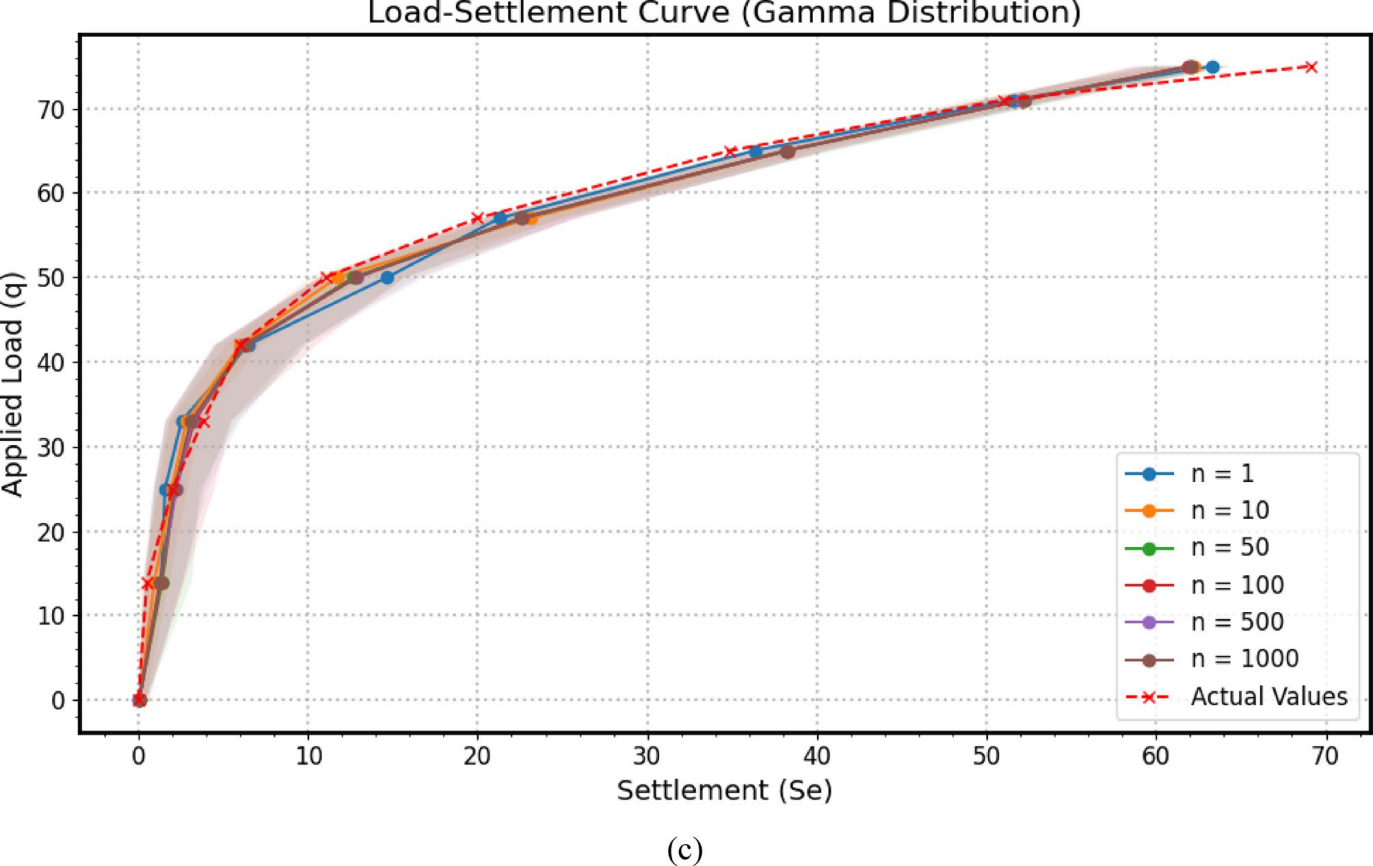
The predicted load-settlement curve using Monte Carlo simulation: (**a**) Normal distributions, (**b**) Lognormal distributions, (**c**) Gamma distributions.

Across the three distinct subfigures comprising Fig. 13 – specifically Fig. 13(a) illustrating results from a Monte Carlo simulation employing Normal distributions for input parameters, Fig. 13(b) showcasing outcomes based on Lognormal distributions, and Fig. 13(c) presenting findings derived from Gamma distributions. For each predefined uniform load value, the Monte Carlo sensitivity analysis was performed with 1, 10, 50, 100, 500, and 1000 iterations. It was observed that using more than one iteration produced essentially the same results, reflecting the low variability in the input parameters. A fundamentally similar analytical framework is consistently applied to evaluate the *GPR* model’s predictive capabilities regarding the load-settlement behavior of a micro-piled raft foundation. Each subfigure juxtaposes the model’s mean predicted settlement against actual experimentally measured settlement values while incorporating a 95% confidence interval around the predicted response to quantify the inherent uncertainty associated with the model’s estimations. The horizontal axis represents settlement (*S*_*e*_), measuring the foundation’s deformation under load, while the vertical axis quantifies the applied stress (*q*), establishing the independent variable that drives the settlement response. The overarching trends of the predicted load-settlement curves and their general alignment with the actual data exhibit a degree of visual resemblance across the three distributional scenarios in Fig. 13(a–c).

The blue solid line, adorned with circular markers and labeled “Mean Predicted,” traces the *GPR* model’s average predicted settlement across the range of applied stress analyzed using Monte Carlo simulation. This line represents the model’s best estimate of the foundation’s load-settlement behavior, effectively acting as a predictive trend line. The proximity of this predicted curve to the red dashed line, marked with crosses and representing the “Actual Values,” offers a direct visual assessment of the model’s accuracy. Close alignment between these two curves signifies a high degree of predictive capability, indicating that the GPR model has successfully learned the underlying relationship between the applied stress and the resulting settlement. Conversely, noticeable deviations highlight potential areas where the model’s predictions diverge from the observed behavior, suggesting possible limitations in its ability to fully capture the intricate soil-structure interaction or the influence of unmodeled factors.

Encircling the mean predicted settlement curve is a light blue shaded region, denoting the “95% Confidence Interval.” This band visually encapsulates the uncertainty associated with the model’s predictions, derived from the probabilistic framework of the Monte Carlo simulation that likely perturbed the input parameters based on their inherent variability. A 95% confidence interval implies that for any given applied stress, there is a 95% probability that the true or actual settlement would fall within this shaded region, assuming the model’s uncertainty quantification is well-calibrated. A narrow confidence interval suggests a high degree of certainty in the model’s predictions, implying that repeated predictions under similar conditions would likely yield settlement values within a tight range. Conversely, a wider confidence interval indicates greater uncertainty, suggesting a broader spectrum of potential actual settlement values for a given load. The direct comparison of the actual settlement values against the predicted curve and its associated confidence interval is paramount for evaluating the model’s predictive robustness. If the red dashed line, representing the actual behavior, consistently resides within the bounds of the light blue shaded confidence interval, it provides strong evidence that the model’s predictions are not only accurate on average but also that its estimation of uncertainty is reasonable. This suggests that the model is capable of capturing the inherent variability in the system and providing a statistically consistent range of expected outcomes. However, if the actual values frequently fall outside the 95% confidence interval, it might indicate that the model is underestimating the true uncertainty, potentially due to limitations in the training data, the model’s complexity, or the assumptions made during the uncertainty propagation process.

**Fig. 14:**
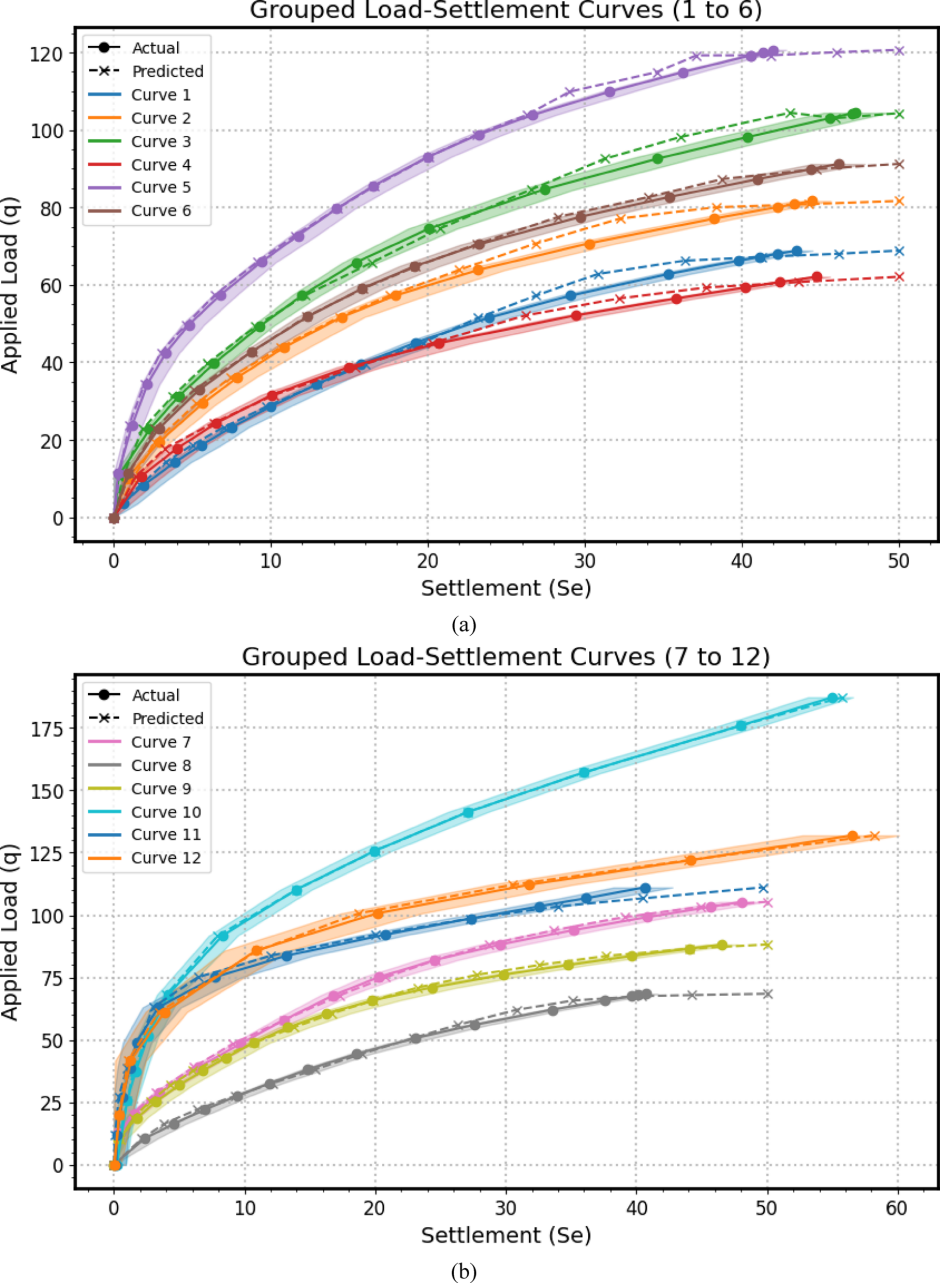

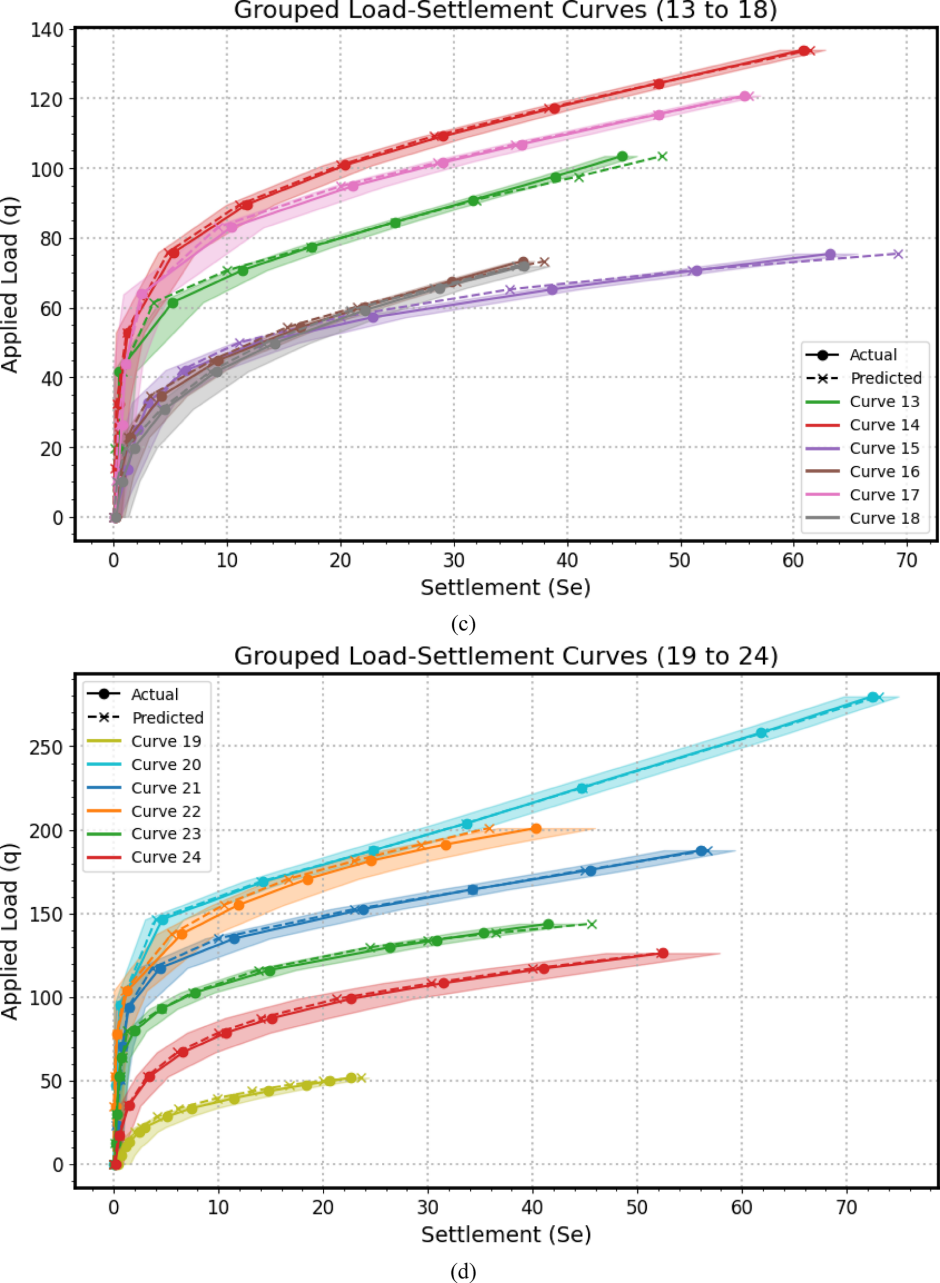

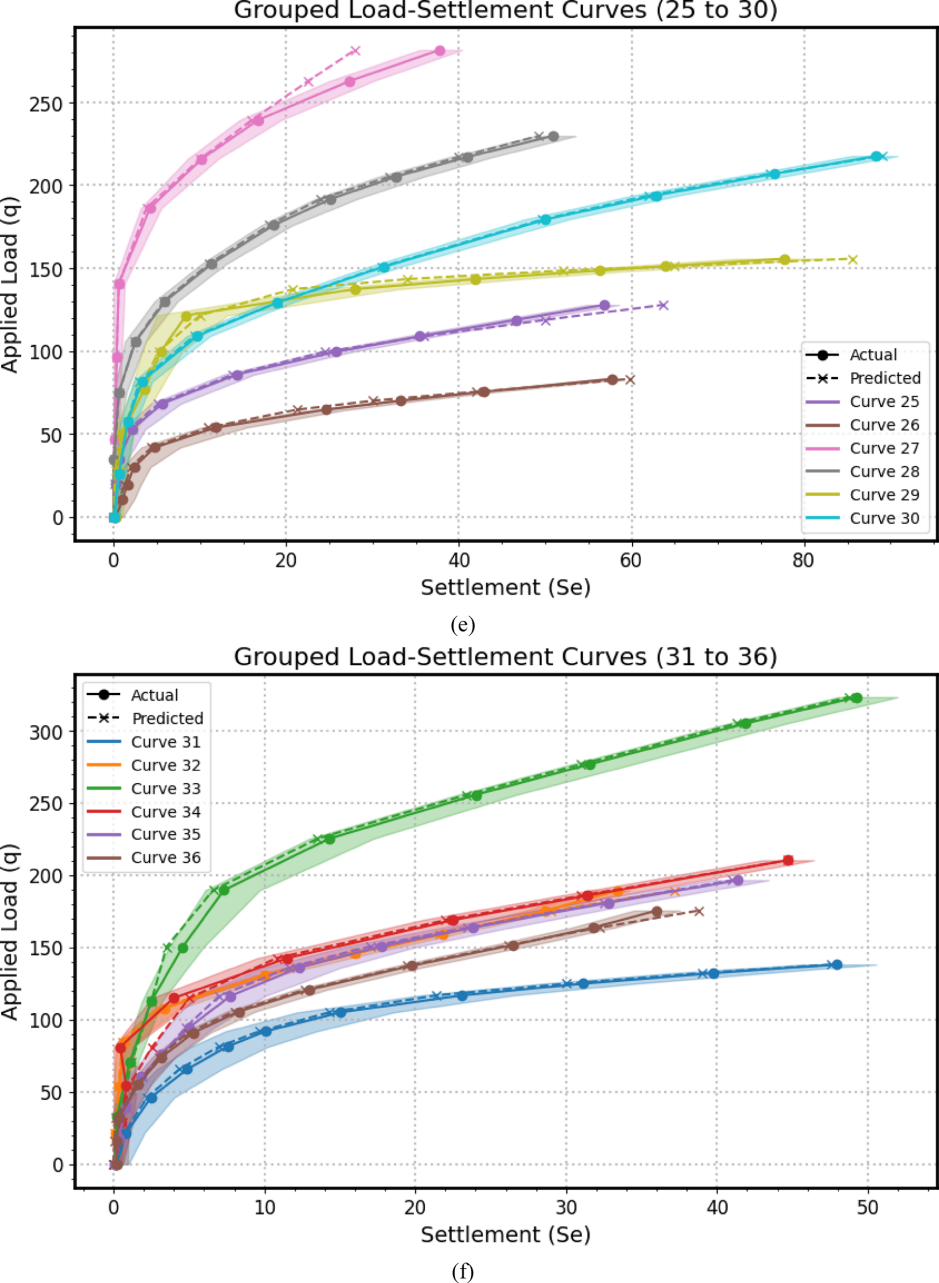

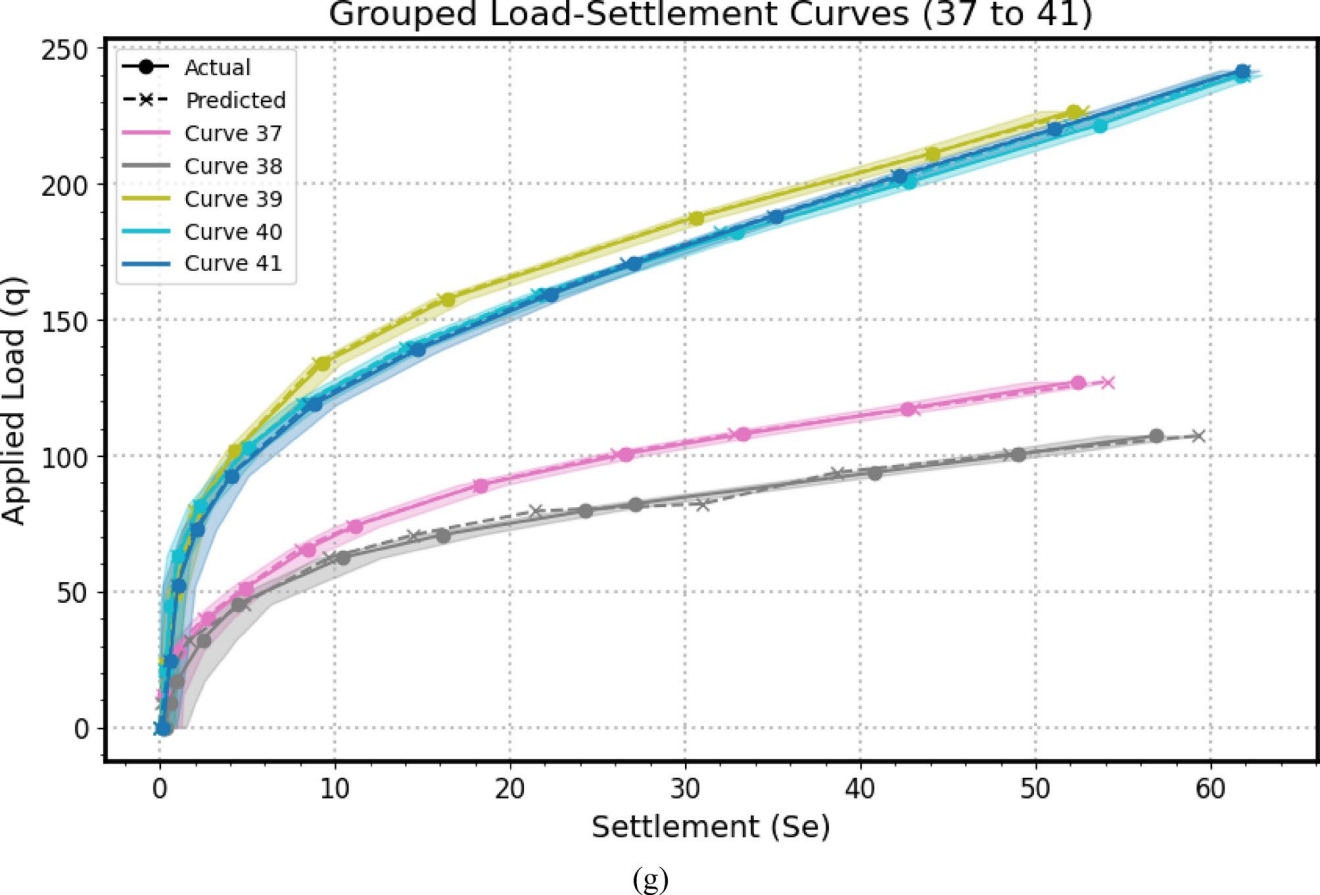
The predicted Load-Settlement Curve using Monte Carlo simulation and Normal distributions.

Encompassing seven distinct graphical representations labeled as Fig. 14(a–g), this comprehensive visualization strategy serves to systematically evaluates the predictive prowess and associate uncertainties of the *GPR* model across a substantial dataset of 41 experimental load-settlement curves for micro-piled raft foundations. Each individual subfigure within this series, similar in structure to the example provided previously, presents a grouped analysis of predicted versus actual load-settlement behavior for a subset of these curves. Specifically, each subfigure juxtaposes solid lines with markers, representing the GPR model’s predicted settlement response under varying applied stress levels, derived from *MCS* to account for input parameter variability using Normal distributions, against dashed lines with distinct markers, illustrating the corresponding experimentally measured settlement values for each curve within that group. Furthermore, a shaded region enveloping each predicted curve visually delineates the 95% confidence interval, effectively quantifying the inherent uncertainty associated with the model’s estimations for that specific curve. By partitioning the total of 41 curves into seven manageable groups across these subfigures (Fig. 14(a): Curves from 1 to 6, Fig. 14(b): Curves from 7 to 12, Fig. 14(c): Curves from 13 to 18, Fig. 14(d): Curves from 19 to 24, Fig. 14(e): Curves from 25 to 30, Fig. 14(f): Curves from 31 to 36, Fig. 14(g): Curves from 37 to 41), this approach facilitates a detailed visual comparison of the model’s performance in terms of both predictive accuracy (the proximity of the solid predicted line to the dashed actual line) and the consistency of its uncertainty quantification (the width of the shaded confidence interval) across a diverse range of experimental conditions or foundation configurations represented by the different curve numbers. This systematic presentation allows for the identification of potential variations in the model’s generalization capabilities, the consistency of its uncertainty estimates, and the detection of any systematic biases or limitations across the entirety of the experimental dataset.

The alignment between the solid predicted lines and the dashed actual lines reveals the model’s accuracy in estimating settlement under specific applied stresses, with close overlap indicating strong predictive performance and divergence suggesting potential under- or over-estimation. Furthermore, the varying width of the shaded 95% confidence intervals quantifies the model’s uncertainty, where wider bands imply lower confidence potentially due to limited data or experimental variability, and narrower bands suggest higher prediction certainty; crucially, the model’s ability to replicate the characteristic nonlinear stress-settlement relationship is evident in both predicted and actual curves, impacting its overall predictive robustness. Finally, examining each curve individually highlights the consistency of the model’s performance and uncertainty levels across diverse experimental conditions.

## Conclusions

This study comprehensively evaluated the predictive performance of six supervised machine learning (*ML*) algorithms, Gaussian Process Regression (*GPR*), Extreme Gradient Boosting (*XGBoost*), Gradient Boosting Machine (*GBM*), Random Forest (*RF*), K-Nearest Neighbors (*KNN*), and Support Vector Regression (*SVR*), for modeling the load–settlement behavior of micro-piled raft foundations in clayey soils. The models were developed and validated using an extensive dataset of 480 samples derived from small-scale laboratory and large-scale field tests. Based on the multi-metric evaluation, explainability analysis, and uncertainty quantification, the following conclusions are drawn:

- The findings highlight the importance of evaluating model generalization using an independent testing dataset. While all models achieved high accuracy on training data, *GPR* demonstrated the most balanced and robust performance on unseen data, achieving superior generalization and low prediction error. In contrast, *XGBoost*, *GBM*, *RF*, and *KNN* showed signs of significant overfitting, with large discrepancies between training and testing performance. This indicates a need for advanced regularization techniques, hyperparameter optimization, and possibly larger or more diverse training datasets to improve their generalizability. *SVR*, despite consistent behavior across datasets, produced higher errors overall, suggesting potential underfitting and warranting further tuning of kernel functions and model parameters.
- Regression Error Characteristics (*REC*) Curve Analysis demonstrated that Gaussian Process Regression (*GPR*) outperformed all other models by delivering the highest accuracy with strong generalization, as indicated by the tight alignment of training and testing error curves. In contrast, models like *XGBoost*, *GBM*, *RF*, and *KNN* exhibited significant overfitting, requiring much higher error tolerance to capture predictions during testing, while *SVR* showed consistently poor accuracy despite its stability.
- Score Analysis corroborated these findings, showing that *GPR* achieved a superior balance between training and testing scores, indicative of robust predictive performance and adaptability to unseen data. Models such as *KNN* and *RF* exhibited high training but low testing scores, reinforcing the presence of overfitting, whereas *SVR’s* low training but relatively better testing scores suggested underfitting.
- *SHAP*-based interpretability analysis revealed that applied stress (*q*), pile length (*L*), and raft width (*B*) were the most influential variables affecting settlement predictions, in alignment with geotechnical principles. The number of piles (*n*) exerted moderate influence, while pile spacing (*S*) and pile diameter (*D*) were of lesser importance. Raft thickness (*t*) and undrained shear strength (*c*_*u*_) showed the lowest mean *SHAP* values, possibly due to multicollinearity rather than true lack of influence. These insights are crucial for understanding the key design drivers and guiding targeted parameter studies in future geotechnical applications.
- The predicted load–settlement responses accurately reflected the nonlinear behavior inherent to soil–structure interaction. The *GPR* model captured the initial high-stiffness region followed by a gradual reduction in stiffness at higher loads, consistent with observed experimental trends. This capability confirms the model’s practical utility for representing the full spectrum of foundation behavior under varying load intensities. The visual and statistical alignment between predicted and actual curves provides engineers with confidence in using *GPR*-based tools for settlement prediction and design evaluation.
- Probabilistic analysis using Monte Carlo simulations, incorporating realistic variability in key geometric and geotechnical parameters, was performed to evaluate the robustness of the GPR model. The strong agreement between the mean predicted load–settlement curves and experimental data, along with narrow 95% confidence intervals, highlights the model’s high predictive reliability. This probabilistic approach enhances model evaluation by quantifying the range of possible outcomes, thereby supporting more informed and risk-aware decision-making in foundation design.
- Overall, while all models demonstrated some predictive capability, the combined insights from statistical evaluation methods and visual diagnostics, such as scatter plots for predicted-versus-actual plots, along with regression error characteristics (*REC*) curves, score analysis, and hyperparameter tuning, clearly position *GPR* as the most promising algorithm for modeling micro-piled raft foundation settlement. Future research should focus on refining model parameters, expanding dataset diversity, and exploring advanced tuning techniques to further enhance the robustness and reliability of all evaluated models.

The findings should be interpreted within the experimental and modeling boundaries defined in this study, with full-scale validation identified as a key direction for future work.

## Limitations and future work

While this study successfully identifies the Gaussian Process Regression (*GPR*) model as a robust tool for predicting the load-settlement behavior of micro-piled rafts, it is important to acknowledge several limitations that provide clear directions for future research.

First, the dataset used for training and validation is primarily derived from experiments conducted on small-scale physical models. This reliance on laboratory-scale data introduces potential scale effects, as the stress-level dependency of soil stiffness and particle size effects are not fully replicated in miniature models. Therefore, while the developed *GPR* model demonstrates excellent predictive capability on the compiled dataset, its direct application to the design of full-scale foundations should be approached with caution. A critical next step is to validate the model’s performance against a comprehensive set of full-scale field data to confirm its applicability and accuracy in real-world engineering scenarios.

Second, a significant statistical limitation of the current dataset is the perfect multicollinearity (Pearson’s *r* = 1.0) observed between raft thickness (*t*) and undrained shear strength (*c*_*u*_). Although both parameters represent fundamentally distinct physical properties—structural geometry versus geotechnical capacity—and were deliberately retained to maintain a physically complete definition of the soil-structure system, this informational redundancy makes it mathematically impossible for any model to deconvolve the independent predictive contributions of these two variables. Consequently, the low feature importance attributed to t and *c*_*u*_ by the *SHAP* analysis is a computational artifact and should not be interpreted as a reflection of true geotechnical behavior. Future work should aim to collect or generate data where these variables are independent to enable a more accurate assessment of their individual influence.

Finally, the scope of this study was focused on a rigorous inter-comparison of various machine learning architectures. To strengthen the practical relevance of these results, a crucial avenue for future research is to benchmark the performance, computational efficiency, and accuracy of the superior GPR model directly against traditional analytical methods (e.g., Terzaghi, Meyerhof) and rigorous three-dimensional Finite Element Method (FEM) modeling. Such a comparative study would provide essential context for the model’s performance and help quantify its advantages in terms of accuracy and computational efficiency. Furthermore, deploying the trained and validated *GPR* model into a user-friendly software pipeline or web application would bridge the gap between theoretical research and practical engineering design, providing a valuable tool for the geotechnical community.

## Supplementary Information

Below is the link to the electronic supplementary material.

Supplementary Material 1

## Data Availability

All data supporting the findings of this study are available from the corresponding author upon reasonable request.

## Abbreviations

A20: A20 index
*AI*: Artificial intelligence
*B*: Raft width
BO: Bayesian optimization
COV: Coefficient of variation
*c*_*u*_: Undrained shear strength
CV: Fold cross-validation
*D*: Micro-piles diameter
*d*: Agreement index
*GBM*: Gradient boosting machine
*GPR*: Gaussian process regressor
*KNN*: K-nearest neighbors regression
*L*: Micro-piles length
*MAE*: Mean absolute error
*MAPE*: Mean absolute percentage error
*MBE*: Mean bias error
*MCS*: Monte Carlo simulations
*ML*: Machine learning
*n*: Micro-piles number
*q*: Applied stress
*r*: Pearson correlation coefficient
*R*^2^: Coefficient of determination
*REC*: Regression error characteristics
*RF*: Random forest regressor
*RMSE*: Root means square error
*S*: Micro-piles spacing
S_e_: Micro-pile raft settlement
*SHAP*: SHapley additive exPlanations
*SI*: Scatter index
*SVR*: Support vector regression
*t*: Raft thickness
*VIF*: Variance inflation factor
*XGBoost*: Extreme gradient boost machine
