# Supplementary material for: Data-driven prediction of micro-piled raft load–settlement using machine learning and Monte Carlo simulation
Source: Sci Rep. 2026 May 26;16:16180. doi: 10.1038/s41598-026-54119-6 (PMC13201751; doi:10.1038/s41598-026-54119-6)
Supplement: Supplementary file 1 — Supplementary Material 1 [file 41598_2026_54119_MOESM1_ESM.docx]

Table A1 Database for prediction of the Load-settlement curve of micropiled raft.

| *D* | *L* | *n* | *S* | *B* | *t* | *cu* | *q* | *Se* | Curve No. | Refrence |
| --- | --- | --- | --- | --- | --- | --- | --- | --- | --- | --- |
| 10 | 150 | 9 | 60 | 200 | 10 | 16 | 3.61 | 0.36 | 1 | [59] |
| 10 | 150 | 9 | 60 | 200 | 10 | 16 | 8.21 | 1.66 |  |  |
| 10 | 150 | 9 | 60 | 200 | 10 | 16 | 14.28 | 3.32 |  |  |
| 10 | 150 | 9 | 60 | 200 | 10 | 16 | 18.71 | 5.00 |  |  |
| 10 | 150 | 9 | 60 | 200 | 10 | 16 | 23.31 | 7.00 |  |  |
| 10 | 150 | 9 | 60 | 200 | 10 | 16 | 28.55 | 9.72 |  |  |
| 10 | 150 | 9 | 60 | 200 | 10 | 16 | 34.29 | 12.96 |  |  |
| 10 | 150 | 9 | 60 | 200 | 10 | 16 | 39.54 | 16.02 |  |  |
| 10 | 150 | 9 | 60 | 200 | 10 | 16 | 45.12 | 19.70 |  |  |
| 10 | 150 | 9 | 60 | 200 | 10 | 16 | 51.51 | 23.22 |  |  |
| 10 | 150 | 9 | 60 | 200 | 10 | 16 | 57.25 | 26.90 |  |  |
| 10 | 150 | 9 | 60 | 200 | 10 | 16 | 62.83 | 30.84 |  |  |
| 10 | 150 | 9 | 60 | 200 | 10 | 16 | 66.26 | 36.36 |  |  |
| 10 | 150 | 9 | 60 | 200 | 10 | 16 | 67.23 | 41.26 |  |  |
| 10 | 150 | 9 | 60 | 200 | 10 | 16 | 68.04 | 46.16 |  |  |
| 10 | 150 | 9 | 60 | 200 | 10 | 16 | 68.85 | 50.00 |  |  |
| 10 | 250 | 9 | 60 | 200 | 10 | 16 | 10.02 | 1.22 | 2 |  |
| 10 | 250 | 9 | 60 | 200 | 10 | 16 | 19.54 | 2.62 |  |  |
| 10 | 250 | 9 | 60 | 200 | 10 | 16 | 29.39 | 5.26 |  |  |
| 10 | 250 | 9 | 60 | 200 | 10 | 16 | 36.12 | 7.44 |  |  |
| 10 | 250 | 9 | 60 | 200 | 10 | 16 | 43.83 | 10.60 |  |  |
| 10 | 250 | 9 | 60 | 200 | 10 | 16 | 51.70 | 14.36 |  |  |
| 10 | 250 | 9 | 60 | 200 | 10 | 16 | 57.28 | 17.60 |  |  |
| 10 | 250 | 9 | 60 | 200 | 10 | 16 | 63.84 | 21.98 |  |  |
| 10 | 250 | 9 | 60 | 200 | 10 | 16 | 70.72 | 26.90 |  |  |
| 10 | 250 | 9 | 60 | 200 | 10 | 16 | 77.11 | 32.24 |  |  |
| 10 | 250 | 9 | 60 | 200 | 10 | 16 | 80.05 | 38.36 |  |  |
| 10 | 250 | 9 | 60 | 200 | 10 | 16 | 80.86 | 44.24 |  |  |
| 10 | 250 | 9 | 60 | 200 | 10 | 16 | 81.66 | 50.00 |  |  |
| 10 | 350 | 9 | 60 | 200 | 10 | 16 | 11.00 | 0.62 | 3 |  |
| 10 | 350 | 9 | 60 | 200 | 10 | 16 | 22.83 | 1.84 |  |  |
| 10 | 350 | 9 | 60 | 200 | 10 | 16 | 31.36 | 3.76 |  |  |
| 10 | 350 | 9 | 60 | 200 | 10 | 16 | 39.73 | 5.96 |  |  |
| 10 | 350 | 9 | 60 | 200 | 10 | 16 | 49.25 | 9.02 |  |  |
| 10 | 350 | 9 | 60 | 200 | 10 | 16 | 57.29 | 12.18 |  |  |
| 10 | 350 | 9 | 60 | 200 | 10 | 16 | 65.82 | 16.46 |  |  |
| 10 | 350 | 9 | 60 | 200 | 10 | 16 | 74.52 | 20.76 |  |  |
| 10 | 350 | 9 | 60 | 200 | 10 | 16 | 84.68 | 26.62 |  |  |
| 10 | 350 | 9 | 60 | 200 | 10 | 16 | 92.56 | 31.28 |  |  |
| 10 | 350 | 9 | 60 | 200 | 10 | 16 | 98.13 | 36.08 |  |  |
| 10 | 350 | 9 | 60 | 200 | 10 | 16 | 104.44 | 43.08 |  |  |
| 10 | 350 | 9 | 60 | 200 | 10 | 16 | 103.04 | 46.00 |  |  |
| 10 | 350 | 9 | 60 | 200 | 10 | 16 | 104.33 | 50.00 |  |  |
| 10 | 250 | 4 | 60 | 200 | 10 | 16 | 10.59 | 1.40 | 4 |  |
| 10 | 250 | 4 | 60 | 200 | 10 | 16 | 17.78 | 3.26 |  |  |
| 10 | 250 | 4 | 60 | 200 | 10 | 16 | 24.37 | 6.34 |  |  |
| 10 | 250 | 4 | 60 | 200 | 10 | 16 | 31.56 | 10.20 |  |  |
| 10 | 250 | 4 | 60 | 200 | 10 | 16 | 38.75 | 15.36 |  |  |
| 10 | 250 | 4 | 60 | 200 | 10 | 16 | 44.93 | 20.26 |  |  |
| 10 | 250 | 4 | 60 | 200 | 10 | 16 | 52.13 | 26.22 |  |  |
| 10 | 250 | 4 | 60 | 200 | 10 | 16 | 56.52 | 32.20 |  |  |
| 10 | 250 | 4 | 60 | 200 | 10 | 16 | 59.32 | 37.74 |  |  |
| 10 | 250 | 4 | 60 | 200 | 10 | 16 | 60.72 | 43.64 |  |  |
| 10 | 250 | 4 | 60 | 200 | 10 | 16 | 62.12 | 50.00 |  |  |
| 10 | 250 | 16 | 60 | 200 | 10 | 16 | 11.50 | 0.38 | 5 |  |
| 10 | 250 | 16 | 60 | 200 | 10 | 16 | 23.76 | 0.96 |  |  |
| 10 | 250 | 16 | 60 | 200 | 10 | 16 | 34.35 | 1.94 |  |  |
| 10 | 250 | 16 | 60 | 200 | 10 | 16 | 42.54 | 3.00 |  |  |
| 10 | 250 | 16 | 60 | 200 | 10 | 16 | 49.73 | 4.58 |  |  |
| 10 | 250 | 16 | 60 | 200 | 10 | 16 | 57.32 | 6.42 |  |  |
| 10 | 250 | 16 | 60 | 200 | 10 | 16 | 65.91 | 9.14 |  |  |
| 10 | 250 | 16 | 60 | 200 | 10 | 16 | 72.73 | 11.52 |  |  |
| 10 | 250 | 16 | 60 | 200 | 10 | 16 | 79.69 | 14.22 |  |  |
| 10 | 250 | 16 | 60 | 200 | 10 | 16 | 85.49 | 16.62 |  |  |
| 10 | 250 | 16 | 60 | 200 | 10 | 16 | 93.08 | 20.06 |  |  |
| 10 | 250 | 16 | 60 | 200 | 10 | 16 | 98.87 | 23.14 |  |  |
| 10 | 250 | 16 | 60 | 200 | 10 | 16 | 103.86 | 26.30 |  |  |
| 10 | 250 | 16 | 60 | 200 | 10 | 16 | 109.85 | 29.04 |  |  |
| 10 | 250 | 16 | 60 | 200 | 10 | 16 | 114.93 | 34.60 |  |  |
| 10 | 250 | 16 | 60 | 200 | 10 | 16 | 119.24 | 37.12 |  |  |
| 10 | 250 | 16 | 60 | 200 | 10 | 16 | 119.24 | 41.88 |  |  |
| 10 | 250 | 16 | 60 | 200 | 10 | 16 | 120.04 | 46.02 |  |  |
| 10 | 250 | 16 | 60 | 200 | 10 | 16 | 120.64 | 50.00 |  |  |
| 12 | 300 | 9 | 72 | 200 | 10 | 16 | 11.47 | 0.98 | 6 |  |
| 12 | 300 | 9 | 72 | 200 | 10 | 16 | 22.94 | 2.64 |  |  |
| 12 | 300 | 9 | 72 | 200 | 10 | 16 | 32.95 | 5.12 |  |  |
| 12 | 300 | 9 | 72 | 200 | 10 | 16 | 42.81 | 8.82 |  |  |
| 12 | 300 | 9 | 72 | 200 | 10 | 16 | 51.80 | 12.26 |  |  |
| 12 | 300 | 9 | 72 | 200 | 10 | 16 | 59.02 | 15.88 |  |  |
| 12 | 300 | 9 | 72 | 200 | 10 | 16 | 64.76 | 19.24 |  |  |
| 12 | 300 | 9 | 72 | 200 | 10 | 16 | 70.51 | 23.12 |  |  |
| 12 | 300 | 9 | 72 | 200 | 10 | 16 | 77.57 | 28.32 |  |  |
| 12 | 300 | 9 | 72 | 200 | 10 | 16 | 82.68 | 34.08 |  |  |
| 12 | 300 | 9 | 72 | 200 | 10 | 16 | 87.28 | 38.74 |  |  |
| 12 | 300 | 9 | 72 | 200 | 10 | 16 | 89.90 | 44.74 |  |  |
| 12 | 300 | 9 | 72 | 200 | 10 | 16 | 91.23 | 50.00 |  |  |
| 14 | 350 | 9 | 84 | 200 | 10 | 16 | 12.78 | 0.52 | 7 |  |
| 14 | 350 | 9 | 84 | 200 | 10 | 16 | 20.32 | 1.42 |  |  |
| 14 | 350 | 9 | 84 | 200 | 10 | 16 | 29.01 | 3.26 |  |  |
| 14 | 350 | 9 | 84 | 200 | 10 | 16 | 39.01 | 6.08 |  |  |
| 14 | 350 | 9 | 84 | 200 | 10 | 16 | 48.35 | 9.08 |  |  |
| 14 | 350 | 9 | 84 | 200 | 10 | 16 | 58.03 | 13.06 |  |  |
| 14 | 350 | 9 | 84 | 200 | 10 | 16 | 67.87 | 17.30 |  |  |
| 14 | 350 | 9 | 84 | 200 | 10 | 16 | 75.09 | 20.82 |  |  |
| 14 | 350 | 9 | 84 | 200 | 10 | 16 | 81.98 | 24.62 |  |  |
| 14 | 350 | 9 | 84 | 200 | 10 | 16 | 88.22 | 28.68 |  |  |
| 14 | 350 | 9 | 84 | 200 | 10 | 16 | 93.96 | 33.70 |  |  |
| 14 | 350 | 9 | 84 | 200 | 10 | 16 | 99.22 | 39.08 |  |  |
| 14 | 350 | 9 | 84 | 200 | 10 | 16 | 103.33 | 44.92 |  |  |
| 14 | 350 | 9 | 84 | 200 | 10 | 16 | 105.32 | 50.00 |  |  |
| 10 | 250 | 9 | 30 | 200 | 10 | 16 | 10.42 | 2.14 | 8 |  |
| 10 | 250 | 9 | 30 | 200 | 10 | 16 | 16.32 | 3.84 |  |  |
| 10 | 250 | 9 | 30 | 200 | 10 | 16 | 22.07 | 6.34 |  |  |
| 10 | 250 | 9 | 30 | 200 | 10 | 16 | 27.55 | 9.30 |  |  |
| 10 | 250 | 9 | 30 | 200 | 10 | 16 | 32.62 | 12.16 |  |  |
| 10 | 250 | 9 | 30 | 200 | 10 | 16 | 38.10 | 15.40 |  |  |
| 10 | 250 | 9 | 30 | 200 | 10 | 16 | 44.40 | 18.98 |  |  |
| 10 | 250 | 9 | 30 | 200 | 10 | 16 | 50.70 | 22.92 |  |  |
| 10 | 250 | 9 | 30 | 200 | 10 | 16 | 56.04 | 26.32 |  |  |
| 10 | 250 | 9 | 30 | 200 | 10 | 16 | 62.07 | 30.88 |  |  |
| 10 | 250 | 9 | 30 | 200 | 10 | 16 | 65.76 | 35.10 |  |  |
| 10 | 250 | 9 | 30 | 200 | 10 | 16 | 67.53 | 40.38 |  |  |
| 10 | 250 | 9 | 30 | 200 | 10 | 16 | 67.94 | 44.24 |  |  |
| 10 | 250 | 9 | 30 | 200 | 10 | 16 | 68.47 | 50.00 |  |  |
| 10 | 250 | 9 | 80 | 200 | 10 | 16 | 10.15 | 0.52 | 9 |  |
| 10 | 250 | 9 | 80 | 200 | 10 | 16 | 18.79 | 1.50 |  |  |
| 10 | 250 | 9 | 80 | 200 | 10 | 16 | 25.37 | 2.76 |  |  |
| 10 | 250 | 9 | 80 | 200 | 10 | 16 | 31.95 | 4.36 |  |  |
| 10 | 250 | 9 | 80 | 200 | 10 | 16 | 37.57 | 6.06 |  |  |
| 10 | 250 | 9 | 80 | 200 | 10 | 16 | 42.92 | 8.22 |  |  |
| 10 | 250 | 9 | 80 | 200 | 10 | 16 | 48.81 | 10.72 |  |  |
| 10 | 250 | 9 | 80 | 200 | 10 | 16 | 54.97 | 13.76 |  |  |
| 10 | 250 | 9 | 80 | 200 | 10 | 16 | 60.59 | 16.72 |  |  |
| 10 | 250 | 9 | 80 | 200 | 10 | 16 | 65.80 | 19.86 |  |  |
| 10 | 250 | 9 | 80 | 200 | 10 | 16 | 71.00 | 23.26 |  |  |
| 10 | 250 | 9 | 80 | 200 | 10 | 16 | 76.07 | 27.82 |  |  |
| 10 | 250 | 9 | 80 | 200 | 10 | 16 | 80.04 | 32.58 |  |  |
| 10 | 250 | 9 | 80 | 200 | 10 | 16 | 83.59 | 37.68 |  |  |
| 10 | 250 | 9 | 80 | 200 | 10 | 16 | 86.59 | 44.04 |  |  |
| 10 | 250 | 9 | 80 | 200 | 10 | 16 | 88.22 | 50.00 |  |  |
| 50 | 1200 | 1 | 0 | 150 | 72.5 | 19 | 0.00 | 0.00 | 10 | [7] |
| 50 | 1200 | 1 | 0 | 150 | 72.5 | 19 | 25.80 | 1.02 |  |  |
| 50 | 1200 | 1 | 0 | 150 | 72.5 | 19 | 37.53 | 1.50 |  |  |
| 50 | 1200 | 1 | 0 | 150 | 72.5 | 19 | 51.72 | 2.55 |  |  |
| 50 | 1200 | 1 | 0 | 150 | 72.5 | 19 | 68.02 | 4.01 |  |  |
| 50 | 1200 | 1 | 0 | 150 | 72.5 | 19 | 91.72 | 7.95 |  |  |
| 50 | 1200 | 1 | 0 | 150 | 72.5 | 19 | 110.25 | 14.00 |  |  |
| 50 | 1200 | 1 | 0 | 150 | 72.5 | 19 | 125.73 | 20.00 |  |  |
| 50 | 1200 | 1 | 0 | 150 | 72.5 | 19 | 141.30 | 27.06 |  |  |
| 50 | 1200 | 1 | 0 | 150 | 72.5 | 19 | 157.16 | 36.00 |  |  |
| 50 | 1200 | 1 | 0 | 150 | 72.5 | 19 | 175.93 | 48.00 |  |  |
| 50 | 1200 | 1 | 0 | 150 | 72.5 | 19 | 187.34 | 55.79 |  |  |
| 50 | 1200 | 4 | 150 | 295 | 72.5 | 19 | 0.00 | 0.00 | 11 |  |
| 50 | 1200 | 4 | 150 | 295 | 72.5 | 19 | 12.13 | 0.09 |  |  |
| 50 | 1200 | 4 | 150 | 295 | 72.5 | 19 | 27.29 | 0.38 |  |  |
| 50 | 1200 | 4 | 150 | 295 | 72.5 | 19 | 38.81 | 1.00 |  |  |
| 50 | 1200 | 4 | 150 | 295 | 72.5 | 19 | 49.12 | 1.98 |  |  |
| 50 | 1200 | 4 | 150 | 295 | 72.5 | 19 | 63.07 | 3.01 |  |  |
| 50 | 1200 | 4 | 150 | 295 | 72.5 | 19 | 75.08 | 6.43 |  |  |
| 50 | 1200 | 4 | 150 | 295 | 72.5 | 19 | 83.69 | 12.01 |  |  |
| 50 | 1200 | 4 | 150 | 295 | 72.5 | 19 | 92.18 | 20.00 |  |  |
| 50 | 1200 | 4 | 150 | 295 | 72.5 | 19 | 98.61 | 27.29 |  |  |
| 50 | 1200 | 4 | 150 | 295 | 72.5 | 19 | 103.46 | 34.01 |  |  |
| 50 | 1200 | 4 | 150 | 295 | 72.5 | 19 | 106.74 | 40.50 |  |  |
| 50 | 1200 | 4 | 150 | 295 | 72.5 | 19 | 111.10 | 49.68 |  |  |
| 50 | 1200 | 9 | 150 | 400 | 72.5 | 19 | 0.00 | 0.00 | 12 |  |
| 50 | 1200 | 9 | 150 | 400 | 72.5 | 19 | 19.79 | 0.40 |  |  |
| 50 | 1200 | 9 | 150 | 400 | 72.5 | 19 | 41.68 | 1.08 |  |  |
| 50 | 1200 | 9 | 150 | 400 | 72.5 | 19 | 61.14 | 3.44 |  |  |
| 50 | 1200 | 9 | 150 | 400 | 72.5 | 19 | 85.76 | 11.00 |  |  |
| 50 | 1200 | 9 | 150 | 400 | 72.5 | 19 | 100.72 | 18.76 |  |  |
| 50 | 1200 | 9 | 150 | 400 | 72.5 | 19 | 112.15 | 30.52 |  |  |
| 50 | 1200 | 9 | 150 | 400 | 72.5 | 19 | 122.05 | 44.00 |  |  |
| 50 | 1200 | 9 | 150 | 400 | 72.5 | 19 | 131.94 | 58.24 |  |  |
| 50 | 1200 | 16 | 150 | 560 | 72.5 | 19 | 0.00 | 0.00 | 13 |  |
| 50 | 1200 | 16 | 150 | 560 | 72.5 | 19 | 19.66 | 0.11 |  |  |
| 50 | 1200 | 16 | 150 | 560 | 72.5 | 19 | 41.81 | 0.78 |  |  |
| 50 | 1200 | 16 | 150 | 560 | 72.5 | 19 | 61.46 | 3.53 |  |  |
| 50 | 1200 | 16 | 150 | 560 | 72.5 | 19 | 70.68 | 10.02 |  |  |
| 50 | 1200 | 16 | 150 | 560 | 72.5 | 19 | 77.42 | 17.30 |  |  |
| 50 | 1200 | 16 | 150 | 560 | 72.5 | 19 | 84.52 | 24.81 |  |  |
| 50 | 1200 | 16 | 150 | 560 | 72.5 | 19 | 90.88 | 31.98 |  |  |
| 50 | 1200 | 16 | 150 | 560 | 72.5 | 19 | 97.61 | 40.99 |  |  |
| 50 | 1200 | 16 | 150 | 560 | 72.5 | 19 | 103.43 | 48.38 |  |  |
| 50 | 1200 | 4 | 225 | 300 | 72.5 | 19 | 0.00 | 0.00 | 14 |  |
| 50 | 1200 | 4 | 225 | 300 | 72.5 | 19 | 14.07 | 0.09 |  |  |
| 50 | 1200 | 4 | 225 | 300 | 72.5 | 19 | 32.37 | 0.24 |  |  |
| 50 | 1200 | 4 | 225 | 300 | 72.5 | 19 | 52.91 | 1.14 |  |  |
| 50 | 1200 | 4 | 225 | 300 | 72.5 | 19 | 75.81 | 4.77 |  |  |
| 50 | 1200 | 4 | 225 | 300 | 72.5 | 19 | 89.52 | 11.10 |  |  |
| 50 | 1200 | 4 | 225 | 300 | 72.5 | 19 | 100.86 | 20.01 |  |  |
| 50 | 1200 | 4 | 225 | 300 | 72.5 | 19 | 109.11 | 28.20 |  |  |
| 50 | 1200 | 4 | 225 | 300 | 72.5 | 19 | 117.28 | 38.40 |  |  |
| 50 | 1200 | 4 | 225 | 300 | 72.5 | 19 | 124.32 | 48.00 |  |  |
| 50 | 1200 | 4 | 225 | 300 | 72.5 | 19 | 133.89 | 61.47 |  |  |
| 50 | 1200 | 9 | 225 | 560 | 72.5 | 19 | 0.00 | 0.00 | 15 |  |
| 50 | 1200 | 9 | 225 | 560 | 72.5 | 19 | 13.55 | 0.50 |  |  |
| 50 | 1200 | 9 | 225 | 560 | 72.5 | 19 | 25.25 | 2.02 |  |  |
| 50 | 1200 | 9 | 225 | 560 | 72.5 | 19 | 32.85 | 3.81 |  |  |
| 50 | 1200 | 9 | 225 | 560 | 72.5 | 19 | 42.07 | 5.99 |  |  |
| 50 | 1200 | 9 | 225 | 560 | 72.5 | 19 | 49.92 | 11.03 |  |  |
| 50 | 1200 | 9 | 225 | 560 | 72.5 | 19 | 57.22 | 19.99 |  |  |
| 50 | 1200 | 9 | 225 | 560 | 72.5 | 19 | 65.20 | 34.89 |  |  |
| 50 | 1200 | 9 | 225 | 560 | 72.5 | 19 | 70.68 | 51.02 |  |  |
| 50 | 1200 | 9 | 225 | 560 | 72.5 | 19 | 75.46 | 69.16 |  |  |
| 50 | 1200 | 16 | 225 | 760 | 72.5 | 19 | 0.00 | 0.00 | 16 |  |
| 50 | 1200 | 16 | 225 | 760 | 72.5 | 19 | 11.76 | 0.46 |  |  |
| 50 | 1200 | 16 | 225 | 760 | 72.5 | 19 | 22.87 | 1.22 |  |  |
| 50 | 1200 | 16 | 225 | 760 | 72.5 | 19 | 34.69 | 3.19 |  |  |
| 50 | 1200 | 16 | 225 | 760 | 72.5 | 19 | 44.87 | 8.66 |  |  |
| 50 | 1200 | 16 | 225 | 760 | 72.5 | 19 | 54.33 | 15.28 |  |  |
| 50 | 1200 | 16 | 225 | 760 | 72.5 | 19 | 60.31 | 21.51 |  |  |
| 50 | 1200 | 16 | 225 | 760 | 72.5 | 19 | 67.63 | 30.17 |  |  |
| 50 | 1200 | 16 | 225 | 760 | 72.5 | 19 | 73.31 | 38.00 |  |  |
| 50 | 1200 | 4 | 300 | 350 | 72.5 | 19 | 0.00 | 0.00 | 17 |  |
| 50 | 1200 | 4 | 300 | 350 | 72.5 | 19 | 10.34 | 0.14 |  |  |
| 50 | 1200 | 4 | 300 | 350 | 72.5 | 19 | 26.41 | 0.42 |  |  |
| 50 | 1200 | 4 | 300 | 350 | 72.5 | 19 | 43.87 | 1.02 |  |  |
| 50 | 1200 | 4 | 300 | 350 | 72.5 | 19 | 63.83 | 2.63 |  |  |
| 50 | 1200 | 4 | 300 | 350 | 72.5 | 19 | 82.99 | 9.17 |  |  |
| 50 | 1200 | 4 | 300 | 350 | 72.5 | 19 | 94.79 | 19.99 |  |  |
| 50 | 1200 | 4 | 300 | 350 | 72.5 | 19 | 101.55 | 28.53 |  |  |
| 50 | 1200 | 4 | 300 | 350 | 72.5 | 19 | 106.85 | 35.39 |  |  |
| 50 | 1200 | 4 | 300 | 350 | 72.5 | 19 | 115.46 | 47.99 |  |  |
| 50 | 1200 | 4 | 300 | 350 | 72.5 | 19 | 120.80 | 56.07 |  |  |
| 50 | 1200 | 9 | 300 | 750 | 72.5 | 19 | 0.00 | 0.00 | 18 |  |
| 50 | 1200 | 9 | 300 | 750 | 72.5 | 19 | 10.22 | 0.30 |  |  |
| 50 | 1200 | 9 | 300 | 750 | 72.5 | 19 | 19.84 | 1.05 |  |  |
| 50 | 1200 | 9 | 300 | 750 | 72.5 | 19 | 30.89 | 4.20 |  |  |
| 50 | 1200 | 9 | 300 | 750 | 72.5 | 19 | 41.69 | 8.55 |  |  |
| 50 | 1200 | 9 | 300 | 750 | 72.5 | 19 | 49.72 | 13.28 |  |  |
| 50 | 1200 | 9 | 300 | 750 | 72.5 | 19 | 59.28 | 20.85 |  |  |
| 50 | 1200 | 9 | 300 | 750 | 72.5 | 19 | 65.68 | 28.28 |  |  |
| 50 | 1200 | 9 | 300 | 750 | 72.5 | 19 | 71.87 | 35.48 |  |  |
| 50 | 1200 | 16 | 300 | 1010 | 72.5 | 19 | 0.00 | 0.00 | 19 |  |
| 50 | 1200 | 16 | 300 | 1010 | 72.5 | 19 | 5.17 | 0.40 |  |  |
| 50 | 1200 | 16 | 300 | 1010 | 72.5 | 19 | 10.38 | 0.91 |  |  |
| 50 | 1200 | 16 | 300 | 1010 | 72.5 | 19 | 12.94 | 1.31 |  |  |
| 50 | 1200 | 16 | 300 | 1010 | 72.5 | 19 | 19.14 | 2.02 |  |  |
| 50 | 1200 | 16 | 300 | 1010 | 72.5 | 19 | 21.85 | 2.63 |  |  |
| 50 | 1200 | 16 | 300 | 1010 | 72.5 | 19 | 28.39 | 4.14 |  |  |
| 50 | 1200 | 16 | 300 | 1010 | 72.5 | 19 | 33.22 | 6.16 |  |  |
| 50 | 1200 | 16 | 300 | 1010 | 72.5 | 19 | 39.43 | 9.90 |  |  |
| 50 | 1200 | 16 | 300 | 1010 | 72.5 | 19 | 43.46 | 13.23 |  |  |
| 50 | 1200 | 16 | 300 | 1010 | 72.5 | 19 | 47.34 | 16.77 |  |  |
| 50 | 1200 | 16 | 300 | 1010 | 72.5 | 19 | 49.67 | 20.00 |  |  |
| 50 | 1200 | 16 | 300 | 1010 | 72.5 | 19 | 51.54 | 23.63 |  |  |
| 50 | 1600 | 1 | 0 | 150 | 72.5 | 19 | 0.00 | 0.00 | 20 |  |
| 50 | 1600 | 1 | 0 | 150 | 72.5 | 19 | 46.91 | 0.20 |  |  |
| 50 | 1600 | 1 | 0 | 150 | 72.5 | 19 | 94.77 | 0.59 |  |  |
| 50 | 1600 | 1 | 0 | 150 | 72.5 | 19 | 146.28 | 4.02 |  |  |
| 50 | 1600 | 1 | 0 | 150 | 72.5 | 19 | 168.89 | 14.00 |  |  |
| 50 | 1600 | 1 | 0 | 150 | 72.5 | 19 | 187.65 | 24.80 |  |  |
| 50 | 1600 | 1 | 0 | 150 | 72.5 | 19 | 203.83 | 33.62 |  |  |
| 50 | 1600 | 1 | 0 | 150 | 72.5 | 19 | 225.19 | 44.81 |  |  |
| 50 | 1600 | 1 | 0 | 150 | 72.5 | 19 | 258.02 | 62.00 |  |  |
| 50 | 1600 | 1 | 0 | 150 | 72.5 | 19 | 279.83 | 73.08 |  |  |
| 50 | 1600 | 4 | 150 | 300 | 72.5 | 19 | 0.00 | 0.00 | 21 |  |
| 50 | 1600 | 4 | 150 | 300 | 72.5 | 19 | 23.46 | 0.24 |  |  |
| 50 | 1600 | 4 | 150 | 300 | 72.5 | 19 | 50.72 | 0.63 |  |  |
| 50 | 1600 | 4 | 150 | 300 | 72.5 | 19 | 70.37 | 0.81 |  |  |
| 50 | 1600 | 4 | 150 | 300 | 72.5 | 19 | 93.73 | 1.38 |  |  |
| 50 | 1600 | 4 | 150 | 300 | 72.5 | 19 | 117.28 | 3.69 |  |  |
| 50 | 1600 | 4 | 150 | 300 | 72.5 | 19 | 134.93 | 9.96 |  |  |
| 50 | 1600 | 4 | 150 | 300 | 72.5 | 19 | 152.47 | 23.01 |  |  |
| 50 | 1600 | 4 | 150 | 300 | 72.5 | 19 | 164.20 | 34.29 |  |  |
| 50 | 1600 | 4 | 150 | 300 | 72.5 | 19 | 175.93 | 45.00 |  |  |
| 50 | 1600 | 4 | 150 | 300 | 72.5 | 19 | 187.68 | 56.73 |  |  |
| 50 | 1600 | 9 | 150 | 400 | 72.5 | 19 | 0.00 | 0.00 | 22 |  |
| 50 | 1600 | 9 | 150 | 400 | 72.5 | 19 | 34.59 | 0.04 |  |  |
| 50 | 1600 | 9 | 150 | 400 | 72.5 | 19 | 52.78 | 0.08 |  |  |
| 50 | 1600 | 9 | 150 | 400 | 72.5 | 19 | 77.93 | 0.28 |  |  |
| 50 | 1600 | 9 | 150 | 400 | 72.5 | 19 | 103.63 | 1.08 |  |  |
| 50 | 1600 | 9 | 150 | 400 | 72.5 | 19 | 137.55 | 5.56 |  |  |
| 50 | 1600 | 9 | 150 | 400 | 72.5 | 19 | 155.03 | 10.52 |  |  |
| 50 | 1600 | 9 | 150 | 400 | 72.5 | 19 | 170.60 | 16.68 |  |  |
| 50 | 1600 | 9 | 150 | 400 | 72.5 | 19 | 181.42 | 23.00 |  |  |
| 50 | 1600 | 9 | 150 | 400 | 72.5 | 19 | 191.31 | 29.32 |  |  |
| 50 | 1600 | 9 | 150 | 400 | 72.5 | 19 | 200.94 | 35.84 |  |  |
| 50 | 1600 | 4 | 225 | 320 | 72.5 | 19 | 0.00 | 0.00 | 23 |  |
| 50 | 1600 | 4 | 225 | 320 | 72.5 | 19 | 12.37 | 0.10 |  |  |
| 50 | 1600 | 4 | 225 | 320 | 72.5 | 19 | 29.48 | 0.22 |  |  |
| 50 | 1600 | 4 | 225 | 320 | 72.5 | 19 | 52.57 | 0.61 |  |  |
| 50 | 1600 | 4 | 225 | 320 | 72.5 | 19 | 63.91 | 0.80 |  |  |
| 50 | 1600 | 4 | 225 | 320 | 72.5 | 19 | 79.48 | 1.31 |  |  |
| 50 | 1600 | 4 | 225 | 320 | 72.5 | 19 | 92.77 | 4.51 |  |  |
| 50 | 1600 | 4 | 225 | 320 | 72.5 | 19 | 102.59 | 7.55 |  |  |
| 50 | 1600 | 4 | 225 | 320 | 72.5 | 19 | 116.00 | 13.79 |  |  |
| 50 | 1600 | 4 | 225 | 320 | 72.5 | 19 | 129.64 | 24.48 |  |  |
| 50 | 1600 | 4 | 225 | 320 | 72.5 | 19 | 134.01 | 30.02 |  |  |
| 50 | 1600 | 4 | 225 | 320 | 72.5 | 19 | 138.13 | 36.51 |  |  |
| 50 | 1600 | 4 | 225 | 320 | 72.5 | 19 | 143.80 | 45.60 |  |  |
| 50 | 1600 | 9 | 225 | 550 | 72.5 | 19 | 0.00 | 0.00 | 24 |  |
| 50 | 1600 | 9 | 225 | 550 | 72.5 | 19 | 17.45 | 0.50 |  |  |
| 50 | 1600 | 9 | 225 | 550 | 72.5 | 19 | 35.04 | 1.38 |  |  |
| 50 | 1600 | 9 | 225 | 550 | 72.5 | 19 | 52.34 | 3.19 |  |  |
| 50 | 1600 | 9 | 225 | 550 | 72.5 | 19 | 66.79 | 6.05 |  |  |
| 50 | 1600 | 9 | 225 | 550 | 72.5 | 19 | 78.51 | 10.01 |  |  |
| 50 | 1600 | 9 | 225 | 550 | 72.5 | 19 | 87.24 | 14.03 |  |  |
| 50 | 1600 | 9 | 225 | 550 | 72.5 | 19 | 98.76 | 21.29 |  |  |
| 50 | 1600 | 9 | 225 | 550 | 72.5 | 19 | 108.17 | 30.31 |  |  |
| 50 | 1600 | 9 | 225 | 550 | 72.5 | 19 | 116.90 | 39.99 |  |  |
| 50 | 1600 | 9 | 225 | 550 | 72.5 | 19 | 126.18 | 52.20 |  |  |
| 50 | 1600 | 4 | 300 | 400 | 72.5 | 19 | 0.00 | 0.00 | 25 |  |
| 50 | 1600 | 4 | 300 | 400 | 72.5 | 19 | 19.79 | 0.16 |  |  |
| 50 | 1600 | 4 | 300 | 400 | 72.5 | 19 | 34.94 | 0.36 |  |  |
| 50 | 1600 | 4 | 300 | 400 | 72.5 | 19 | 52.78 | 1.80 |  |  |
| 50 | 1600 | 4 | 300 | 400 | 72.5 | 19 | 68.19 | 5.20 |  |  |
| 50 | 1600 | 4 | 300 | 400 | 72.5 | 19 | 85.76 | 13.80 |  |  |
| 50 | 1600 | 4 | 300 | 400 | 72.5 | 19 | 99.49 | 24.44 |  |  |
| 50 | 1600 | 4 | 300 | 400 | 72.5 | 19 | 108.85 | 36.00 |  |  |
| 50 | 1600 | 4 | 300 | 400 | 72.5 | 19 | 118.75 | 50.00 |  |  |
| 50 | 1600 | 4 | 300 | 400 | 72.5 | 19 | 127.82 | 63.64 |  |  |
| 50 | 1600 | 9 | 300 | 700 | 72.5 | 19 | 0.00 | 0.00 | 26 |  |
| 50 | 1600 | 9 | 300 | 700 | 72.5 | 19 | 10.77 | 0.63 |  |  |
| 50 | 1600 | 9 | 300 | 700 | 72.5 | 19 | 19.31 | 1.33 |  |  |
| 50 | 1600 | 9 | 300 | 700 | 72.5 | 19 | 30.16 | 2.03 |  |  |
| 50 | 1600 | 9 | 300 | 700 | 72.5 | 19 | 41.86 | 4.41 |  |  |
| 50 | 1600 | 9 | 300 | 700 | 72.5 | 19 | 53.85 | 10.99 |  |  |
| 50 | 1600 | 9 | 300 | 700 | 72.5 | 19 | 64.66 | 21.28 |  |  |
| 50 | 1600 | 9 | 300 | 700 | 72.5 | 19 | 70.01 | 30.03 |  |  |
| 50 | 1600 | 9 | 300 | 700 | 72.5 | 19 | 75.40 | 42.00 |  |  |
| 50 | 1600 | 9 | 300 | 700 | 72.5 | 19 | 83.13 | 59.85 |  |  |
| 50 | 2000 | 1 | 0 | 150 | 72.5 | 19 | 0.00 | 0.00 | 27 |  |
| 50 | 2000 | 1 | 0 | 150 | 72.5 | 19 | 46.91 | 0.11 |  |  |
| 50 | 2000 | 1 | 0 | 150 | 72.5 | 19 | 96.45 | 0.35 |  |  |
| 50 | 2000 | 1 | 0 | 150 | 72.5 | 19 | 140.74 | 0.60 |  |  |
| 50 | 2000 | 1 | 0 | 150 | 72.5 | 19 | 186.25 | 3.83 |  |  |
| 50 | 2000 | 1 | 0 | 150 | 72.5 | 19 | 215.80 | 10.01 |  |  |
| 50 | 2000 | 1 | 0 | 150 | 72.5 | 19 | 239.26 | 16.01 |  |  |
| 50 | 2000 | 1 | 0 | 150 | 72.5 | 19 | 262.72 | 22.50 |  |  |
| 50 | 2000 | 1 | 0 | 150 | 72.5 | 19 | 281.64 | 27.98 |  |  |
| 50 | 2000 | 4 | 150 | 300 | 72.5 | 19 | 0.00 | 0.00 | 28 |  |
| 50 | 2000 | 4 | 150 | 300 | 72.5 | 19 | 34.54 | 0.09 |  |  |
| 50 | 2000 | 4 | 150 | 300 | 72.5 | 19 | 75.18 | 0.63 |  |  |
| 50 | 2000 | 4 | 150 | 300 | 72.5 | 19 | 105.56 | 2.49 |  |  |
| 50 | 2000 | 4 | 150 | 300 | 72.5 | 19 | 129.89 | 5.76 |  |  |
| 50 | 2000 | 4 | 150 | 300 | 72.5 | 19 | 152.47 | 11.01 |  |  |
| 50 | 2000 | 4 | 150 | 300 | 72.5 | 19 | 175.93 | 18.00 |  |  |
| 50 | 2000 | 4 | 150 | 300 | 72.5 | 19 | 191.67 | 23.97 |  |  |
| 50 | 2000 | 4 | 150 | 300 | 72.5 | 19 | 205.25 | 32.01 |  |  |
| 50 | 2000 | 4 | 150 | 300 | 72.5 | 19 | 216.98 | 39.99 |  |  |
| 50 | 2000 | 4 | 150 | 300 | 72.5 | 19 | 229.69 | 49.20 |  |  |
| 50 | 2000 | 9 | 150 | 450 | 72.5 | 19 | 0.00 | 0.00 | 29 |  |
| 50 | 2000 | 9 | 150 | 450 | 72.5 | 19 | 28.21 | 0.18 |  |  |
| 50 | 2000 | 9 | 150 | 450 | 72.5 | 19 | 50.03 | 0.86 |  |  |
| 50 | 2000 | 9 | 150 | 450 | 72.5 | 19 | 76.93 | 3.02 |  |  |
| 50 | 2000 | 9 | 150 | 450 | 72.5 | 19 | 99.93 | 5.27 |  |  |
| 50 | 2000 | 9 | 150 | 450 | 72.5 | 19 | 121.45 | 9.99 |  |  |
| 50 | 2000 | 9 | 150 | 450 | 72.5 | 19 | 137.35 | 20.79 |  |  |
| 50 | 2000 | 9 | 150 | 450 | 72.5 | 19 | 143.35 | 34.02 |  |  |
| 50 | 2000 | 9 | 150 | 450 | 72.5 | 19 | 148.56 | 52.02 |  |  |
| 50 | 2000 | 9 | 150 | 450 | 72.5 | 19 | 151.17 | 64.98 |  |  |
| 50 | 2000 | 9 | 150 | 450 | 72.5 | 19 | 155.70 | 85.64 |  |  |
| 50 | 2000 | 4 | 225 | 350 | 72.5 | 19 | 0.00 | 0.00 | 30 |  |
| 50 | 2000 | 4 | 225 | 350 | 72.5 | 19 | 25.85 | 0.60 |  |  |
| 50 | 2000 | 4 | 225 | 350 | 72.5 | 19 | 57.42 | 1.61 |  |  |
| 50 | 2000 | 4 | 225 | 350 | 72.5 | 19 | 81.86 | 3.01 |  |  |
| 50 | 2000 | 4 | 225 | 350 | 72.5 | 19 | 109.02 | 9.35 |  |  |
| 50 | 2000 | 4 | 225 | 350 | 72.5 | 19 | 129.25 | 19.01 |  |  |
| 50 | 2000 | 4 | 225 | 350 | 72.5 | 19 | 150.79 | 31.01 |  |  |
| 50 | 2000 | 4 | 225 | 350 | 72.5 | 19 | 179.32 | 49.67 |  |  |
| 50 | 2000 | 4 | 225 | 350 | 72.5 | 19 | 193.88 | 61.99 |  |  |
| 50 | 2000 | 4 | 225 | 350 | 72.5 | 19 | 206.80 | 75.99 |  |  |
| 50 | 2000 | 4 | 225 | 350 | 72.5 | 19 | 217.59 | 89.01 |  |  |
| 50 | 2000 | 9 | 225 | 540 | 72.5 | 19 | 0.00 | 0.00 | 31 |  |
| 50 | 2000 | 9 | 225 | 540 | 72.5 | 19 | 21.24 | 0.70 |  |  |
| 50 | 2000 | 9 | 225 | 540 | 72.5 | 19 | 46.09 | 2.21 |  |  |
| 50 | 2000 | 9 | 225 | 540 | 72.5 | 19 | 65.77 | 4.37 |  |  |
| 50 | 2000 | 9 | 225 | 540 | 72.5 | 19 | 81.45 | 7.02 |  |  |
| 50 | 2000 | 9 | 225 | 540 | 72.5 | 19 | 91.86 | 9.67 |  |  |
| 50 | 2000 | 9 | 225 | 540 | 72.5 | 19 | 104.98 | 14.31 |  |  |
| 50 | 2000 | 9 | 225 | 540 | 72.5 | 19 | 116.74 | 21.38 |  |  |
| 50 | 2000 | 9 | 225 | 540 | 72.5 | 19 | 124.89 | 30.02 |  |  |
| 50 | 2000 | 9 | 225 | 540 | 72.5 | 19 | 132.13 | 38.99 |  |  |
| 50 | 2000 | 9 | 225 | 540 | 72.5 | 19 | 138.16 | 47.57 |  |  |
| 30 | 750 | 1 | 0 | 100 | 72.5 | 19 | 0.00 | 0.00 | 32 |  |
| 30 | 750 | 1 | 0 | 100 | 72.5 | 19 | 21.11 | 0.10 |  |  |
| 30 | 750 | 1 | 0 | 100 | 72.5 | 19 | 53.94 | 0.32 |  |  |
| 30 | 750 | 1 | 0 | 100 | 72.5 | 19 | 84.44 | 0.60 |  |  |
| 30 | 750 | 1 | 0 | 100 | 72.5 | 19 | 107.88 | 2.94 |  |  |
| 30 | 750 | 1 | 0 | 100 | 72.5 | 19 | 130.89 | 10.00 |  |  |
| 30 | 750 | 1 | 0 | 100 | 72.5 | 19 | 145.67 | 16.00 |  |  |
| 30 | 750 | 1 | 0 | 100 | 72.5 | 19 | 158.99 | 21.64 |  |  |
| 30 | 750 | 1 | 0 | 100 | 72.5 | 19 | 175.22 | 29.00 |  |  |
| 30 | 750 | 1 | 0 | 100 | 72.5 | 19 | 189.37 | 37.18 |  |  |
| 30 | 750 | 4 | 90 | 150 | 72.5 | 19 | 0.00 | 0.00 | 33 |  |
| 30 | 750 | 4 | 90 | 150 | 72.5 | 19 | 32.84 | 0.41 |  |  |
| 30 | 750 | 4 | 90 | 150 | 72.5 | 19 | 70.84 | 1.20 |  |  |
| 30 | 750 | 4 | 90 | 150 | 72.5 | 19 | 112.59 | 2.51 |  |  |
| 30 | 750 | 4 | 90 | 150 | 72.5 | 19 | 150.12 | 3.50 |  |  |
| 30 | 750 | 4 | 90 | 150 | 72.5 | 19 | 189.62 | 6.60 |  |  |
| 30 | 750 | 4 | 90 | 150 | 72.5 | 19 | 225.19 | 13.50 |  |  |
| 30 | 750 | 4 | 90 | 150 | 72.5 | 19 | 255.68 | 23.40 |  |  |
| 30 | 750 | 4 | 90 | 150 | 72.5 | 19 | 276.79 | 31.01 |  |  |
| 30 | 750 | 4 | 90 | 150 | 72.5 | 19 | 304.94 | 41.30 |  |  |
| 30 | 750 | 4 | 90 | 150 | 72.5 | 19 | 323.24 | 48.74 |  |  |
| 30 | 750 | 9 | 90 | 250 | 72.5 | 19 | 0.00 | 0.00 | 34 |  |
| 30 | 750 | 9 | 90 | 250 | 72.5 | 19 | 28.71 | 0.60 |  |  |
| 30 | 750 | 9 | 90 | 250 | 72.5 | 19 | 54.04 | 1.00 |  |  |
| 30 | 750 | 9 | 90 | 250 | 72.5 | 19 | 80.97 | 2.55 |  |  |
| 30 | 750 | 9 | 90 | 250 | 72.5 | 19 | 114.84 | 5.00 |  |  |
| 30 | 750 | 9 | 90 | 250 | 72.5 | 19 | 142.53 | 10.80 |  |  |
| 30 | 750 | 9 | 90 | 250 | 72.5 | 19 | 168.89 | 22.00 |  |  |
| 30 | 750 | 9 | 90 | 250 | 72.5 | 19 | 185.78 | 31.00 |  |  |
| 30 | 750 | 9 | 90 | 250 | 72.5 | 19 | 210.35 | 44.68 |  |  |
| 30 | 750 | 16 | 90 | 350 | 72.5 | 19 | 0.00 | 0.00 | 35 |  |
| 30 | 750 | 16 | 90 | 350 | 72.5 | 19 | 17.23 | 0.32 |  |  |
| 30 | 750 | 16 | 90 | 350 | 72.5 | 19 | 38.78 | 0.84 |  |  |
| 30 | 750 | 16 | 90 | 350 | 72.5 | 19 | 60.32 | 1.89 |  |  |
| 30 | 750 | 16 | 90 | 350 | 72.5 | 19 | 75.94 | 3.22 |  |  |
| 30 | 750 | 16 | 90 | 350 | 72.5 | 19 | 94.78 | 4.80 |  |  |
| 30 | 750 | 16 | 90 | 350 | 72.5 | 19 | 116.33 | 7.00 |  |  |
| 30 | 750 | 16 | 90 | 350 | 72.5 | 19 | 136.06 | 11.66 |  |  |
| 30 | 750 | 16 | 90 | 350 | 72.5 | 19 | 150.79 | 17.01 |  |  |
| 30 | 750 | 16 | 90 | 350 | 72.5 | 19 | 163.72 | 23.49 |  |  |
| 30 | 750 | 16 | 90 | 350 | 72.5 | 19 | 180.95 | 32.52 |  |  |
| 30 | 750 | 16 | 90 | 350 | 72.5 | 19 | 196.54 | 41.02 |  |  |
| 26 | 750 | 4 | 120 | 200 | 72.5 | 19 | 0.00 | 0.00 | 36 |  |
| 26 | 750 | 4 | 120 | 200 | 72.5 | 19 | 15.83 | 0.06 |  |  |
| 26 | 750 | 4 | 120 | 200 | 72.5 | 19 | 32.41 | 0.12 |  |  |
| 26 | 750 | 4 | 120 | 200 | 72.5 | 19 | 54.94 | 1.56 |  |  |
| 26 | 750 | 4 | 120 | 200 | 72.5 | 19 | 73.89 | 3.00 |  |  |
| 26 | 750 | 4 | 120 | 200 | 72.5 | 19 | 90.86 | 4.90 |  |  |
| 26 | 750 | 4 | 120 | 200 | 72.5 | 19 | 105.56 | 8.00 |  |  |
| 26 | 750 | 4 | 120 | 200 | 72.5 | 19 | 120.62 | 12.68 |  |  |
| 26 | 750 | 4 | 120 | 200 | 72.5 | 19 | 137.22 | 19.50 |  |  |
| 26 | 750 | 4 | 120 | 200 | 72.5 | 19 | 151.56 | 26.18 |  |  |
| 26 | 750 | 4 | 120 | 200 | 72.5 | 19 | 163.61 | 32.20 |  |  |
| 26 | 750 | 4 | 120 | 200 | 72.5 | 19 | 175.40 | 38.78 |  |  |
| 26 | 750 | 9 | 120 | 300 | 72.5 | 19 | 0.00 | 0.00 | 37 |  |
| 26 | 750 | 9 | 120 | 300 | 72.5 | 19 | 11.73 | 0.21 |  |  |
| 26 | 750 | 9 | 120 | 300 | 72.5 | 19 | 27.91 | 0.69 |  |  |
| 26 | 750 | 9 | 120 | 300 | 72.5 | 19 | 39.88 | 2.49 |  |  |
| 26 | 750 | 9 | 120 | 300 | 72.5 | 19 | 51.07 | 4.77 |  |  |
| 26 | 750 | 9 | 120 | 300 | 72.5 | 19 | 65.68 | 8.01 |  |  |
| 26 | 750 | 9 | 120 | 300 | 72.5 | 19 | 74.04 | 10.98 |  |  |
| 26 | 750 | 9 | 120 | 300 | 72.5 | 19 | 89.14 | 18.21 |  |  |
| 26 | 750 | 9 | 120 | 300 | 72.5 | 19 | 100.62 | 26.07 |  |  |
| 26 | 750 | 9 | 120 | 300 | 72.5 | 19 | 107.90 | 32.79 |  |  |
| 26 | 750 | 9 | 120 | 300 | 72.5 | 19 | 117.28 | 42.99 |  |  |
| 26 | 750 | 9 | 120 | 300 | 72.5 | 19 | 127.23 | 54.06 |  |  |
| 26 | 750 | 16 | 120 | 430 | 72.5 | 19 | 0.00 | 0.00 | 38 |  |
| 26 | 750 | 16 | 120 | 430 | 72.5 | 19 | 9.13 | 0.09 |  |  |
| 26 | 750 | 16 | 120 | 430 | 72.5 | 19 | 17.21 | 0.39 |  |  |
| 26 | 750 | 16 | 120 | 430 | 72.5 | 19 | 32.29 | 1.68 |  |  |
| 26 | 750 | 16 | 120 | 430 | 72.5 | 19 | 45.50 | 4.77 |  |  |
| 26 | 750 | 16 | 120 | 430 | 72.5 | 19 | 62.36 | 9.68 |  |  |
| 26 | 750 | 16 | 120 | 430 | 72.5 | 19 | 70.79 | 14.49 |  |  |
| 26 | 750 | 16 | 120 | 430 | 72.5 | 19 | 79.57 | 21.46 |  |  |
| 26 | 750 | 16 | 120 | 430 | 72.5 | 19 | 82.21 | 31.00 |  |  |
| 26 | 750 | 16 | 120 | 430 | 72.5 | 19 | 93.71 | 38.61 |  |  |
| 26 | 750 | 16 | 120 | 430 | 72.5 | 19 | 100.47 | 48.50 |  |  |
| 26 | 750 | 16 | 120 | 430 | 72.5 | 19 | 107.33 | 59.30 |  |  |
| 20 | 750 | 4 | 60 | 150 | 72.5 | 19 | 0.00 | 0.00 | 39 |  |
| 20 | 750 | 4 | 60 | 150 | 72.5 | 19 | 23.46 | 0.30 |  |  |
| 20 | 750 | 4 | 60 | 150 | 72.5 | 19 | 49.26 | 0.84 |  |  |
| 20 | 750 | 4 | 60 | 150 | 72.5 | 19 | 79.75 | 2.00 |  |  |
| 20 | 750 | 4 | 60 | 150 | 72.5 | 19 | 101.52 | 4.25 |  |  |
| 20 | 750 | 4 | 60 | 150 | 72.5 | 19 | 133.70 | 9.00 |  |  |
| 20 | 750 | 4 | 60 | 150 | 72.5 | 19 | 157.68 | 16.17 |  |  |
| 20 | 750 | 4 | 60 | 150 | 72.5 | 19 | 187.65 | 30.50 |  |  |
| 20 | 750 | 4 | 60 | 150 | 72.5 | 19 | 211.11 | 44.00 |  |  |
| 20 | 750 | 4 | 60 | 150 | 72.5 | 19 | 226.69 | 52.70 |  |  |
| 20 | 750 | 9 | 60 | 200 | 72.5 | 19 | 0.00 | 0.00 | 40 |  |
| 20 | 750 | 9 | 60 | 200 | 72.5 | 19 | 21.11 | 0.30 |  |  |
| 20 | 750 | 9 | 60 | 200 | 72.5 | 19 | 44.86 | 0.64 |  |  |
| 20 | 750 | 9 | 60 | 200 | 72.5 | 19 | 63.33 | 1.00 |  |  |
| 20 | 750 | 9 | 60 | 200 | 72.5 | 19 | 81.81 | 2.24 |  |  |
| 20 | 750 | 9 | 60 | 200 | 72.5 | 19 | 102.92 | 5.00 |  |  |
| 20 | 750 | 9 | 60 | 200 | 72.5 | 19 | 118.96 | 8.10 |  |  |
| 20 | 750 | 9 | 60 | 200 | 72.5 | 19 | 139.86 | 14.00 |  |  |
| 20 | 750 | 9 | 60 | 200 | 72.5 | 19 | 159.07 | 21.52 |  |  |
| 20 | 750 | 9 | 60 | 200 | 72.5 | 19 | 182.08 | 32.00 |  |  |
| 20 | 750 | 9 | 60 | 200 | 72.5 | 19 | 200.56 | 41.80 |  |  |
| 20 | 750 | 9 | 60 | 200 | 72.5 | 19 | 221.67 | 52.00 |  |  |
| 20 | 750 | 9 | 60 | 200 | 72.5 | 19 | 240.03 | 61.90 |  |  |
| 20 | 750 | 16 | 60 | 270 | 72.5 | 19 | 0.00 | 0.00 | 41 |  |
| 20 | 750 | 16 | 60 | 270 | 72.5 | 19 | 24.62 | 0.57 |  |  |
| 20 | 750 | 16 | 60 | 270 | 72.5 | 19 | 52.13 | 1.00 |  |  |
| 20 | 750 | 16 | 60 | 270 | 72.5 | 19 | 72.72 | 2.03 |  |  |
| 20 | 750 | 16 | 60 | 270 | 72.5 | 19 | 92.67 | 4.00 |  |  |
| 20 | 750 | 16 | 60 | 270 | 72.5 | 19 | 118.79 | 8.51 |  |  |
| 20 | 750 | 16 | 60 | 270 | 72.5 | 19 | 139.00 | 14.50 |  |  |
| 20 | 750 | 16 | 60 | 270 | 72.5 | 19 | 159.27 | 22.01 |  |  |
| 20 | 750 | 16 | 60 | 270 | 72.5 | 19 | 170.49 | 26.60 |  |  |
| 20 | 750 | 16 | 60 | 270 | 72.5 | 19 | 188.23 | 34.99 |  |  |
| 20 | 750 | 16 | 60 | 270 | 72.5 | 19 | 202.71 | 42.01 |  |  |
| 20 | 750 | 16 | 60 | 270 | 72.5 | 19 | 220.09 | 51.00 |  |  |
| 20 | 750 | 16 | 60 | 270 | 72.5 | 19 | 241.69 | 61.88 |  |  |
